# Supplementary material for: Simplified clinical algorithm for identifying patients eligible for immediate initiation of antiretroviral therapy for HIV (SLATE): protocol for a randomised evaluation
Source: BMJ Open. 2017 May 28;7(5):e016340. doi: 10.1136/bmjopen-2017-016340 (PMC5726128; doi:10.1136/bmjopen-2017-016340)
Supplement: Supplementary material 1 [file bmjopen-2017-016340supp001.pdf]

SIMPLIFIED ALGORITHM FOR TREATMENT ELIGIBILITY (SLATE): SCREENS

Symptom report

1. Does patient have a cough? Yes/No
2. Does patient have a fever? Yes/No
3. Does patient report having night sweats? Yes/No
4. Does patient report losing weight? Yes/No
5. Does patient have a continuous headache that hasn't gone away for at least two days? (If headache is persistent or continuous, answer "yes." If occasional or temporary, answer "no.") Yes/No
6. Does patient report any other symptoms of illness? Yes/No

   If yes, what other symptoms? (Specify)

   If yes, do any of the other symptoms indicate that further consultation is needed before starting ART? Yes/No
7. On the basis of symptoms, should this patient be referred for further care before starting ART? Yes/No

Medical history

1. Have you been on ART before? Yes/No

   If yes, when did you start taking ART before? (Record)

   If yes, when did you stop taking ART before? (Record)
2. Are you currently on TB treatment? Yes/No

   If yes, how many weeks ago did you start taking TB treatment? (Record)

   If patient started taking TB treatment 1 or 2 weeks ago, how many days ago did she or he start TB treatment? (Record)
3. Have you been told that you have other diseases or health problems besides HIV? Yes/No

   If yes, what other diseases or conditions do you have? (Specify)

   If yes, do any of the diseases or conditions suggest further consultation is needed? Yes/No
4. Are you currently taking any anti-epilepsy medication? Yes/No
5. Are you currently taking warfarin? Yes/No
6. Are you currently taking any other medications? Yes/No

   If yes, what other medications are you currently taking? (Specify)

   If yes, do any of the medications suggest that further consultation is needed? Yes/No
7. Do you currently take any recreational drugs, such as smoking (bhang/ dagga/ weed) or using pills or injectables? Yes/No

NOT FOR USE IN PRACTICE

8. Do you currently drink more alcohol than you think you should? Yes/No
9. On the basis of medical history, should this patient be referred for further care before starting ART? Yes/No

Physical examination

1. Temperature (Record)
2. Systolic blood pressure (Record)
3. Diastolic blood pressure (Record)
4. Weight (kg) (Record)
5. Did the patient report symptoms that should be examined? Yes/No

   If yes, after examination, do any symptoms suggest that further consultation is needed? Yes/No

   If yes, which symptoms should be investigated further? (Specify)
6. Does the patient have other conditions for which further consultation is needed? Yes/No

   If yes, what are the other conditions? (Specify)
8. On the basis of the physical exam, should should this patient be referred for further care before starting ART? Yes/No

Readiness assessment

1. If you should start ART today, how ready are you?
   Not ready
   Thinking about trying, maybe in the next month or so
   Almost ready, maybe this week
   Ready today
2. How will you remember to take your medication every day?
   Mobile phone reminder
   Alarm on a clock or watch
   Ask someone to remind me
   Use a calendar or diary
   Take my tablets at the same time as I do something else every day (like brushing my teeth)
   No reminders, I'll just remember
   Other way (specify)
3. Is there anything that will stop you from taking your tablets every day? Yes/No
4. Did the patient raise any issues or concerns that lead you to think that ART should be delayed?

   If yes, what were these issues or concerns and how did you respond?
5. On the basis of the readiness assessment, should this patient be referred for further care before starting ART? Yes/No

NOT FOR USE IN PRACTICE
