# Supplementary material for: Simplified clinical algorithm for identifying patients eligible for immediate initiation of antiretroviral therapy for HIV (SLATE): protocol for a randomised evaluation
Source: BMJ Open. 2017 May 28;7(5):e016340. doi: 10.1136/bmjopen-2017-016340 (PMC5726128; doi:10.1136/bmjopen-2017-016340)
Supplement: Author's manuscript [file bmjopen-2017-016340.draft_revisions.pdf]

BMJ Open

Simplified clinical algorithm for identifying patients eligible for immediate initiation of antiretroviral therapy for HIV (SLATE): protocol for a randomized evaluation

Journal:	*BMJ Open*
Manuscript ID	bmjopen-2017-016340
Article Type:	Protocol
Date Submitted by the Author:	08-Feb-2017

Complete List of Authors:	Rosen, Sydney; Boston University School of Public Health, Center for Global Health and Development; University of the Witwatersrand Faculty of Health Sciences, Health Economics and Epidemiology Research Office<br>Fox, Matt; Boston University, Epidemiology and Global Health<br>Larson, Bruce; Boston University, School of Public Health<br>Brennan, Alana; Boston University<br>Maskew, Mhairi; University of the Witwatersrand, Health Economics and Epidemiology Research Office<br>Tsikhutsu, Isaac; Kenya Medical Research Institute, Walter Reed Projects<br>Bii, Margaret; Kenya Medical Research Institute, Walter Reed Projects<br>Ehrenkranz, Peter; Bill & Melinda Gates Foundation<br>Venter, WD Francois; University of the Witwatersrand, Wits RHI

**Primary Subject Heading**:	HIV/AIDS
Secondary Subject Heading:	Global health, Health services research
Keywords:	Antiretroviral therapy, Treatment initiation, South Africa, Kenya, Protocols & guidelines < HEALTH SERVICES ADMINISTRATION & MANAGEMENT, Randomized trial

SCHOLARONE™
Manuscripts

Simplified clinical algorithm for identifying patients eligible for immediate initiation of antiretroviral therapy for HIV (SLATE): protocol for a randomized evaluation

Rosen S[1,2*], Fox MP[1,2,3], Larson BA[1], Brennan AT[1,2], Maskew M[2], Tsikhutsu I[4], Bii M[4], Ehrenkranz PD[5], Venter WDF[6]

[1]Department of Global Health, Boston University School of Public Health, Boston, Massachusetts, United States of America

[2]Health Economics and Epidemiology Research Office, Department of Internal Medicine, School of Clinical Medicine, Faculty of Health Sciences, University of the Witwatersrand, Johannesburg, South Africa

[3]Department of Epidemiology, Boston University School of Public Health, Boston, Massachusetts, United States of America

[4]Kenya Medical Research Institute/Walter Reed Project HIV Program, Kericho, Kenya

[5]Bill & Melinda Gates Foundation, Seattle, Washington, United States of America

[6]Wits Reproductive Health and HIV Institute, Department of Internal Medicine, School of Clinical Medicine, Faculty of Health Sciences, University of the Witwatersrand, Johannesburg, South Africa

*Author for correspondence: Sydney Rosen, Boston University School of Public Health, 801 Massachusetts Ave Room 390, Boston, MA 02118 USA, sbrosen@bu.edu, +1 617 414 1273.

Word count: 4,038

For peer review only

Abstract

Introduction: African countries are rapidly adopting guidelines to offer antiretroviral therapy (ART) to all HIV-infected individuals, regardless of CD4 count. For this policy of “treat all” to succeed, millions of new patients must be initiated on ART as efficiently as possible. Studies have documented high losses of treatment-eligible patients from care before they receive their first dose of antiretrovirals (ARVs), due in part to a cumbersome, resource-intensive process for treatment initiation, requiring multiple clinic visits over a several-week period.

Methods and analysis: The SLATE (Simplified Algorithm for Treatment Eligibility) study is an individually randomized evaluation of a simplified clinical algorithm for clinicians to reliably determine a patient’s eligibility for immediate ART initiation without waiting for laboratory results or additional clinic visits. SLATE will enrol and randomize (1:1) 960 adult, HIV-positive patients who present for HIV testing or care and are not yet on ART in South Africa and Kenya. Patients randomized to the standard arm will receive routine, standard of care ART initiation from clinic staff. Patients randomized to the intervention arm will be administered a symptom report, medical history, brief physical exam, and readiness assessment. Patients who have positive (satisfactory) results for all four components of SLATE will be dispensed ARVs immediately, at the same clinic visit. Patients who have any negative results will be referred for further clinical investigation, counseling, tests, or other services prior to being dispensed ARVs. After the initial visit, follow up will be by passive medical record review. The primary outcomes will be ART initiation ≤28 days and retention in care 8 months after study enrollment.

Ethics: Ethics approval has been provided by the Boston University Institutional Review Board, the University of the Witwatersrand Human Research Ethics Committee (Medical), and the KEMRI Scientific and Ethics Review Unit.

Trial registration: Clinicaltrials.gov NCT02891135, registered September 1, 2016

Keywords: Antiretroviral therapy, Treatment initiation, Africa, South Africa, Kenya, Protocol, Randomized trial

For peer review only

Strengths and limitations of this study

- Evaluates a practical, simple algorithm for accelerating initiation of HIV treatment, a point at which many patients are currently lost from care
- Will contribute to achieving global targets for scale-up of treatment for HIV
- Individually randomized trial conducted in six study sites in two African countries, making the results generalizable to the region
- Comparison arm is standard care, which could change over the course of the study

For peer review only

For peer review only - http://bmjopen.bmj.com/site/about/guidelines.xhtml

INTRODUCTION

In its 2015 revision of the global guidelines for HIV care and treatment, the World Health Organization (WHO) called for initiating lifelong antiretroviral treatment (ART) for all patients testing positive for HIV, rather than waiting for a patient's CD4 count to fall below 500 cells/mm^3, as in the previous guidelines.[1] Many African countries have adopted this recommendation and are rapidly developing new instructions and procedures for its implementation.

In its recommendation, which is referred to as "treat all" or "test and treat", the WHO cited "increases in ART uptake and linkage to care, reduction in the time between HIV diagnosis and ART initiation regardless of baseline CD4 cell count and an increase in the median CD4 value at ART initiation" as some of the main benefits to be gained from the guideline change, a conclusion recently confirmed in a modeling analysis.[2] For countries to benefit from treat all, therefore, millions of new patients must be initiated on ART as efficiently as possible, while ensuring that patient autonomy, welfare, and commitment to lifelong ART are not jeopardized by the initiation process. "Treat all," by removing the requirement of a pre-initiation CD4 count, promises to simplify and accelerate treatment initiation, if efficient procedures can be developed and implemented. Studies from throughout sub-Saharan Africa have documented high losses of treatment-eligible patients from care after they test positive for HIV but before they receive their first dose of antiretrovirals (ARVs), due to a wide range of facility- and patient-level barriers to initiation.[3][4] Multiple preparatory visits, long waiting times, stock outs of supplies, staff absences, and poor communication between staff and patients all deter treatment initiation.[5–8]

We previously proposed a simplified clinical algorithm for allowing nurses and other clinicians to reliably determine a patient's eligibility for immediate ART initiation without waiting for laboratory results or additional clinic visits.[9] The Simplified Algorithm for Treatment Eligibility (SLATE) includes a symptom report, medical history, brief physical exam, and readiness assessment; patients who "screen in" on all four screens can be dispensed ARVs on the spot, while those who do not are referred for further assessment, care, or support. An international technical consultation in 2015 regarded evaluating this algorithm as the highest priority question on

For peer review only - http://bmjopen.bmj.com/site/about/guidelines.xhtml
For peer review only

the operational research agenda it developed[9].

In this paper, we describe the protocol for a pragmatic, individually randomized evaluation of the effectiveness of the new algorithm in increasing ART uptake and retention in care in South Africa and Kenya. The SLATE study will evaluate whether patients who are administered the SLATE screens and either initiated on ART immediately or referred for further care have higher uptake of ART at 28 days and better retention in care at 6 months than do patients who receive standard ART initiation.

The purpose of the SLATE algorithm is to allow clinics to initiate ART in a way that minimizes the time required for both patients and staff and reduces loss to follow up prior to treatment initiation for the majority of patients who require no additional clinical care or psychosocial support before they can start. For those who would benefit from additional care or support—whether an additional counseling session, a TB test, or a more comprehensive physical examination—the algorithm is intended to identify and refer such patients for the additional care required, without slowing down the process for those who do not.

METHODS AND ANALYSIS

SLATE will be an individually randomized, non-blinded, pragmatic evaluation of the effect of the SLATE algorithm on ART initiation and retention in care, conducted at three public-sector clinics each in Kenya and South Africa.

Intervention: The SLATE algorithm and same-visit ART initiation

The intervention to be evaluated in this study is a clinical algorithm that streamlines and structures the information required from patients before ARVs are dispensed for the first time. The algorithm consists of four "screens": symptom report, medical history, brief physical examination, and readiness assessment. Patients who "screen in"—have satisfactory responses to all four screens—can then be dispensed ARV medications on the spot, without any further steps or delays. Patients who screen out—have at least one unsatisfactory response on a

For peer review only

For peer review only - http://bmjopen.bmj.com/site/about/guidelines.xhtml

screen—are referred for further services, such as a laboratory test, more intensive physical examination, or counseling, before ARVs are dispensed.

Table 1 and Figure 1 describe the SLATE screens in detail, and the questions from the SLATE screening instruments to be used in the study are available in Supplementary File 1. Most of the information collected by the screens is consistent with current practice in study countries. The fourth screen, which aims to assess patient "readiness" to start ART, addresses an issue for which there is little standardization among countries or clinics. While emotional and psychological readiness to start ART is often regarded as important to achieving good medication adherence after initiation, a published review of readiness instruments for HIV initiation found that none was notably successful in predicting patient readiness for ART, as indicated by adherence once on treatment.[10] Primarily to ensure that patients have an opportunity to express any reservations they may have about starting ART and ask questions, we developed a three-question readiness assessment instrument.

Table 1. SLATE algorithm screens

Screen	Overall purpose of screen	Reasons for screening out	Justification	If yes, anticipated next step
Symptom report	Identify self-reported conditions that require additional investigation	Current cough, fever, night sweats, or recent weight loss	These symptoms comprise the WHO-recommended tuberculosis symptom screen[11]	Referral for TB test
		Persistent headache for > 2 days	Symptom of cryptococcal meningitis[12,13]	Referral for CrAg screening
		Other self-reported symptoms	Other symptoms could indicate the need for further clinical investigation	Referral for additional clinical consultation
Medical history	Through self-report identify individuals on concurrent medications or who may struggle with adherence	On ART previously	Patients who have been on ART in the past may require additional adherence counseling	Referral for additional counseling session
		Started TB treatment within the past two weeks	Guidelines recommend a two-week delay in ART initiation for patients starting TB treatment	Appointment for ART initiation immediately after the two-week window has passed
		Concurrent medications for epilepsy or current warfarin	These medications can interact with ARVs	Referral for additional clinical or pharmacy consultation
		Current substance abuse	Use of recreational drugs or over-use of alcohol can create challenges for chronic medication adherence	Referral for additional counseling session
Physical examination	Record weight, temperature and blood pressure and identify any observable conditions that require additional investigation	Any conditions that call for further investigation prior to ART initiation	Patient may identify previously unreported symptoms or clinician may observe conditions that indicate a need for further clinical investigation before starting ART	Referral for additional clinical consultation

For peer review only - http://bmjopen.bmj.com/site/about/guidelines.xhtml

Screen	Overall purpose of screen	Reasons for screening out	Justification	If yes, anticipated next step
Readiness assessment	Confirm that the patient feels ready to start ART today	Responses that indicate reluctance, hesitation, or concerns in starting and adhering to treatment	Creates a structured opportunity for clinician and patient to discuss any concerns that the patient has not yet raised	Referral for additional counseling and follow-up support as indicated

Figure 1. Simplified algorithm for treatment eligibility (SLATE)

Outcomes, randomization, and sample size

The study has two primary outcomes, both based on the outcomes proposed for operational research on ART initiation in a previous publication[9]. For primary outcome 1, we will estimate the proportion of patients in each arm (baseline and intervention) who initiate ART within 28 days of study enrollment. Previous studies have found that 28 days is a sufficient time interval for a majority of patients found eligible for ART to complete the steps required to start treatment under routine care.[14,15] A patient who has not initiated within 28 days will be regarded as failing to achieve this primary outcome.

For primary outcome 2, we will estimate the proportion of patients in each arm who initiate ART within 28 days (i.e. achieve primary outcome 1) and are alive, in care, and retained on ART eight months after study enrollment. Eight months was selected to allow up to 1 month (28 days) to initiate ART, six months of follow up after treatment initiation, and up to 1 month to return for the 6-month routine clinic visit. Although viral suppression is preferable to retention in care as a measure of ART success, we are not confident that all the study sites will consistently perform routine viral load tests at 6 months, and viral suppression will be considered as a secondary outcome. To allow for the irregularity of clinic visits, we will allow any clinic visit between 5 and 7 months after treatment initiation (or between 6 and 8 months after study enrollment, taking into account the month allowed for treatment initiation) to represent the 6-month visit.

Secondary outcomes are described in Table 2.

Table 2. Secondary outcomes

For peer review only - http://bmjopen.bmj.com/site/about/guidelines.xhtml

Secondary outcome	Justification and/or further description	Data analysis
**ART outcomes**		
ART initiation within 14 days of study enrollment	National guidelines in both study countries[16,17] recommend initiation ≤ 14 days	Intention to treat analysis; comparison of proportions between groups presented as a risk difference and 95% confidence intervals
Time to initiation, in days	One goal of SLATE algorithm is to accelerate initiation; time to initiation captures any effect on this.	Intention to treat analysis; comparison of time to initiation presented as survival curves with log rank test
Viral suppression by 8 months after study enrollment	Allows ≤ 1 month (28 days) to initiate ART, 6 months of follow up after treatment initiation, and ≤ 1 month to return for the 6-month routine clinic visit	Intention to treat analysis; comparison of proportions between groups presented as a risk difference and 95% confidence intervals
Retention in care 14 months after study enrollment	Allows ≤ 1 month (28 days) to initiate ART, 12 months of follow up after treatment initiation, and ≤ 1 month to return for the 12-month routine clinic visit; any visit 12-14 months after study enrollment will represent the 12-month visit	Intention to treat analysis; comparison of proportions between groups presented as a risk difference and 95% confidence intervals
Retention in care at 16 months after study enrollment	Allows ≤ 1 month (28 days) to initiate ART, 12 months of follow up after treatment initiation, and ≤ 3 months to return for the 12-month routine clinic visit, to allow comparability to other studies of 12 month retention in care, which often define loss to follow-up as 90 days late for the last scheduled visit.	Intention to treat analysis; comparison of proportions between groups presented as a risk difference and 95% confidence intervals
**SLATE evaluation**		
Proportions of study patients who screen in and screen out for immediate ART initiation using SLATE algorithm criteria	Will provide guidance on proportions of patients who could be initiated under SLATE if adopted as routine care	Intention to treat analysis; comparison of proportions between groups presented as a risk difference and 95% confidence intervals
Reasons for ineligibility	Will provide guidance on types of referral services required from clinics	Descriptive analysis of proportions of patients screening out for each possible reason indicated on SLATE screens
Patient preferences on the speed and timing of ART initiation	Baseline questionnaire data	Descriptive analysis of medians and IQRs for continuous outcomes and proportions and corresponding 95% confidence intervals for categorical outcomes
**Health system outcomes**		
Costs to patients of ART initiation under standard and intervention procedures	SLATE is hypothesized to reduce the number of clinic visits required for ART initiation and thus costs to patients	Sum of clinic visit costs and time spent from enrollment visit to visit at which ARVs are dispensed, calculated from questionnaire responses.
Costs to providers of ART initiation under standard and intervention procedures and cost-effectiveness of intervention	SLATE is hypothesized to reduce the number of clinic visits required for ART initiation and thus costs to providers	Estimate of provider costs using previously described[18] bottom-up costing methods, with resource utilization extracted from medical records and CRFs and unit costs obtained from study sites. The average cost to the provider per patient achieving each primary outcome will be compared between intervention and standard initiation groups to provide an estimate of the cost-effectiveness of the two strategies. Costs will be reported as means (standard deviations) and medians (IQRs) in local currencies and USD dollars.

Study patients will be randomized 1:1, using block randomization in blocks of six, to the intervention arm or

For peer review only - http://bmjopen.bmj.com/site/about/guidelines.xhtml

standard care arm. Randomization will be done at the country level, with an expected sample size of 160 subjects per site but with the possibility of allocating more or fewer slots to individual sites, while maintaining randomization rules, if enrollment rates differ. Blinding is not possible in a pragmatic evaluation such as SLATE, as each arm will follow very different procedures post-randomization.

The target sample size for the study is 480 patients per country, or 960 in total. Using results of the RapIT randomized trial we recently conducted in South Africa,[14] we estimated that 65% of treatment-eligible patients will be initiated on antiretroviral therapy and retained on ART in the standard arm, and we considered an increase to 80% to be programmatically important. Using an α of 0.05, power of 90%, 1:1 randomization, and an uncorrected Fisher's exact test, this requires a minimum sample size of 197 patients per group, which we will increase to a maximum of 240 to ensure sufficient power if patients withdraw or are found ineligible after consent.

Study sites and ethics review

South Africa and Kenya, where the study will be conducted, differ widely in income and HIV prevalence, and thus will allow us to determine the effectiveness of the intervention in varying settings. In each country, patients will be enrolled at three public-sector clinics that have infrastructure and staff that are typical of the facilities that treat the vast majority of HIV patients. The only restriction we applied in selecting sites was that they have relatively high patient volumes, to speed up study enrollment. The sites are described in Table 3.

Table 3. Study sites

Country, implementer, and registry	Province/district or county	Site name	Patient population served
South Africa: Health Economics and Epidemiology Research Office (HE^2RO), University of the Witwatersrand, Johannesburg. South African National Health Research Database GP_2016RP42_318.	Gauteng Province, Johannesburg Metro	OR Tambo Primary Health Clinic	Urban informal settlement (Diepsloot)
	Gauteng Province, Johannesburg Metro	Alexandra Community Health Centre	Urban informal settlement (Alexandra)
	Gauteng Province, Ekhurhuleni Metro	Jabulani Dumani Community Health Centre	Urban informal settlement (Vosloorus)
Kenya: Kenya Medical Research Institute/Walter Reed Projects (KEMRI/WRP), Kericho. Pan African Clinical Trial Registry PACTR201609001783150.	Kericho County	Kericho County Referral Hospital	Kericho town and surrounding rural areas
	Kapsabet County	Kapsabet County Referral Hospital	Kabsabet town and surrounding rural areas
	Kombewa County	Kombewa County Hospital	Rural areas northwest of Kisumu

For peer review only - http://bmjopen.bmj.com/site/about/guidelines.xhtml

Study implementation in each country will be led by a local research organization subcontracted to the primary study implementer, Boston University (Table 3). At each site, the study will employ a study nurse (South Africa) or clinical officer (Kenya) in the same professional cadre that initiates patients onto ART under standard care. Each site will also have one or two study assistants, who will be responsible for screening potential subjects, obtaining informed consent, administering the baseline questionnaire, and performing other non-clinical tasks. The study will have two dedicated rooms or private spaces at the sites, one for the nurse/clinical officer and one for the study assistant. Where necessary, we will renovate existing space or provide a separate, stand-alone space, such as a trailer, for study activities.

The study protocol, which is available as Supplementary File 2, has been approved by the Boston University Institutional Review Board and the University of the Witwatersrand Human Research Ethics Committee (Medical) and is under review by the KEMRI Scientific and Ethics Review Unit and the Walter Reed Army Institute of Research Institutional Review Board. It is registered with Clinicaltrials.gov as NCT02891135.

Screening and enrollment

At each site, we will recruit 160 adult patients (≥18 years) who have tested positive for HIV, either at the current clinic visit or previously, and have not yet initiated ART or begun the clinic's routine ART initiation steps, such as adherence education sessions. Pregnant women will be excluded, as procedures for initiating and managing pregnant women on ART differ from those for non-pregnant adults. Patients who intend to receive further HIV care at a different clinic, rather than the study site, will also be excluded, as will those who are determined by study staff to be physically, mentally, or emotionally unable to participate.

Site staff will refer patients with HIV to the study assistant for study screening and consent. Patients will be screened consecutively in the order in which they are referred by the site staff, based on the availability of the study assistant. All patients who are found eligible for the study through the screening process will be asked for written informed consent and enrolled in the study. Following consent, female patients will be asked to complete a

For peer review only - http://bmjopen.bmj.com/site/about/guidelines.xhtml

pregnancy test, and any who are found to be pregnant will be withdrawn from the study and escorted to the site's antenatal clinic to enroll in antenatal care and prevention of mother-to-child transmission (PMTCT) care.

One of the goals of implementation science studies is to generate evidence in a time frame that is programmatically relevant. To accelerate reaching the country enrollment targets, unequal enrollment by site will be allowed, to take advantage of sites that have higher patient volumes. Enrollment in SLATE is expected to be completed within a 6-8 month period in 2017. The first primary outcome can then be estimated just 28 days after the last patient has been enrolled, while the second primary outcome will require 8 months of follow up. We thus expect most study results to be available by the end of 2018.

Procedures

Figure 2. Study flow diagram

Study procedures are illustrated in Figure 2. Prior to randomization, patients who provide written informed consent to study participation will be administered a baseline questionnaire by the study assistant, with sections on demographic and socioeconomic status, costs incurred per clinic visit, and preferences for the timing and speed of ART initiation. Randomization assignments, stored in sealed envelopes, will be opened and conveyed to participants by the study assistant, after completing the questionnaire. After randomization, patients assigned to the standard arm will have no further study activities. They will receive a payment equivalent in value to USD 5-15 to thank them for participating and compensate them for their time. Payment will be in the form of a shopping voucher that can be used at nearby grocery/general goods stores in South Africa and cash in Kenya. The study assistant will then escort standard arm patients back to the clinic reception desk to resume their place in the patient queue and complete a standard clinic visit, as they would have done in the absence of the study, and will follow the clinic's regular schedule of procedures and visits for ART initiation. They will have no further interaction with study staff after this point.

For peer review only

For peer review only - http://bmjopen.bmj.com/site/about/guidelines.xhtml

Patients assigned to the intervention arm will be introduced to the study nurse/clinical officer, who will administer the four SLATE screens. For the study, all four screens will be completed for all patients assigned to the intervention arm, so that we can obtain a complete data set for all patients. If SLATE were used in routine care, we expect that the screens would be administered sequentially, so that a negative response to the first screen (for example, report of a symptom of TB) would allow a decision to refer the patient for additional care to be made without having to complete the second screen, and so on. Patients would progress to the next screen only after being found positive on the previous one, where "positive" refers to satisfactory responses to all items on the screen, while "negative" indicates a response that requires referral. We expect that for the vast majority of patients, each screen will take less than five minutes to administer. We would anticipate that in routine practice the algorithm will take 15-20 minutes for most patients.

Following administration of the four screens, intervention arm patients will have a blood draw to allow the study to collect baseline CD4 counts and for any pre-initiation laboratory tests called for under standard care. Although baseline CD4 counts are no longer used in either study country to establish ART eligibility, they remain a strong indicator of early outcomes on ART, and are therefore an important variable for the study analysis. Blood samples will be sent for processing at the same laboratories used by the study sites.

The results of the four screens will indicate whether a patient has either "screened in" or "screened out" of the SLATE algorithm. Findings in any of the four screens that suggest that further clinical investigation, counseling, laboratory tests, or other services are advisable prior to dispensing ARVs will cause a patient to screen out. Patients who screen out will be referred for the suggested follow-up services and escorted to the appropriate clinic location for immediate follow up at the same visit where possible. If the recommended follow-up service is not available immediately, a follow-up appointment will be made for the patient.

It is important to note that screening out of the SLATE algorithm will not necessarily preclude same-day ART initiation, either in the study or if the algorithm were used in routine care. Many patients who screen out will receive the additional service at the same clinic visit (e.g. an additional meeting with a counselor) and can still be

For peer review only - http://bmjopen.bmj.com/site/about/guidelines.xhtml

prescribed ARVs before completing the visit. In fact, we expect that many patients who screen out of SLATE could indeed start ART on the same day, if the clinic has the capacity to provide the follow-up service that is needed and there is enough time left in the day for the patient to be served. SLATE is not intended to delay ART initiation for those who screen out, but rather to accelerate the process for those who screen in.

For patients who "screen in"—have satisfactory responses to all four SLATE screens and do not require any additional services—the study clinician will then have a brief conversation with the patient to confirm that the patient remains ready to start ART, understands what happens next in the study, and has no further questions or concerns. The clinician will then write a prescription for an initial supply of medications and either dispense the medications directly from the clinician's room or escort the patient to the clinic pharmacy for immediate dispensing, as per the usual practice at each clinic. Patients in the intervention arm will then receive the same token of appreciation (shopping voucher or cash) as was provided earlier to patients in the standard arm.

Data collection and management

For the primary analysis, the two main sources of study data will be case report forms (CRF) and patients' routine medical records. The CRF includes responses to the baseline questionnaire and all four SLATE screens. It also has sections for confirming a participant's randomization assignment, CD4 count, medication dispensing, next appointment date, and/or referral for further care, as needed. It concludes with a follow-up form for recording study outcomes manually from paper files, in case these are not available electronically at every site.

The SLATE CRF will be programmed in REDCap Mobile (https://www.project-redcap.org/software/mobile-app/), housed at Boston University. REDCap (Research Electronic Data Capture) is a secure, web-based application designed to support data capture for research studies.[19] Data capture forms will be built in REDCap's web application and then loaded securely onto tablet computers for direct entry by study staff on site. Using REDCap Mobile, data can be entered off line, then uploaded to the REDCap server as soon as an internet connection is available. The program will ensure a consistent study identification number for each participant, maintain data

For peer review only - http://bmjopen.bmj.com/site/about/guidelines.xhtml

security, and allow data quality monitoring by the study data management team in near-real time. Tablets will be accessible only to the study staff at the specified site, and data that are uploaded to the server will automatically be erased from the tablets, to reduce any chance of loss of confidentiality.

With the exception of the follow-up form, CRFs will be completed at the end of a patient's enrollment visit. All data for the follow-up period, until all primary and secondary outcomes are reached, will come from routinely collected medical records. All the study sites maintain an electronic medical record (EMR) database that we will access for most follow up data. In South Africa, sites use Tier.Net, the national HIV monitoring system.[20] In Kenya, sites use either IQCare (https://fgiqcare.codeplex.com/) or the Kenya version of Open MRS (https://wiki.openmrs.org/display/ke/KenyaEMR). Because the electronic records are not always complete or up to date, for example if a clinic is short-staffed and a data entry backlog develops, the CRF follow-up form will be completed where needed.

To allow linking of data between the CRF and medical records, a separate linking form, also in REDCap, will be completed for each patient immediately after consent. The linking form will contain the patient's name, date of birth, clinic file number, and national identification number. It will be stored separately from all other data collection forms, and used solely to select the right medical records (electronic and/or paper) for each study participant.

In addition to the two main sources of data described above, data from screening forms, which will be entered into a separate REDCap database, will be used to compare the enrolled sample with all patients screened for the study, to determine if the study sample is representative of the screened population and summarize reasons for screening out of the study. We will also collect aggregate, facility-level information on patient volume, resource utilization, and unit costs to answer our secondary research questions.

CRF data and other information collected in REDCap (screening forms, linking forms) will be uploaded to the REDCap server on a daily basis, using either the site's own internet access or a cellular network uplink from the

For peer review only - http://bmjopen.bmj.com/site/about/guidelines.xhtml

tablets. Following an initial period of daily quality review, a study manager will monitor data quality and completeness on a weekly basis. Queries about the data will be sent to study coordinators for follow up and correction by site level staff, as needed.

Data generated by the study (case report forms) will be made available in de-identified format following closure of the protocol in a publicly available repository, to be identified in papers published from the study. Data obtained from the study sites (routinely generated medical record data) will not be owned by the study and cannot be made publicly available by the authors.

Data analysis

The analytic approach will be by intention-to-treat: subjects will be analyzed according to the intervention they were supposed to receive, whether or not they adhered to the defined intervention. Outcomes for patients randomized to the intervention arm who screen out of immediate ART initiation by the SLATE algorithm will count towards the intervention arm. Analyses will be pooled across countries, but we will also stratify the analysis of primary outcomes by country.

The analysis will begin with a simple comparison of the two treatment groups with respect to baseline predictors of outcomes to detect any imbalances. We will then compare the proportion of patients achieving each dichotomous outcome by group and will calculate crude risk ratios and crude risk difference and their corresponding 95% confidence intervals. We will look for effect modification by important predictors of the outcome. The primary modifier is expected to be country, as standard of care for ART initiation differs between the two study countries. We will also look for differences in effects stratified by age, sex, baseline body mass index, and CD4 count, which are predictors of retention in care, and by any other important demographic, socioeconomic, and clinical predictors of outcomes identified, using baseline data including questionnaire responses. Our analysis for effect modification will use a simple stratification of the primary analysis by the potential modifier and reporting crude risk differences and risk ratios and their corresponding 95% confidence

For peer review only - http://bmjopen.bmj.com/site/about/guidelines.xhtml

intervals.

Analytic methods for secondary outcomes are described in Table 2.

Limitations

We anticipate that the SLATE study will have three main limitations. First, the number of study countries will be small, which could reduce generalizability if patient populations differ in characteristics that affect study outcomes. Second, in order to reach the target sample size in the desired timeframe, there will be heterogeneity in the population enrolled, with some patients who have spent years in pre-ART monitoring and others who were diagnosed with HIV on the day of study enrollment. And third, as in most operational research studies, we will have little control over what happens in our baseline (non-intervention) arm. Standard of care continues to evolve rapidly in Africa. Guideline revisions are frequent, and clinicians at the study sites may learn from the intervention and make adjustments to routine care before the study is completed. Any changes that occur will be reported by study staff and taken into account in data analysis.

DISCUSSION

Improving the efficiency of HIV service delivery—which encompasses both improving outcomes and reducing costs—is a high priority of national governments and international agencies tasked with implementing and paying for universal access to antiretroviral therapy. Patients who have already volunteered for HIV testing but not yet started ART are a promising target for intervention, as they have already identified themselves and, by coming forward for testing, provided a time and place to intervene. Streamlining procedures for HIV initiation thus offers a relatively easy way to increase efficiency. The resources freed by such improvements can, in turn, be used to expand access to and quality of healthcare provision overall.

For peer review only - http://bmjopen.bmj.com/site/about/guidelines.xhtml

The SLATE algorithm that we will evaluate in this study has the potential to reduce the time and resources that both providers and patients must invest in ART initiation, while also diminishing the likelihood that patients will get lost from care between diagnosis and treatment initiation. If it can do so without jeopardizing outcomes after starting ART—such as retention in care during the first year, as will be monitored by the study—then SLATE will offer national HIV programs and providers a new and more efficient approach to an important component of HIV care. As other researchers have argued, rigorous implementation studies of feasible and pragmatic approaches to reducing losses to care are essential to achieving global targets.[21] The SLATE study aims to generate evidence for one new such approach.

Figure 1 legend

Figure 1 adapted from Rosen S, Fox MP, Larson B, Sow PS, Ehrenkranz PD, Venter F, Manabe Y, Kaplan J, for the Models for Accelerating Treatment Initiation (MATI) Technical Consultation. Accelerating the uptake and timing of antiretroviral therapy initiation in sub-Saharan Africa: an operations research agenda. *PLoS Med* 2016; 13: e1002106. DOI:10.1371/journal.pmed.1002106 (CC BY 4.0).

Competing interests:

PDE is an employee of the Bill & Melinda Gates Foundation, which is funding this work. WDFV sits on antiretroviral initiation guideline committees, both local and international. WDFV has accepted speaking honoraria from multiple manufacturers of antiretrovirals and is on several of their advisory boards. The remaining authors declare that they have no competing interests.

Funding:

Funding for the work presented here was provided by the Bill & Melinda Gates Foundation under the terms of OPP1136158 to Boston University. The funders participated in the Technical Consultation reported here and are co-authors of the manuscript. The funders had no separate role, beyond that of other participants and authors, in study design, data collection and analysis, decision to publish, or preparation of the manuscript.

Authors' contributions:

Conceived of and designed the work: SR, MPF, BAL, PDE, WDFV. Contributed to designing the work: ATB, MM, IT, MB. Wrote the first draft of the manuscript: SR. Reviewed and revised the manuscript: all authors. Approve the manuscript's results and conclusions: all authors. All authors have read, and confirm that they meet, ICMJE criteria for authorship.

References

1. World Health Organization. Guideline on when to start antiretroviral therapy and on pre-exposure prophylaxis for HIV. Geneva: World Health Organization, 2015.
2. Barnabas RV, Bendavid E, Bershteyn A, Boulle A, Eaton JW, Ford N, et al. HIV mortality by care cas- cade stage and implications for universal ART eligibility. Conference on Retroviruses and Opportunistic Infections 2016;

For peer review only - http://bmjopen.bmj.com/site/about/guidelines.xhtml

22–25 Feb 2016; Boston. Available: http://www.croiconference. org/sessions/hiv-mortality-care-cascade-stage-and-implications-universal-art-eligibility.
3. Lahuerta M, Ue F, Hoffman S, Elul B, Kulkarni SG, Wu Y, et al. The problem of late ART initiation in Sub-Saharan Africa: a transient aspect of scale-up or a long-term phenomenon? J. Health Care Poor Underserved 2013;24:359–83.
4. Iwuji CC, Orne-Gliemann J, Larmarange J, Okesola N, Tanser F, Thiebaut R, et al. Uptake of home-based HIV testing, linkage to care, and community attitudes about ART in rural KwaZulu-Natal, South Africa: Descriptive results from the first phase of the ANRS 12249 TasP cluster-randomised trial. PLOS Med 2016;13:e1002107.
5. Govindasamy D, Ford N, Kranzer K. Risk factors, barriers and facilitators for linkage to ART care in sub-Saharan Africa: a systematic review. AIDS 2012;26:2059–67.
6. Micek M a, Gimbel-Sherr K, Baptista AJ, Matediana E, Montoya P, Pfeiffer J, et al. Loss to follow-up of adults in public HIV care systems in central Mozambique: identifying obstacles to treatment. J Acquir Immune Defic Syndr 2009;52:397–405.
7. Scott V, Zweigenthal V, Jennings K. Between HIV diagnosis and initiation of antiretroviral therapy: assessing the effectiveness of care for people living with HIV in the public primary care service in Cape Town, South Africa. Trop Med Int Heal 2011;16:1384–91.
8. Siedner MJ, Lankowski A, Haberer JE, Kembabazi A, Emenyonu N, Tsai AC, et al. Rethinking the "'pre'" in pre-therapy counseling: no benefit of additional visits prior to therapy on adherence or viremia in Ugandans initiating ARVs. PLoS One 2012;7:e39894.
9. Rosen S, Fox MP, Larson B, Sow PS, Ehrenkranz PD, Venter F, et al. Accelerating the uptake and timing of antiretroviral therapy initiation in sub-Saharan Africa: an operations research agenda. PLoS Med. 2016;13:e1002106.
10. Grimes RM, Grimes DE. Readiness: The state of the science (or the lack thereof). Curr HIV/AIDS Rep 2010;7:245–52.
11. World Health Organization. Guidelines for intensified tuberculosis case-finding and isoniazid preventive therapy for people living with HIV in resource-constrained settings. Geneva: World Health Organization, 2011.
12. Wajanga BM, Kalluvya S, Downs JA, Johnson WD, Fitzgerald DW, Peck RN. Universal screening of Tanzanian HIV-infected adult inpatients with the serum cryptococcal antigen to improve diagnosis and reduce mortality: An operational study. J Int AIDS Soc 2011;14:48.
13. Aoussi EF, Ehui E, Demb JP, Kolia-Diafouka P, Elloh NF, Ouattara SI, et al. Cryptoccocal meningitis and HIV in the era of HAART in Cote d'Ivoire. Med Mal Infect 2012;42:349–54.
14. Rosen S, Maskew M, Fox MP, Nyoni D, Mongwenyana C, Malete G, et al. Initiating antiretroviral therapy for HIV at a patient's first clinic visit: the RapIT randomized controlled trial. PLoS Med. 2016;13:e1002015.
15. Amanyire G, Semitala FC, Namusobya J, Katuramu R, Kampiire L, Wallenta J, et al. Effects of a multicomponent intervention to streamline initiation of antiretroviral therapy in Africa: a stepped-wedge cluster-randomised trial. Lancet HIV 2016; 3:e539-e548.
16. Ministry of Health National AIDS & STI Control Programme. Guidelines on use of antiretroviral drugs for treating and preventing HIVinfection in Kenya 2016. Nairobi; Ministry of Health, 2016.
17. Pillay Y. Implementation of the universal test and treat strategy for HIV positive patients and differentiated care for stable patients. Pretoria: Department of Health, 2016.
18. Long L, Brennan A, Fox MP, Ndibongo B, Jaffray IA, Sanne I, et al. Treatment outcomes and cost-effectiveness of shifting management of stable ART patients to nurses in South Africa: an observational cohort. PLoS Med 2011;8:e1001055.
19. Harris PA, Taylor R, Thielke R, Payne J, Gonzalez N, Conde JG. Research electronic data capture (REDCap)—A metadata-driven methodology and workflow process for providing translational research informatics support. J Biomed Inform 2009;42:377–81.
20. Osler M, Hilderbrand K, Hennessey C, Arendse J, Goemaere E, Ford N, et al. A three-tier framework for monitoring antiretroviral therapy in high HIV burden settings. J Int AIDS Soc 2014;17: 18908
21. Labhardt ND, Ringera I, Lejone TI, Masethothi P, Thaanyane T, Kamele M, et al. Same day ART initiation versus clinic-based pre-ART assessment and counselling for individuals newly tested HIV-positive during community-based HIV testing in rural Lesotho – a randomized controlled trial (CASCADE trial). BMC Public Health 2016;16:329.

For peer review only

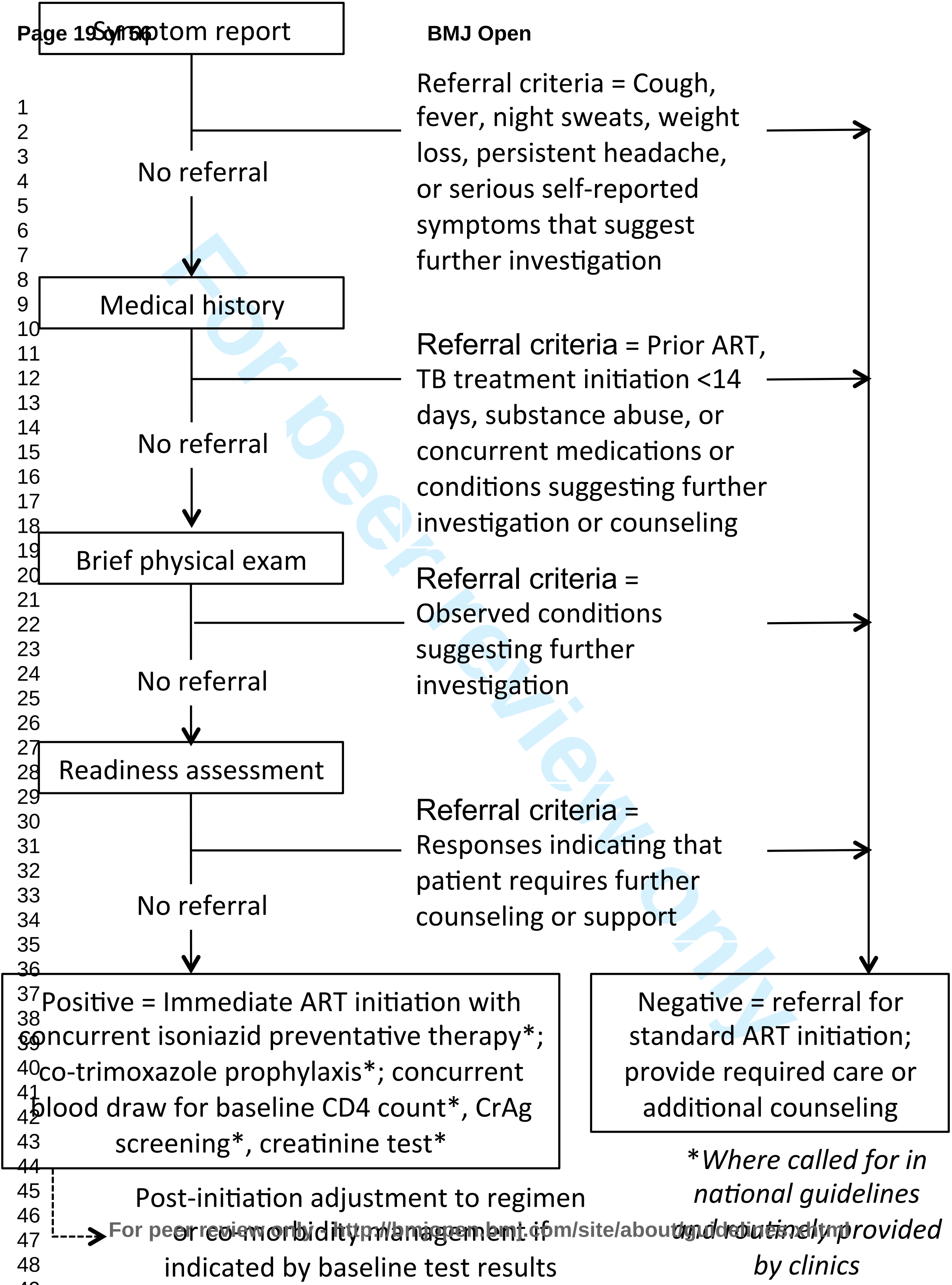

For peer review only

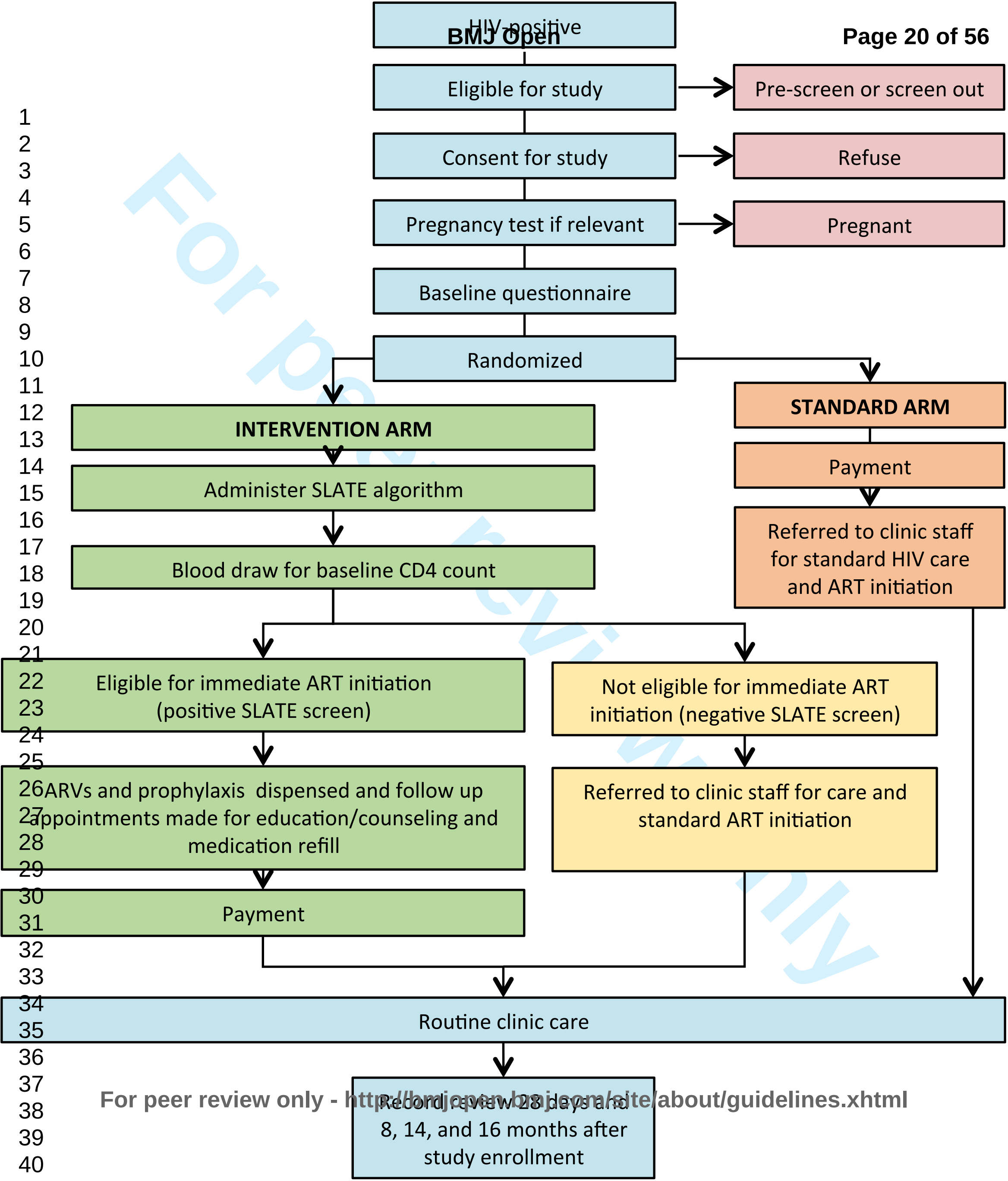

For peer review only

SIMPLIFIED ALGORITHM FOR TREATMENT ELIGIBILITY (SLATE): SCREENS

Symptom report

1. Does patient have a cough? Yes/No
2. Does patient have a fever? Yes/No
3. Does patient report having night sweats? Yes/No
4. Does patient report losing weight? Yes/No
5. Does patient have a continuous headache that hasn't gone away for at least two days? (If headache is persistent or continuous, answer "yes." If occasional or temporary, answer "no.") Yes/No
6. Does patient report any other symptoms of illness? Yes/No

   If yes, what other symptoms? (Specify)

   If yes, do any of the other symptoms indicate that further consultation is needed before starting ART? Yes/No
7. On the basis of symptoms, should this patient be referred for further care before starting ART? Yes/No

Medical history

1. Have you been on ART before? Yes/No

   If yes, when did you start taking ART before? (Record)

   If yes, when did you stop taking ART before? (Record)
2. Are you currently on TB treatment? Yes/No

   If yes, how many weeks ago did you start taking TB treatment? (Record)

   If patient started taking TB treatment 1 or 2 weeks ago, how many days ago did she or he start TB treatment? (Record)
3. Have you been told that you have other diseases or health problems besides HIV? Yes/No

   If yes, what other diseases or conditions do you have? (Specify)

   If yes, do any of the diseases or conditions suggest further consultation is needed? Yes/No
4. Are you currently taking any anti-epilepsy medication? Yes/No
5. Are you currently taking warfarin? Yes/No
6. Are you currently taking any other medications? Yes/No

   If yes, what other medications are you currently taking? (Specify)

   If yes, do any of the medications suggest that further consultation is needed? Yes/No
7. Do you currently take any recreational drugs, such as smoking (bhang/ dagga/ weed) or using pills or injectables? Yes/No

For peer review only

NOT FOR USE IN PRACTICE

8. Do you currently drink more alcohol than you think you should? Yes/No
9. On the basis of medical history, should this patient be referred for further care before starting ART? Yes/No

Physical examination

1. Temperature (Record)
2. Systolic blood pressure (Record)
3. Diastolic blood pressure (Record)
4. Weight (kg) (Record)
5. Did the patient report symptoms that should be examined? Yes/No

   If yes, after examination, do any symptoms suggest that further consultation is needed? Yes/No

   If yes, which symptoms should be investigated further? (Specify)
6. Does the patient have other conditions for which further consultation is needed? Yes/No

   If yes, what are the other conditions? (Specify)
8. On the basis of the physical exam, should should this patient be referred for further care before starting ART? Yes/No

Readiness assessment

1. If you should start ART today, how ready are you?
   Not ready
   Thinking about trying, maybe in the next month or so
   Almost ready, maybe this week
   Ready today
2. How will you remember to take your medication every day?
   Mobile phone reminder
   Alarm on a clock or watch
   Ask someone to remind me
   Use a calendar or diary
   Take my tablets at the same time as I do something else every day (like brushing my teeth)
   No reminders, I'll just remember
   Other way (specify)
3. Is there anything that will stop you from taking your tablets every day? Yes/No
4. Did the patient raise any issues or concerns that lead you to think that ART should be delayed?

   If yes, what were these issues or concerns and how did you respond?
5. On the basis of the readiness assessment, should this patient be referred for further care before starting ART? Yes/No

For peer review only

NOT FOR USE IN PRACTICE

SLATE

Research Protocol

Randomized Evaluation of a Simplified Clinical Algorithm for Identifying Patients Eligible for Immediate Initiation of Antiretroviral Therapy for HIV (SIMPLIFIED ALGORITHM FOR TREATMENT ELIGIBILITY —THE SLATE STUDY)

BUMC IRB Protocol Number: H-35634 (United States)

Wits University HREC Protocol Number: 160910 (South Africa)

KEMRI SERU Protocol Number: XXX (Kenya)

WRAIR IRB Protocol Number: XXX (United States)

Federal-Wide Assurance Numbers:

FWA00000301 (Boston University Medical Campus)
FWA00000715 (University of the Witwatersrand)
FWA 00002066 (Kenya Medical Research Unit)
FWA00000015 (Walter Reed Army Institute of Research)

January 13, 2017

Version 1.1

For peer review only

Table of Contents

1. Summary **4**
2. Investigators **4**
3. Background **5**
3a. Context: New guidelines for HIV treatment eligibility 5
3b. The problem: loss to follow up before treatment initiation 6
3c. Proposed solution: a standardized clinical algorithm for determining eligibility for immediate treatment initiation 7
4. Objectives **9**
4a. Primary objectives 9
4b. Secondary objectives 9
5. Study Design **10**
5a. Overview 10
5b. Study sites 11
5c. Study population 12
6. Enrollment and Preparation **13**
6a. Pre-screening 13
6b. Screening 13
6c. Consent 14
6d. Pregnancy test 14
6e. Questionnaire 14
6f. Randomization 14
6g. Payment 14
7. Intervention **15**
7a. Algorithm data collection 15
7b. Blood draw 16
7c. Referral of patients with positive screens 16
7d. Dispensing of ARVs and prophylaxis 16
7e. Completion of direct study interaction 17
8. Medical Record Follow Up **17**
9. Data Collection and Management **18**
9a. Sources of data 18
9b. Pre-screening logbook 18
9c. Screening form 18
9d. Case report form 18
9e. Medical record data 19
9f. Study identification numbers and linking of records 21
9g. Data entry and storage 21
10. Staff Supervision and Training **22**
11. Sample Size **22**
12. Outcomes and Data Analysis **22**

For peer review only - http://bmjopen.bmj.com/site/about/guidelines.xhtml

12a. Outcomes 22
12b. Analytic methods 23
12c. Dissemination of findings 24
13. Human Subjects Considerations........ 25
13a. Risks and protection against risks 25
13b. Benefits 26
13c. Costs and payments to subjects 27
13d. Recruitment 27
13e. Informed consent 27
13f. Protection of confidentiality 28
14. References 28
12. Appendices 29

For peer review only

For peer review only - http://bmjopen.bmj.com/site/about/guidelines.xhtml

1. Summary

In its 2015 revision of the global guidelines for HIV care and treatment, the World Health Organization called for initiating lifelong antiretroviral treatment (ART) for all patients testing positive for HIV, regardless of CD4 cell count. As countries adopt the new recommendation, known as "treat all," millions of additional patients are becoming eligible for ART worldwide. In sub-Saharan Africa, where most of these patients are located, studies continue to document high losses of treatment-eligible patients from care before they receive their first dose of antiretroviral medications (ARVs). Among facility-level reasons for these losses are treatment initiation protocols that require multiple clinic visits and long waiting times before a patient who tests positive for HIV is dispensed an initial supply of medications. Simpler, more efficient, accelerated algorithms for ART initiation will be needed if "treat all" is to realize the benefits expected.

At a technical consultation convened in October 2015, participants proposed a simplified clinical algorithm to screen patients for eligibility for immediate ART initiation at a patient's first clinic visit, without the use of point-of-care laboratory test technologies. The Simplified Algorithm for Treatment Eligibility (SLATE) uses four screens to assess whether a patient is eligible for immediate (same-day) treatment initiation: i) symptom report, ii) medical history, iii) readiness assessment, and iv) brief physical examination. SLATE is a pragmatic, individually randomized evaluation to determine the effectiveness of the algorithm in increasing ART initiation, compared to standard care, among non-pregnant adult patients. A total of approximately 960 HIV-infected adult patients not yet on ART will be enrolled during a routine clinic visit and randomized to receive the intervention or standard care. Patients in the intervention arm will be administered the SLATE screens; those found eligible under the algorithm will be offered immediate treatment initiation, while those who are not eligible will be referred for standard clinic care. Patients in the standard arm will be referred for ART initiation under standard clinic procedures. All care after the initial visit will be by the clinic under standard of care. The study will be conducted at three healthcare facilities (clinics) in each of two countries, South Africa and Kenya. If successful, SLATE will offer a standardized approach to collecting and interpreting a minimum set of patient data that will avoid delaying treatment initiation for the majority of patients who are eligible for immediate ART, while deferring initiation in the minority who should not start immediately.

2. Investigators

Sydney Rosen
Research Professor
Boston University School of Public Health
Boston, MA, USA
Role on study: principal investigator

Matthew Fox
Associate Professor
Boston University School of Public Health
Boston, MA, USA
Role on study: co-investigator, statistical advisor

Bruce Larson
Research Professor
Boston University School of Public Health

For peer review only - http://bmjopen.bmj.com/site/about/guidelines.xhtml

Boston, MA, USA
Role on study: co-investigator, evaluation advisor

Alana Brennan
Research Scientist
Boston University School of Public Health
Boston, MA, USA
Role on study: co-investigator, study manager

Francois Venter
Associate Professor
Department of Medicine
Wits University
Johannesburg, South Africa
Role on study: co-investigator, clinical advisor

Mhairi Maskew
Senior Researcher
Health Economics and Epidemiology Research Office
University of the Witwatersrand
Johannesburg, South Africa
Role on study: South Africa PI, data analyst

Isaac Tsikhutsu
Medical Doctor
Kenya Medical Research Institute/Walter Reed Project HIV Program
Kericho, Kenya
Role on study: Kenya PI

3. Background

3a. Context: New guidelines for HIV treatment eligibility

In its 2015 revision of the global guidelines for HIV care and treatment, the World Health Organization called for initiating lifelong antiretroviral treatment (ART) for all patients testing positive for HIV, regardless of CD4 cell count[1]. This revision followed a series of increases in the global guidelines in the threshold for initiating ART, from a CD4 count of ≤200 cells/mm^3 prior to 2010, to 350 until 2013, to 500 at present. In its recommendation, the WHO cites three anticipated benefits from this approach, which is referred to as "treat all" (alternatively, "test and treat" or "test and start"): reduced morbidity among HIV-infected patients; reduced risk of transmission from HIV-infected individuals to their partners; and "*increases in ART uptake and linkage to care, reduction in the time between HIV diagnosis and ART initiation regardless of baseline CD4 cell count and an increase in the median CD4 value at ART initiation*." A recent analysis concluded that future reductions in HIV-related mortality in sub-Saharan Africa will derive largely from this last benefit, rather than from the first two[2].

Multiple African countries—including South Africa and Kenya, where the proposed study will take place—have already announced their plans to adopt the new guidelines, and it is clear that the trend globally is toward offering ART to all. As this happens, national HIV programs will face the challenge of

For peer review only - http://bmjopen.bmj.com/site/about/guidelines.xhtml

initiating individuals newly diagnosed as HIV-infected on ART as efficiently as possible, while ensuring that retention on ART and patient welfare are not jeopardized by the initiation process.

3b. The problem: loss to follow up before treatment initiation

Studies from throughout sub-Saharan African continue to document high losses of treatment-eligible patients from care before they receive their first dose of ARVs. A systematic review of the literature on pre-ART retention in care found that roughly one third of adult patients in sub-Saharan Africa who had already been determined eligible for ART did not start ART within the studies' follow-up periods[3]. If those diagnosed with HIV but not assessed for treatment eligibility had been added, this proportion would have been far higher. Reasons for this include a wide range of facility- and patient-level barriers to initiation[4]. Among facility-level barriers are clinic inaccessibility and inefficiency and initiation protocols designed for the convenience of providers rather than patients. These barriers manifest themselves as multiple required visits, long waiting times, stock outs of supplies, staff absences, and poor communication between staff and patients. Eliminating the eligibility threshold under a "treat all" policy is not likely to solve these problems, as clinics become even more crowded with newly-eligible patients. Simpler, more efficient, accelerated algorithms for ART initiation will be needed if "treat all" is to realize the benefits expected.

In an earlier clinical trial in South Africa (RapIT, H-31880), we demonstrated that same-day treatment initiation, in which all the steps required for ART initiation are performed in sequence during a single clinic visit, significantly increased the proportion of eligible patients started on treatment (RR 1.36, 95% CI 1.24–1.49) and the proportion virally suppressed six months later (RR 1.26, 95% CI 1.05–1.50).[5] The RapIT trial was premised on the notion that standard of care treatment initiation, which in South Africa required 5 or 6 clinic visits over a 1-2 month period, created a burden on patients that deterred many from completing the process. In addition to delaying the start of treatment, each clinic visit entailed transport costs and time, many hours of waiting time in the clinic, and possibly lost wages, child care, and other costs for the patient[6]. The results of the study indicate that accelerating the initiation process does overcome these barriers; in the intervention arm of the study, 75% of study subjects started treatment on the same day as study enrollment, and nearly all the rest within one month. The study demonstrated that it is possible to initiate nearly 100% of eligible patients, and eliminate loss to follow up at this point in the care cascade.

Although RapIT and other studies have shown that the vast majority of patients presenting for HIV care at clinics in Africa can safely start ART on the same day, without delay, there are still patients for whom a delay is necessary. These reasons include:

- Tuberculosis (TB): To minimize the risk of immune reconstitution inflammatory syndrome (IRIS), two weeks of TB treatment is recommended for patients presenting with active TB before ART is started.
- Cryptococcal meningitis (CM): Patients with low CD4 counts should be screened for cryptococcal antigen (CrAg) and given presumptive treatment for CM before ART is started, again to minimize the risk of IRIS.
- Other clinical conditions: HIV patients present with a range of symptoms and conditions that may lead a clinician to decide to delay ART while further investigations or treatment are completed.
- Patient history: Patients who previously defaulted from ART or have a history predicting non-adherence, such as substance abuse, may require additional support before starting ART.
- Patient readiness: Finally, some patients may feel that they are not ready to start treatment on the same day and choose for themselves to delay initiation.

For peer review only - http://bmjopen.bmj.com/site/about/guidelines.xhtml

The RapIT trial used multiple rapid, point-of-care laboratory test instruments to generate the laboratory data required for ART initiation in South Africa. The generalizability of its findings is thus limited by the need for expensive equipment that will not be feasible to maintain in most primary healthcare clinics in the region. RapIT also excluded many ART-eligible patients on the basis of clinical trial-specific exclusion criteria that would not apply in a routine care setting. What is needed now is a way to assess eligibility for immediate treatment initiation that is broadly applicable to nearly all patients and does not require either point-of-care laboratory instruments or delays for laboratory results to be received, while screening out patients who should not start treatment immediately for clinical or personal reasons.

3c. Proposed solution: a standardized clinical algorithm for determining eligibility for immediate treatment initiation

In October 2015, the investigators convened a technical consultation of experts in the field to discuss models for accelerating treatment initiation[7]. Participants in that meeting proposed a simplified clinical algorithm to screen patients for eligibility for immediate ART initiation without the use of point-of-care technologies. The simplified algorithm, abbreviated “SLATE,” is illustrated in Figure 1.

For peer review only

Figure 1. SLATE algorithm

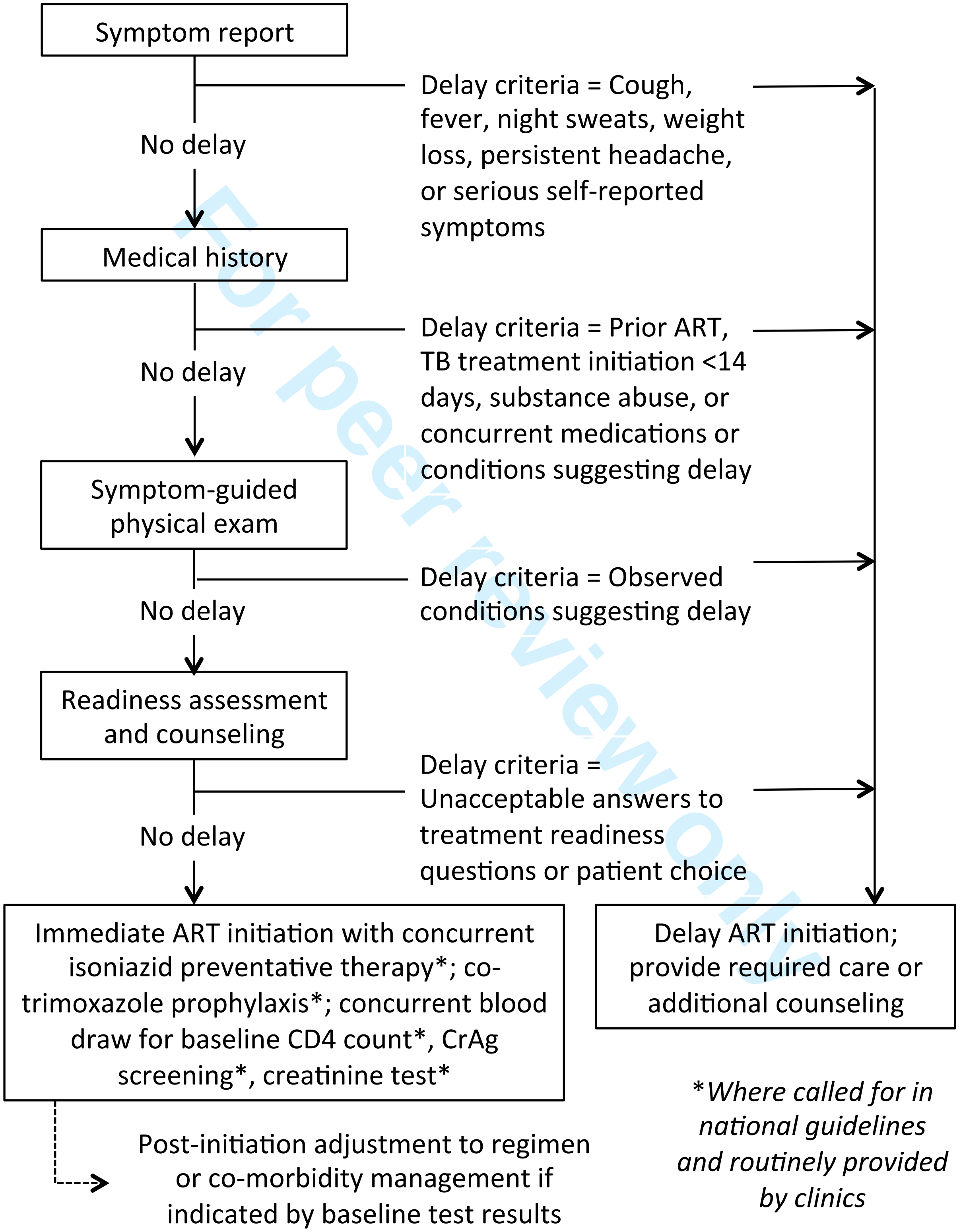

For peer review only

For peer review only - http://bmjopen.bmj.com/site/about/guidelines.xhtml

As shown in Figure 1, the SLATE algorithm uses four screens to assess whether a patient is eligible for immediate (same-day) treatment initiation. Each of the screens is intended to identify specific reasons that a patient might *not* be eligible, including current symptoms of opportunistic infections, previous experiences or behaviors that indicate a need for additional treatment readiness or adherence support, and the patient's own concerns about starting treatment. The algorithm captures all components of treatment eligibility screening currently used in routine care in the study countries with the exception of pre-initiation laboratory results. It follows national guidelines for detection of co-morbidities, which vary by country; some countries call for a CrAg test for patients with CD4 counts < 100, for example, while others do not conduct routine CrAg screening.

The purpose of the SLATE algorithm is to create a standardized approach to collecting and interpreting a minimum set of patient data that will avoid delaying treatment initiation for the majority of patients who are eligible for immediate ART, while deferring initiation in the minority who should not start immediately. If successful, it is expected to increase overall uptake of ART and viral suppression in the population of HIV-infected adults eligible for ART in sub-Saharan Africa.

4. Objectives

The SLATE study is a pragmatic, individually randomized evaluation to determine the effectiveness of the SLATE algorithm in increasing ART initiation, compared to standard care, among non-pregnant adult patients. Patients will be individually randomized to either an intervention arm, in which the SLATE algorithm will be implemented, or a standard arm, in which they will receive standard treatment initiation.

4a. Primary objectives

The primary objectives of this study are to:

1. Compare the proportion of study-eligible patients who initiate ART within 28 days of study enrollment between HIV-infected patients offered immediate ART initiation under the SLATE algorithm and patients offered standard ART initiation.

2. Compare the proportion of study-eligible patients who initiate ART and are alive, in care, and retained on ART by eight months after study enrollment between HIV-infected patients offered immediate ART initiation under the SLATE algorithm and patients offered standard ART initiation.

4b. Secondary objectives

The secondary objectives of this study are to:

3. Compare the proportion of study-eligible patients who initiate ART within 14 days of study enrollment between HIV-infected patients offered immediate ART initiation under the SLATE algorithm and patients offered standard ART initiation.
4. At sites where viral load data are available, compare the proportion of study-eligible patients who initiate ART and are virally suppressed eight months after having an HIV test or enrolling in HIV care
5. Compare the proportion of study-eligible patients who initiate ART and are alive, in care, and retained on ART by 14 months and 16 months after study enrollment between HIV-infected patients

For peer review only - http://bmjopen.bmj.com/site/about/guidelines.xhtml

offered immediate ART initiation under the SLATE algorithm and patients offered standard ART initiation.

6. Estimate the proportion of HIV-positive patients presenting at study clinics and not yet on ART who are eligible or ineligible for immediate initiation using SLATE algorithm criteria.
7. Describe reasons for ineligibility for immediate initiation, among those found ineligible in the intervention arm.
8. Estimate average time interval in days between study enrollment and ART initiation for each study arm.
9. Describe self-reported patient preferences on the speed and timing of ART initiation.
10. Estimate costs to patients of implementing the SLATE algorithm, compared to standard care.
11. Estimate costs to providers of implementing the SLATE algorithm, compared to standard care.

5. Study Design

5a. Overview

The study will be an individually randomized evaluation that will collect data from consented study subjects using questionnaires, a brief physical examination, a venous blood draw where not already done under routine care, and review of routine medical records. Patients randomized to the intervention arm will be initiated on ART if eligible under SLATE or referred for standard care if not eligible under SLATE. Health facility information that does not pertain to human subjects (e.g. unit costs) will also be collected. Figure 2 summarizes study design and procedures.

For peer review only

Figure 2. SLATE study procedures

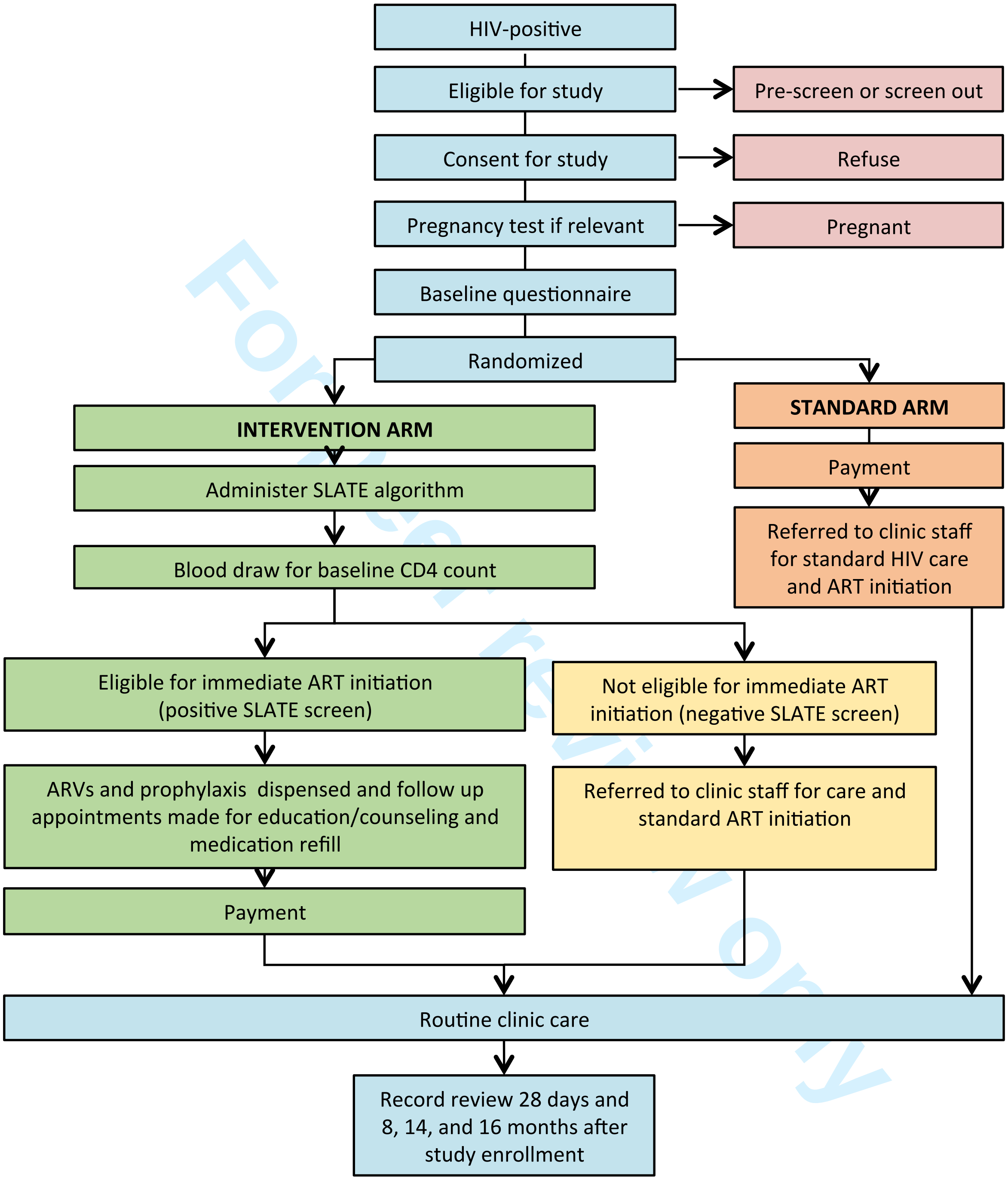

5b. Study sites

The study will be conducted at three high-volume, public sector clinics in each study country. We selected sites that have both a relatively high volume of potentially study-eligible patients, to reduce the time required for enrollment, and have service delivery infrastructure and staff that are typical of the public sector, so that results will be broadly generalizable within each country.

For peer review only - http://bmjopen.bmj.com/site/about/guidelines.xhtml

Sites were selected in collaboration with local study teams and in consultation with government health authorities in each area. A letter confirming government authorization for each site to participate in this study will be submitted to the BU and local IRBs prior to study enrollment at that site.

The proposed sites in each country are:

South Africa:

- OR Tambo Primary Health Clinic, City of Johannesburg
- Alexandra Community Health Centre, City of Johannesburg
- Jabulani Dumani Community Health Centre, Ekhurhuleni Metro

Kenya:

- Kericho County Referral Hospital, Kericho
- Kapsabet County Referral Hospital, Kapsabet
- Kombewa County Hospital, Kombewa

5c. Study population

The study population will be all non-pregnant HIV-infected adults who are not yet on antiretroviral therapy.

Inclusion criteria:

- Adult patients (≥18 years) (initiating children on ART is likely to require additional information, making the SLATE algorithm less applicable to pediatric populations)
- Confirmed HIV-positive test result at any time (may have been diagnosed previously)
- Not currently on ART (three-drug combination)
- Presented at the study clinic for any HIV-related reason, including an HIV test, pre-ART monitoring, or ART initiation

Exclusion criteria:

- Pregnant (pregnancy is an exclusion criterion because treatment guidelines for pregnant women differ from those for non-pregnant adults; most pregnant women are diagnosed with HIV and initiated on ART in antenatal clinics, not general adult HIV clinics)
- Not intending to return to this clinic for further HIV care in the coming year (i.e. intends to seek further care somewhere else)
- Not physically, mentally, or emotionally able to participate in the study, in the opinion of the investigators or study staff
- Not willing or able to provide written informed consent to participate in the study
- Previously enrolled in the same study

All the study countries have adopted a policy of “treat all” or “test and treat”, so that all SLATE study subjects will also be eligible for ART under national guidelines. Although implementation of this policy may not be complete and may vary by site, we will enroll all patients who would be eligible under expected treat all guidelines regardless of actual clinic-specific practice at the time of the study. This will

For peer review only - http://bmjopen.bmj.com/site/about/guidelines.xhtml

ensure that the SLATE study can generate information for a future in which "treat all" is the standard in most places.

Patients will be followed passively through medical record review for up to 16 months after study enrollment.

6. Enrollment and Preparation

6a. Pre-screening

Patients presenting at a study clinic who are HIV-infected but not yet on ART will be eligible for study pre-screening. This includes patients diagnosed earlier and patients newly diagnosed on the same day. At each study site, the site's staff (counselors, clerks, and clinical staff, as appropriate to each site) will inform potentially eligible patients about the study at visit registration, which will be defined as the patient's first point of contact with clinic staff upon arriving at the clinic. Patients who express willingness to learn more about the study will be referred to a study assistant for screening and consent. Patients will be assured that they will not "lose their place" in the clinic queue by agreeing to referral to the study assistant; upon completion of interaction with study staff, the study assistant will escort patients who do not want to participate or who were randomized to standard care to return to their original place in the queue (or front of the line in case their place was lost) to continue with their routine clinic visit.

Where feasible, clinic staff who refer patients to the study assistant will be asked to complete one row in a pre-screening logbook (Appendix 1) for each patient they inform about the study, to allow the study to collect aggregate statistics about the overall patient population and compare it to the screened population. Fields that will be recorded in this logbook are date, sex, age in years, and primary reason for clinic visit (HIV test, CD4 count, pre-ART monitoring, adherence education session, ART initiation). No identifiers of any kind will be collected in this logbook, which is intended to provide only aggregate information. We anticipate that use of the logbook will be feasible at some study sites and some first points of contact at those sites but may not be feasible at others, if clinic staff are too busy or do not remain in a fixed location in the clinic. Where the logbook cannot be used, clinic staff will follow the same procedures for information patients about the study, but no record will be kept. Data analysis requiring pre-screening data will be limited to sites where the logbook is used.

6b. Screening

Upon referral to the study assistant, patients will receive a more complete description of the study, including the details of why it is being done, study procedures, and the need for written informed consent. They will be assured that participation is voluntary and that they can withdraw from the study at any time, without affecting the quality of care provided by the site. They will also be offered the opportunity to ask questions. If the study assistant concludes that all other inclusion/exclusion criteria have been met, patients will then be asked to provide written informed consent to participate, as described below. The study assistant will complete a screening form (Appendix 2) to confirm study eligibility for each patient screened. The screening form will not collect any identifiable information pertaining to individual patients prior to receipt of written informed consent.

Patients will be screened consecutively, as they arrive at the study site. Due to the volume limitations of study staff, however, recruitment may be halted during intervals when the study assistant and/or study

For peer review only - http://bmjopen.bmj.com/site/about/guidelines.xhtml

clinician are already busy. We anticipate that clinical data collection will take no more than 30 minutes per patient, allowing us to enroll and collect data from roughly 8-10 patients per day using a single study clinician per site.

6c. Consent

Written informed consent will be obtained from all study participants after screening and prior to study enrollment. The study information sheet and consent form (Appendix 3) will be translated into local languages most commonly spoken by patients at the study sites. Translated consent documents and attestation of consent accuracy will be submitted to the BUMC IRB and local IRB prior to use with any study subjects. Potential subjects will be asked if they would prefer to read the information sheet or have it read to them by the study assistant and will be given the opportunity to ask questions about the study. Those who provide written informed consent will be enrolled. Those who refuse consent will be referred back to the clinic staff to complete their routine visit, and the study assistant will indicate the refusal on the screening form.

6d. Pregnancy test

Because SLATE is not intended for pregnant women, we will conduct a rapid pregnancy test for female patients following consent. Women who have already reported being pregnant will have been screened out prior to consent; the pregnancy test will identify those who were not aware of being pregnant. Patients who have a positive pregnancy test will be withdrawn from the study at this time and escorted to the clinic registration desk to begin antenatal care and PMTCT.

6e. Questionnaire

The study assistant will administer a short questionnaire documenting the subject's basic demographic and socioeconomic characteristics, costs incurred per clinic visit, and preferences regarding ART initiation. As the questionnaire elicits only simple responses and will be completed by the study assistant not the subject, we do not intend to translate the questionnaire itself into subjects' first languages. Instead, for subjects who do not speak English, the assistant will ask the questions in one of the commonly-understood languages used by the study population, and then record the answers in English on the questionnaire. The questionnaire is included in the study case report form in the appendix to this protocol.

6f. Randomization

After the questionnaire has been completed, patients will be randomized to the intervention or standard groups of the study. Randomization envelopes will be generated in blocks of 6 and kept at the study sites. For each enrolled patient, the study assistant will open the next envelope in sequential order, read the randomization group, and record it on the case report form. The study assistant will then inform the patient of the randomization assignment.

Following randomization, standard group patients will be escorted to the clinic registration desk to resume their routine clinic visit. The study assistant will repeat the explanation of next steps in the study, which are limited to review of medical records by the study team, provide study payment as described below, and thank them for their willingness to participate in the study. The study team will have no further personal contact with patients in the standard group.

For peer review only - http://bmjopen.bmj.com/site/about/guidelines.xhtml

The study assistant will introduce intervention group patients to the study clinician for algorithm data collection. The study clinician will be a nurse, medical officer, or clinical officer at the same level as the individual responsible for ART initiation under standard care at the study site.

7. Intervention

7a. Algorithm data collection

The SLATE algorithm (illustrated in Figure 1 above) calls for four types of screens: a symptom report, medical history, brief physical exam, and readiness assessment. Each of these is described below and further detail is provided in the case report form appended to this protocol. A negative response to a screen indicates that the patient "screens out" and referall for additional care (consultation, laboratory test, counselling, other) is recommended; if all responses are positive, then the patient "screens" in for immediate initiation under SLATE.

1. Symptom report. Each patient will be screened for current cough, fever, night sweats, and weight loss, which comprise the standard, WHO-recommended TB symptom screen[8], and for persistent (continuous) headache for more two days, which is a symptom of cryptococcal meningitis[9,10], and will be asked about any other symptoms that the patient has noticed.

2. Medical history: To identify individuals who are likely to face problems with medication-taking or adherence, patients will be asked if they have ever been on ART previously, started TB treatment in the past two weeks, are taking concurrent medications for epilepsy or warfarin, which can interact with ARVs, or have a history of substance abuse.

3. Physical exam: A brief, symptom-guided physical exam to look for causes of symptoms or any other evident conditions that could postpone ART initiation will be conducted.

4. Readiness assessment and counselling: A published review of readiness instruments for HIV initiation found that none was notably successful in predicting patient readiness for ART, as indicated by adherence once on treatment[11]. Despite this, there is general consensus that explicit attention to readiness is an important component of treatment initiation. Drawing on the published literature on stage-of-readiness scales[12], we have created a short instrument which we expect to serve primarily as an invitation to patients to raise concerns or questions about treatment readiness with the clinician conducting the assessment. Following the questions, the study clinician will have a brief conversation with the patient to confirm that the patient feels ready to start treatment immediately, understands what happens next, and has no further questions or concerns.

If SLATE were used in routine care, we expect that the screens would be administered sequentially, so that a negative response on the first screen would cause a patient to screen out without having to complete the second screen, and so on. Patients would progress to the next screen only after being found positive on the previous one. For purposes of this study, however, we will administer all four screens, to obtain a complete algorithm data set for each patient in the intervention arm.

We anticipate that for the vast majority of patients, the first three screens will take less than 5 minutes each to administer and the last screen less than 10 minutes. Patients who screen out will require more time, and we therefore estimate needing an average of up to 30 minutes of clinician time per patient in the intervention am.

For peer review only - http://bmjopen.bmj.com/site/about/guidelines.xhtml

7b. Blood draw

Cryptococcal meningitis and other co-infections are more likely in patients with very low CD4 counts. Although new national guidelines in the study countries do not require the results of a CD4 count to establish treatment eligibility, most countries will continue to use CD4 counts to monitor patient condition. To avoid asking intervention group patients to wait in the clinic's queue for a blood draw, the study clinician will do a standard venous blood draw, following the same procedures that the clinic would otherwise use. Blood samples will be tested by the laboratories that serve the clinics, following routine procedures, and results will be placed in routine medical records so that the study clinics do not have to repeat the CD4 count for the same patients. The clinic may also use the blood sample for other baseline tests recommended in national guidelines, such as creatinine, hemoglobin, and for patients with CD4 counts under 100 cells/mm^3, cryptococcal antigen (CrAg). The clinic will follow routine procedures for laboratory tests; the study will only be responsible for drawing the required blood sample.

Study staff will monitor CD4 count results for all patients enrolled in the intervention arm. Under national guidelines in both countries, any patient who has a CD4 count under 100 cells/mm^3 and a positive cryptococcal antigen (CrAg) screening result should begin presumptive treatment for cryptococcal meningitis. Results of the CD4 count and CrAg screening test will only be available several days after study enrollment, however. To facilitate proper follow up of any intervention arm patient who does have a positive CrAg screening test, study staff will review CD4 count results for intervention arm patients as soon as the results are available at the study clinics. The study team will alert the clinic to any patient who has a CD4 count under 100 and request that the clinic trace the patient and provide follow up care as guidelines mandate.

7c. Referral of patients who screen out

After completing all four of the SLATE screens, the study clinician will fill out a brief referral letter that reports any substantive findings, such as TB symptoms or a physical exam observation. Intervention arm patients who screen out (have negative responses on any of the SLATE screens) will be referred to routine clinic care to address the issue(s) identified and initiate ART under standard care. The study clinician will discuss and explain the concern(s) with the patient and then escort the patient to the registration desk to continue with a standard clinic visit. The patient will be given the referral letter and encouraged to give the letter to the clinic nurse or medical officer. Patients who do not wish study findings to be shared with clinic staff will not be required to pass on the letter. Patients who are referred to routine care due to a positive SLATE screen will remain in the intervention arm, have the same study follow up as other intervention arm participants, and be analyzed as part of the intervention arm.

For patients who screen in (have positive responses on all the SLATE screens and are eligible for immediate ART initiation) give their agreement, referral letters will be placed in their clinic files along with other source documentation, such as the regular ART record that must be filled out for all new ART patients. Letters will not be filed for patients who do not agree.

7d. Dispensing of ARVs and prophylaxis

Patients who have a full negative SLATE screen (no reasons for delay) will immediately be offered their first supply of ARV medications. While making the offer to dispense, the study clinician will confirm that

For peer review only - http://bmjopen.bmj.com/site/about/guidelines.xhtml

the patient still wishes to start ART immediately, answer any questions the patient may have, and reiterate the importance of returning for the patient's next appointment and medication refill.

In addition to ARVs, national guidelines call for prescribing isoniazid preventive therapy (IPT) and/or co-trimoxazole prophylaxis to new HIV patients. The study clinician will also write prescriptions for these medications. Where possible, the study clinician will have a stock of medications in the study room, so that the patient does not need to go anywhere else in the clinic to obtain their medications. If this is not allowed, the study clinician or study assistant will escort the patient to the clinic pharmacy to obtain the medications without waiting in a queue. The clinician or study assistant will then escort the patient to the clinic registration desk to schedule the next appointment, which will include participation in one of the clinic's routine adherence education sessions.

7e. Completion of direct study interaction and payment

Direct study interaction with study subjects will cease as follows:

- *Intervention group (Track 1)*: Upon completion of the ART initiation steps described above, after which subjects will be managed by the study site following standard procedures.

- *Standard group (Track 1)*: after randomization, at which point subjects will be managed by the study site following standard procedures.

From these points on, all subjects, regardless of which type of initiation they received, will be managed by the site following its routine procedures for pre-ART and ART care. Because this study evaluates routine practice and uses retention in care as an outcome, any further study interaction with subjects would affect the results, in particular loss to follow-up. There will therefore be no post-baseline study visits, only routine care visits not involving study personnel. Standard procedures will also be implemented by the study site (not the study team) for study subjects who miss scheduled visits.

The last step in study interaction for both groups will be payment. Reimbursement to consented study subjects of the equivalent of $5-$15 will be provided as a token of appreciate for study participation. The exact amount will be determined in consultation with the study sites and local study teams. The reimbursement will be in the form of cash in Kenya and a voucher to a nearby supermarket and general goods store in South Africa.

8. Medical Record Follow Up

Study subjects in both arms will be followed passively through review of routine medical records kept by the study sites for up to 14 months after study enrollment. This will capture up to 1 month for treatment initiation, up to 12 months of follow up, and up to 1 month for a 12-month routine visit to be made.

The purpose of medical record follow up is to determine which patients enrolled in the study did go on to initiate ART and after what time interval, their early outcomes on treatment, and resource utilization (cost) of initiating ART.

Specific data fields to be collected from medical records are listed under Data Collection below.

For peer review only - http://bmjopen.bmj.com/site/about/guidelines.xhtml

9. Data Collection and Management

9a. Sources of data

The study will collect data from five sources, as follows:

1. Pre-screening logbook. Aggregate statistics about the number of potentially eligible patients not screened for the study will be calculated from logbooks completed by clinic staff.

2. Screening form. Screening forms will be completed to confirm study eligibility and consent.

3. Case report form. The study assistant and study clinician will use the CRF to record all information gathered directly from the patient, including the questionnaire and four SLATE screens.

4. Medical records. Routinely collected medical record data will be abstracted from medical records maintained by the study site and by central repositories (e.g. laboratory database) in electronic and paper format.

5. Facility-level records. We will use aggregate information from the study sites to estimate the overall proportion of patients presenting at each clinic who could start ART immediately if SLATE were used in routine care and the resources that would be required.

9b. Pre-screening logbook

As explained above, where feasible we will ask clinic registration staff to complete one row of a pre-screening logbook for each patient who is offered referral to the study assistant for screening. The logbook will have only five fields: date of visit, sex, age in years, primary reason for clinic visit (HIV test, CD4 count, pre-ART monitoring, adherence education session, ART initiation), and whether the referral to the study assistant was accepted (yes/no). No identifiers of any kind will be collected in this logbook, which is intended to provide only aggregate information. Data from the logbook will be used to estimate the total volume of patients who could benefit from SLATE if it were adopted as standard care and to compare the characteristics of patients who do enroll in the study with those of the overall patient population at the site.

9c. Screening form

The study assistant will complete a screening form for each patient referred after pre-screening. The screening form will record whether the patient meets each inclusion and exclusion criterion and provides written informed consent. For patients enrolled, the Study ID number and medical record number will be added to the screening form, which will become part of the case report form for study subjects. For patients who screen out, it will record the reason for ineligibility.

For female patients, the results of the pregnancy test conducted after consent will be recorded on the screening form, as pregnancy is an exclusion criterion for the study. No case report form will be opened for patients who have positive pregnancy tests.

9d. Case report form

For peer review only - http://bmjopen.bmj.com/site/about/guidelines.xhtml

The case report form will have eight sections. Sections 1, 7, and 8 will be completed for standard group patients. All sections will be completed for intervention group patients.

1. A short, closed-ended questionnaire to be administered by the study assistant after consent. The questionnaire will elicit basic information about:

   - Patient characteristics (age, sex, marital status, household composition, duration living in current location, education)
   - HIV-related information (familiarity with clinic, time of diagnosis, acquaintance with others with HIV or on ART)
   - Activities and employment
   - Costs to patient of making a clinic visit (transport costs, loss of income, etc.)
   - Patient's preferences regarding ART initiation (single visit, multiple visits, intervals between).

2. Symptom report. The symptom report form will record yes/no to the presence of the symptoms listed above and provide a space for the study clinician to describe or comment on symptoms and to state whether the patient is ineligible for immediate ART initiation due to symptoms.

3. Medical history. The medical history form will record answers to the medical history questions and provide a space for the study clinician to describe or comment on these answers and to state whether the patient is ineligible for immediate ART initiation due to medical history.

4. Physical exam. The physical exam form will record findings of the physical exam and provide a space for the study clinician to describe or comment on these findings and to state whether the patient is ineligible for immediate ART initiation due to the outcome of the medical exam.

5. Readiness assessment. The readiness assessment form will record answers to the readiness questions and provide a space for the study clinician to describe or comment on these answers and to state whether the patient is ineligible for immediate ART initiation due to readiness.

6. Blood draw. The blood draw form will confirm that a blood sample was taken and record the identification number or bar code used to identify the sample on the CD4 count laboratory order form.

7. Patient identifiers. The identification form will capture identifying information about the subject, including name, date of birth, clinic ID number, and national identification number. This information will not be entered into the study database but will be used only for matching CRF and medical record data.

8. Completion form. The last page of the CRF will confirm data collection and close the study visit for the patient.

9e. Medical record data

Once the CRF has been completed, all remaining patient-level data for the study will be collected from routinely maintained medical records. The format of these records will vary by study country and site. Some sites will use electronic medical records, while others will rely on paper-based records and registers or a combination of electronic and paper. We will access all available records for each study

For peer review only - http://bmjopen.bmj.com/site/about/guidelines.xhtml

patient, regardless of format or storage location, to try to obtain as complete a study data set as possible. The fields we will aim to capture are listed below.

To be used to link electronic and paper-based records to study identification number only:

- Electronic medical record system number
- Clinic file numbers
- National ID number
- Name
- Date of birth
- Sex

All subjects at clinic visit on date of study enrollment:

- Date of positive HIV test (if available)
- CD4 count (most recent)
- Date and result of TB symptom screen, if done
- Date, type, and result of TB test(s) (current and previous, if any)
- TB treatment, if offered (date of initiation)
- WHO stage and clinical conditions

Subjects who are eligible for ART initiation at the study clinic:

- Reasons for delaying ART initiation, if relevant
- Date ART initiated (first dose of drugs dispensed)
- First-line regimen prescribed

For all clinic visits for duration of study follow up, from study enrollment to the follow-up endpoints indicated above:

- Date of visit
- Primary reason for visit (e.g. pre-ART visit, scheduled medical visit, medication pickup, unscheduled medical visit)
- Types of professionals seen (e.g. nurse, doctor, pharmacy assistant, counselor)
- Group and individual sessions attended (e.g. wellness course, adherence education)
- Numbers and types of all lab tests conducted (including CD4 counts, viral loads, CrAg test, blood tests to determine ARV regimen)
- Other procedures (e.g. x-rays) performed
- Medications prescribed and dispensed (ARV and non-ARV, including isoniazid and cotrimoxazole)
- Admissions for inpatient care since previous visit, if recorded
- Details of inpatient care received (facility, number of days, medications, lab tests, etc.) if recorded

At follow-up endpoints (6 and 12 months after ART initiation or comparable timepoint for those who do not initiate ART):

- Date and result of most recent viral load test
- Status at clinic (in care / died / transferred to another site / stopped care / lost to follow up, defined as > 1 month late for last medication pickup)
- New or recurring HIV-related conditions
- Date and cause of death if applicable
- Date and result of most recent CD4 count

For peer review only - http://bmjopen.bmj.com/site/about/guidelines.xhtml
For peer review only

9f. Study identification numbers and linking of records

Upon consenting to participate in the study, each subject will be assigned a random study identification number. This will be a five-digit number in which the first digit indicates the country and the remaining four digits will be a sequential number that does not provide any identifying information.

A password-protected, encrypted linking file will be created in REDCap to link study identification numbers to subjects' names, dates of birth, national identity numbers, and clinic file numbers. These fields are needed to ensure that study ID numbers can be correctly matched to the identification numbers used in the sites medical records. When a subject has been matched to a clinic record, the clinic record number will also be added to the linking file. The linking file will be a separate document from any other study files. Access to the linking file will be limited to the study team, and the file will be stored in a secure location separate from the study database.

9g. Data entry and storage

Screening and CRF data will be entered by study staff onto Android-based tablet computers programmed for data collection using the REDCap mobile app. The REDCap system provides a secure, web-based platform for data entry that allows real time monitoring, querying, and quality control of data by an off-site data manager. It allows access to data to be assigned and restricted to individual study staff as needed and creates audit trails to monitor appropriate access. The mobile app allows data to be entered offline and then uploaded to a central server when an internet connection is available. Records are encrypted upon entry and can be removed from data capturers' tablets once they have been uploaded to the server, reducing the chances of breach of confidentiality if a tablet is lost or stolen. REDCap is supported by the Boston University IT office (https://redcap.bumc.bu.edu/). It has previously been used successfully in South Africa, where the University of the Witwatersrand is a REDCap Consortium partner.

Medical record data for study subjects will be downloaded from electronic medical record systems where available. The lead data analyst will be responsible for ensuring that study identification numbers are correctly matched to the record numbers in the database. In instances where the data analyst cannot match a study ID number to an electronic medical record number using only the fields in the study linking file, a query will be sent to the on-site study staff, who will access the clinic's paper-based patient files to determine whether the subject ever initiated ART and, if so, locate the correct medical record number.

At sites where all or some data are recorded only in individual paper-based files, a note or tab indicating that a patient is a participant in the study will be attached to the cover of each subject's file, to assist site and study staff in locating study subjects' files for data extraction. At regular intervals, files will be pulled and the data fields listed above entered into the study database by study staff. All data entry will take place at the study clinics or other locations where the files are stored; no hard copies of patient records will be taken off site by the study team.

Data will be retained in secure study databases until completion of the study, including completion of all data analysis and report writing associated with the study and closing of the IRB-approved protocol. Study data sets and other electronic files containing study data will then have all identifiers removed

For peer review only - http://bmjopen.bmj.com/site/about/guidelines.xhtml

and where possible will be made available for use of other researchers through a repository such as Dryad. Hard copies of study documents will be shredded.

10. Staff Supervision and Training

In each study country, the local study team will include a principal investigator, study coordinator, and site-based study clinicians, assistants, and data capturers. The local PIs will be responsible for training and supervising their teams, under the guidance of the overall study PI and co-investigators. The study clinicians who will implement the intervention will have the qualifications required for ART initiation in each study country. Study assistants who will administer consent and the questionnaire will also be trained as HIV counselors to ensure that they are sensitive to the needs of HIV-positive study subjects.

All members of the study team will be trained in and adhere to the standards laid out in the Belmont Report and NIH guidelines for research and in research ethics applicable to South Africa. Human subjects ethics training will be repeated on an annual basis to ensure that the study team's understanding of research ethics is current.

11. Sample Size

Using results of the RapIT randomized trial we recently conducted in South Africa, we estimate that 65% of treatment-eligible patients will be initiated on antiretroviral therapy and retained on ART in the standard group, and we consider an increase to 80% to be programmatically important. Using an α of 0.05, power of 90%, 1:1 randomization, and an uncorrected Fisher's exact test, this requires a minimum sample size of 197 patients per group, which we will increase to a maximum of 240 to ensure sufficient power if patients withdraw or are found ineligible after consent. The sample size will thus be a maximum of 480 per country, or a maximum of 960 for the two-country study.

12. Outcomes and Data Analysis

12a. Outcomes

The primary outcomes for the study are:

1. The proportion of patients initiated on ART within 28 days of study enrollment. While national guidelines recommend ART initiation within two weeks of a patient's first clinic visit or HIV diagnosis, recent studies suggest that 28 days is a more realistic deadline for routine care.

2. The proportion of patients who initiate ART and are alive, in care, and retained on ART eight months after study enrollment. Eight months was selected to allow up to 1 month (28 days) to initiate ART, six months of follow up after treatment initiation, and up to 1 month to return for the six-month routine clinic visit.

These outcomes were proposed in a paper published by the investigators based on the MATI technical consultation described in protocol section 3c.[7]

Secondary outcomes will include:

For peer review only

3. The proportion of patients initiated on ART within 14 days of having an HIV test or enrolling in HIV care.
4. The proportion of patients who initiate ART and are virally suppressed eight months of having an HIV test or enrolling in HIV care between HIV-infected patients offered immediate ART initiation under the SLATE algorithm and patients offered standard ART initiation.
5. The proportions of patients who initiate ART and are alive, in care, and retained on ART 14 and 16 months after having an HIV test or enrolling in HIV care. Fourteen months extends the primary outcome endpoint, which is at 8 months, by an additional six months. Sixteen months allows loss to follow up at 12 months to be defined as three months late for the 12-month visit, which is a common endpoint in the literature (16 = 1 month to initiate ART + 12 months' follow up + 3 months to attend 12-month visit).
6. The proportions of HIV-positive patients presenting at study clinics and not yet on ART who are eligible and ineligible for immediate initiation using SLATE algorithm criteria.
7. Reasons for ineligibility for immediate initiation, among those found ineligible in the intervention arm.
8. Average time to ART initiation (days) for each arm.
9. Patient preferences on the speed and timing of ART initiation.
10. Costs to patients of ART initiation under standard and intervention procedures.
11. Costs to providers of ART initiation under standard and intervention procedures.

12b. Analytic methods

We will first conduct descriptive analyses to allow us to determine the characteristics of the sample we have enrolled. We will then analyze the specific outcomes as follows:

Primary outcomes 1 and 2 and secondary outcomes 3, 4, and 5:

For all primary and secondary clinical outcomes, the analytic approach will be the same. All analyses will be by intention-to-treat: subjects will be analyzed according to the intervention they were supposed to receive, whether or not they adhere to the defined intervention. This includes patients randomized to the intervention arm who are screened out of immediate ART initiation by the SLATE algorithm; they will remain in the intervention arm for data analysis. Primary analyses will be pooled across study countries, though we will also conduct stratified analyses by country to look for country-specific differences.

The analysis will begin with a simple comparison of the two treatment groups with respect to baseline predictors of outcomes to look for any imbalances. These potential confounders include demographic (e.g. age, sex) and socioeconomic (education, distance from clinic, employment, etc.) variables, medications, baseline CD4 counts, and low BMI, among others.

We will then conduct a crude analysis comparing the proportion of patients achieving each dichotomous outcome by group and estimating crude risk ratios and crude risk differences and their corresponding 95% confidence intervals. This will be our primary analysis as we anticipate that randomization of over 1000 subjects should lead to balance in baseline covariates. Should any important imbalances occur by chance, we will proceed with an adjusted model as described below for the country-specific analyses.

Next, we will look for effect modification by important predictors of each outcome. The primary modifier will be country, as practices for care and treatment may differ across the different countries.

For peer review only - http://bmjopen.bmj.com/site/about/guidelines.xhtml
For peer review only

We will also look for differences in effects stratified by age, sex, baseline BMI, CD4 count, and any other important demographic and clinical predictors of outcomes identified. Our analysis for effect modification will use a simple stratification of the primary analysis by the potential modifier and report crude risk differences and risk ratios and their corresponding 95% confidence intervals.

When stratified by these potential effect modifiers, because the sample sizes within groups will be smaller, it is possible that some baseline covariates may be imbalanced by treatment arm. To adjust for potential differences by baseline covariates, a log-linear regression model will be used to estimate adjusted risk ratios. Variables considered to be important in the univariate stage will be included in multivariate models. Adjusted analyses will include covariates which are unevenly distributed across treatment groups and which could plausibly affect retention or ART initiation. Each of these models can then be used to look for patient-level predictors of treatment uptake and retention in care.

Secondary outcomes 6-9:

Secondary outcomes 6, 7, 8, and 9 are descriptive in nature. These will be described using frequencies, stratified by country, site, and patient characteristics as numbers allow.

Secondary outcome 10:

We will estimate the average cost to patients per clinic visit using questionnaire responses, including transport, substitute labor, and other costs. This cost will then be multiplied by the average number of clinic visits observed in each study arm to generate a total average cost to patients of ART initiation under each model (SLATE and standard). Costs will be reported as means (standard deviations) and medians (IQRs) in local currencies and dollars.

Secondary outcome 11:

At 8 months after study enrollment, all resource usage will be extracted from subjects' medical records and case report forms. Unit costs will be obtained from external suppliers and the site's finance and procurement records and applied to the resource usage data to provide a cost per study patient. Costs will be measured from the provider perspective and will include the cost of all resources utilized for each study patient, including drugs, laboratory tests, outpatient visits, and fixed costs such as building space, equipment, and management staff. We will estimate the average cost to the provider per patient achieving each primary outcome. The average cost per outcome will then be compared between intervention and standard initiation groups to provide an estimate of the cost-effectiveness of the two strategies. Costs will be reported as means (standard deviations) and medians (IQRs) in local currencies and dollars.

12c. Dissemination of findings

The results of this study will be disseminated as widely as possible in the study countries, where new strategies for increasing the efficiency of treatment initiation are activity sought. A full report will be made to the study sites and other local stakeholders and will be circulated widely and posted on our website. We will also develop a short briefing document to send to the national and provincial departments of health, donor agencies, and other interested organizations, and we will present the results at relevant conferences domestically and internationally. One or more journal manuscripts will be submitted to an appropriate peer-reviewed international journal.

For peer review only - http://bmjopen.bmj.com/site/about/guidelines.xhtml

13. Human Subjects Considerations

13a. Risks and protection against risks

For this study, we will not collect any biomedical samples that would not be collected as part of routine care, nor will we implement any clinical procedures that are not routinely carried out under standard of care. The strategy being evaluated merely reorganizes data collection and accelerates the timing of events. The only non-routine data generated for the study itself will be responses to questionnaires. Study staff will conduct a brief physical exam and do a blood draw for intervention arm subjects, but both are procedures that are routinely performed and carry little risk. We therefore believe that our study poses no physical risks to subjects beyond those routinely encountered. It may, however, pose risks associated with loss to HIV care, emotional distress, and breach of confidentiality.

Emotional distress

Risk: By necessity, the study population for this study will include some patients who have been newly diagnosed with HIV. Interacting with them in order to explain the study and confirm eligibility before requesting written informed consent may cause some emotional distress for some potential subjects.

Protection: To minimize any emotional distress caused by interacting with study staff or enrolling in the study, we will train the site's staff to emphasize when they introduce the study to potential subjects that referral to study staff and enrolling in the study are completely voluntary and that those who do not wish to enroll will receive exactly the same care as the study site would otherwise have provided. Potential subjects will also be told that they can discontinue participation at any time, even after consenting. Study staff will be trained to look for signs of emotional distress and instructed to terminate or postpone the enrollment process if subjects appear distraught.

Loss to HIV care

Risk: The study aims to initiate on ART patients who might otherwise be lost to HIV care. Some patients lost before or after ART initiation will seek care at other sites, but many will not and will postpone or end all HIV care and treatment, some until they become more seriously ill, others forever. Although the SLATE strategy is expected to increase the proportion of patients who do initiate ART, there is a possibility that patients who accept immediate ART initiation under SLATE will subsequently discontinue treatment at a greater rate than do those who receive standard ART initiation. In our prior study of same-day initiation (RapIT), we found that a higher proportion of patients in the intervention arm than in the standard arm were lost to care, but we believe that most of these were patients who otherwise would never have started treatment at all, had only standard initiation been offered. We therefore expect the SLATE intervention to have a net positive impact on our primary outcomes. We cannot be certain of this result in advance, however.

Protection: Because this is an evaluation of routine practice and retention in care is a study outcome, no efforts will be made to retain patients in the study, beyond those already routinely undertaken by the study site clinics as part of standard care. Intervention arm patients will

For peer review only - http://bmjopen.bmj.com/site/about/guidelines.xhtml

be encouraged to remain in the clinic on the day of study enrollment if a routine ART adherence class or club is available on that day. Study staff will escort intervention arm patients to clinic registration desks to confirm that each patient has made an appointment for a follow-up visit and that the first follow-up visit includes the clinic's regular ART adherence activities (e.g. individual counselling, group education sessions, etc.). Through these efforts, intervention arm patients will have access to the same adherence support as standard arm patients, though the support will be offered after ART initiation rather than before.

Loss of confidentiality

Risk: Because we must collect identifying information in order to link CRF data with medical records, accidental disclosure of HIV status or other loss of confidentiality is also possible. A high level of stigmatization continues to inhibit the disclosure of HIV status in the study populations. Data collected in during the study visit or from clinic records could be disclosed and reveal a person's HIV status.

Protection: Several steps will be taken to protect subjects against the risk of accidental disclosure of HIV status. Individual documents or electronic files (including signed consent forms, case report forms, and the linking file) which could associate patients with an HIV study will be kept strictly confidential. Signed consent forms will be stored in locked cabinets away from the study site, with access limited to the senior investigators. CRFs will be held in electronic versions only and will be password-protected. The linking file will also be password-protected, with access limited to study staff. It will be used only for the purpose of linking study identification numbers with clinic medical records.

To protect against other violations of confidentiality, study staff will be trained in expectations that they are not to disclose any information collected in the study to anyone outside the study team. All study staff will be required to pass an ethics certification course, such as the on-line certification offered by the NIH. All study participants will be encouraged to contact the study clinician or clinic manager to report any undesirable conduct associated with the study. These reports will be brought to the attention of the local and international PI, and appropriate steps will be taken to solve the problems, including reporting to relevant ethical review boards.

13b. Benefits

Direct benefits

Study subjects offered immediate treatment initiation will benefit from the opportunity to start ART quickly and with fewer clinic visits and time delays than they would otherwise have incurred. Study subjects offered standard initiation will not receive any direct benefits from the study but will receive the same care that would have been provided in the absence of the study.

Indirect (societal) benefits

The study has the potential to generate substantial indirect benefits to the subjects. The research we will undertake is expected to reveal whether a simplified clinical algorithm can successfully be

For peer review only - http://bmjopen.bmj.com/site/about/guidelines.xhtml

incorporated into procedures for ART initiation, leading to more patients initiating ART and a reduction in patient attrition from care. The results of the study may thus lead to improvements in HIV care and keep patients alive and in care longer.

Ratio of benefits to risks

The knowledge to be generated by this study will allow HIV treatment programs to determine whether a simplified clinical algorithm can be used to determine eligibility of immediate ART initiation. This study will address an important gap in the current knowledge around ART initiation and what can be done to reduce the already high proportion of patients who are being lost from care after testing positive. There is currently almost no information available on how to reduce these pre-ART losses. As explained in the previous section, we believe that the risk to subjects in our study is minimal and is outweighed by the indirect benefits and the potential importance of the findings.

13c. Costs and payments to subjects

There will be no costs to subjects for participating in this study.

Subjects who consent to participate and complete study procedures will receive a payment of $10-$15 (local currency equivalent) to reimburse for their time and inconvenience. This payment will be made in the form of a shopping voucher, cell phone air time, or cash, depending on individual study site circumstances.

13d. Recruitment

Identification of potential subjects will occur during patients' regular clinic visits for any HIV-related service other than monitoring of patients already on ART. At reception and relevant waiting areas at each study site a flier will be posted that informs patients that a study is underway and indicates where to get further information. The flier will be translated into the commonly used languages at the study sites.

The site's post-test counselors, nurses, and registration staff and the study assistant will inform potentially eligible patients about the study while they are awaiting and/or receiving services. They will explain that a study is underway and that patients found eligible are invited to participate. They will be assured that they will not lose their place "in line" at the clinic and will still receive whatever services they came to the clinic for. Patients who indicate that they may be interested in participating in the study will be referred to the study assistant for screening.

13e. Informed consent

Written, informed consent will be sought from all study subjects by a trained study assistant. The informed consent information sheet will describe the nature, goals, and procedures of the study and assure subjects their information will be kept confidential. The consent form will explain:

- That patients will not get to decide which group they will be in, but rather that we will decide by chance and they will have a 50-50 chance of being in either group;
- How study procedures will be different from standard care procedures for those randomized to the intervention group;

For peer review only - http://bmjopen.bmj.com/site/about/guidelines.xhtml

- How we will do follow-up through medical record data collection; and
- That any individual patient can choose not to participate in the study and be offered standard initiation.

The full informed consent information sheet and form will be translated into the most commonly used languages at the study sites. Translated forms and attestations of translation accuracy will be submitted to the IRB prior to any use of the forms. Subjects who unable to read and/or sign the consent form due to illiteracy will be asked to provide a thumbprint mark, in the presence of a witness who will also sign the form. Patients who do not speak English or any of the languages into which the form has been translated will be regarded as unable to provide written informed consent, which is an exclusion criterion for the study, and will not be eligible for study enrollment.

13f. Protection of confidentiality

Subject data will be captured electronically on case report forms programmed on Android tablet computers using REDCap or another similar, secure application. The tablets will be password-protected and files will be encrypted. When not in use, they will be stored in locked cabinets at the study sites or in study team offices. Study subjects will be assigned sequential study ID numbers upon consent, and data files will contain study ID numbers only, without any other individual identifiers.

A password-protected linking file allowing medical record data to be matched to study-generated data will be maintained in a secure location, with access limited to study staff. Medical record data will be downloaded or extracted from paper records on a regular basis, as needed for data analysis. Electronic medical record datasets may include data on all patients at the study clinic, not just those included in the study, as it may not be possible to select only study participants prior to downloading. As soon as datasets are downloaded, the linking file will be used to select study participants and link their records with their study identification numbers. All identifiers will then be stripped from the clinical data and records for all patients not enrolled in the study will be deleted.

14. References

1. World Health Organization. Guideline on when to start antiretroviral therapy and on pre-exposure prophylaxis for HIV. World Health Organization, Geneva, 2015.
2. Barnabas R, Bendavid E, Bershteyn A, et al. HIV mortality by care cascade stage and implications for universal ART eligibility. Conference on Retroviruses and Opportunistic Infections, Boston, MA, Feb 22-25, 2016.
3. Rosen S, Fox MP. Retention in HIV care between testing and treatment in sub-Saharan Africa: a systematic review. PLoS Med 2011; 8: e1001056.
4. Lahuerta M, Ue F, Hoffman S, et al. The problem of late ART initiation in Sub-Saharan Africa: a transient aspect of scale-up or a long-term phenomenon? J Health Care Poor Underserved 2013; 24: 359–83.
5. Rosen S, Maskew M, Fox MP, et al. Initiating antiretroviral therapy for HIV at a patient's first clinic visit: the RapIT randomized controlled trial. PLoS Med 2016; 13: e1002015.
6. Lessells RJ, Mutevedzi PC, Cooke GS. Retention in HIV care for individuals not yet eligible for antiretroviral therapy: rural KwaZulu-Natal , South Africa. J Acquir Immune Defic Syndr 2011; 56: 79–86.
7. Rosen S, Fox MP, Larson B, et al. Accelerating the uptake and timing of antiretroviral therapy initiation in sub-Saharan Africa: an operations research agenda. PLoS Med 2016; 13: e1002106.

For peer review only - http://bmjopen.bmj.com/site/about/guidelines.xhtml

8. World Health Organization. Guidelines for intensified tuberculosis case-finding and isoniazid preventive therapy for people living with HIV in resource-constrained settings. World Health Organization, Geneva, 2011.
9. Wajanga BM, Kalluvya S, Downs JA, Johnson WD, Fitzgerald DW, Peck RN. Universal screening of Tanzanian HIV-infected adult inpatients with the serum cryptococcal antigen to improve diagnosis and reduce mortality: An operational study. J Int AIDS Soc 2011; 14: 48.
10. Aoussi EF, Ehui E, Demb JP, et al. Cryptoccocal meningitis and HIV in the era of HAART in Cote d'Ivoire. Med Mal Infect 2012; 42: 349–54.
11. Grimes RM, Grimes DE. Readiness: The state of the science (or the lack thereof). Curr HIV/AIDS Rep 2010; 7: 245–52.
12. Rathbun RC, Farmer KC, Lockhart SM, Stephens JR. Validity of a stage of change instrument in assessing medication adherence in indigent patients with HIV infection. Ann Pharmacother. 2007;41: 208–215.

12. Appendices

Appendix 1. Pre-screening log
Appendix 2. Screening form
Appendix 3. Informed consent information sheet and consent form
Appendix 4. Case report form
Appendix 5. Referral letter
Appendix 6. Recruiting flier

For peer review only

For peer review only - http://bmjopen.bmj.com/site/about/guidelines.xhtml

SPIRIT

STANDARD PROTOCOL ITEMS: RECOMMENDATIONS FOR INTERVENTIONAL TRIALS

SPIRIT 2013 Checklist: Recommended items to address in a clinical trial protocol and related documents*

Section/item	Item No	Description	Addressed on page number
**Administrative information**			
Title	1	Descriptive title identifying the study design, population, interventions, and, if applicable, trial acronym	______________
Trial registration	2a	Trial identifier and registry name. If not yet registered, name of intended registry	______________
	2b	All items from the World Health Organization Trial Registration Data Set	______________
Protocol version	3	Date and version identifier	______________
Funding	4	Sources and types of financial, material, and other support	______________
Roles and responsibilities	5a	Names, affiliations, and roles of protocol contributors	______________
	5b	Name and contact information for the trial sponsor	______________
	5c	Role of study sponsor and funders, if any, in study design; collection, management, analysis, and interpretation of data; writing of the report; and the decision to submit the report for publication, including whether they will have ultimate authority over any of these activities	______________
	5d	Composition, roles, and responsibilities of the coordinating centre, steering committee, endpoint adjudication committee, data management team, and other individuals or groups overseeing the trial, if applicable (see Item 21a for data monitoring committee)	______________

For peer review only

Introduction

Background and rationale	6a	Description of research question and justification for undertaking the trial, including summary of relevant studies (published and unpublished) examining benefits and harms for each intervention	______________
	6b	Explanation for choice of comparators	______________
Objectives	7	Specific objectives or hypotheses	______________
Trial design	8	Description of trial design including type of trial (eg, parallel group, crossover, factorial, single group), allocation ratio, and framework (eg, superiority, equivalence, noninferiority, exploratory)	______________

Methods: Participants, interventions, and outcomes

Study setting	9	Description of study settings (eg, community clinic, academic hospital) and list of countries where data will be collected. Reference to where list of study sites can be obtained	______________
Eligibility criteria	10	Inclusion and exclusion criteria for participants. If applicable, eligibility criteria for study centres and individuals who will perform the interventions (eg, surgeons, psychotherapists)	______________
Interventions	11a	Interventions for each group with sufficient detail to allow replication, including how and when they will be administered	______________
	11b	Criteria for discontinuing or modifying allocated interventions for a given trial participant (eg, drug dose change in response to harms, participant request, or improving/worsening disease)	______________
	11c	Strategies to improve adherence to intervention protocols, and any procedures for monitoring adherence (eg, drug tablet return, laboratory tests)	______________
	11d	Relevant concomitant care and interventions that are permitted or prohibited during the trial	______________
Outcomes	12	Primary, secondary, and other outcomes, including the specific measurement variable (eg, systolic blood pressure), analysis metric (eg, change from baseline, final value, time to event), method of aggregation (eg, median, proportion), and time point for each outcome. Explanation of the clinical relevance of chosen efficacy and harm outcomes is strongly recommended	______________
Participant timeline	13	Time schedule of enrolment, interventions (including any run-ins and washouts), assessments, and visits for participants. A schematic diagram is highly recommended (see Figure)	______________

For peer review only - http://bmjopen.bmj.com/site/about/guidelines.xhtml

Sample size	14	Estimated number of participants needed to achieve study objectives and how it was determined, including clinical and statistical assumptions supporting any sample size calculations	______________
Recruitment	15	Strategies for achieving adequate participant enrolment to reach target sample size	______________

Methods: Assignment of interventions (for controlled trials)

Allocation:

Sequence generation	16a	Method of generating the allocation sequence (eg, computer-generated random numbers), and list of any factors for stratification. To reduce predictability of a random sequence, details of any planned restriction (eg, blocking) should be provided in a separate document that is unavailable to those who enrol participants or assign interventions	______________
Allocation concealment mechanism	16b	Mechanism of implementing the allocation sequence (eg, central telephone; sequentially numbered, opaque, sealed envelopes), describing any steps to conceal the sequence until interventions are assigned	______________
Implementation	16c	Who will generate the allocation sequence, who will enrol participants, and who will assign participants to interventions	______________
Blinding (masking)	17a	Who will be blinded after assignment to interventions (eg, trial participants, care providers, outcome assessors, data analysts), and how	______________
	17b	If blinded, circumstances under which unblinding is permissible, and procedure for revealing a participant's allocated intervention during the trial	______________

Methods: Data collection, management, and analysis

Data collection methods	18a	Plans for assessment and collection of outcome, baseline, and other trial data, including any related processes to promote data quality (eg, duplicate measurements, training of assessors) and a description of study instruments (eg, questionnaires, laboratory tests) along with their reliability and validity, if known. Reference to where data collection forms can be found, if not in the protocol	______________
	18b	Plans to promote participant retention and complete follow-up, including list of any outcome data to be collected for participants who discontinue or deviate from intervention protocols	______________

Data management	19	Plans for data entry, coding, security, and storage, including any related processes to promote data quality (eg, double data entry; range checks for data values). Reference to where details of data management procedures can be found, if not in the protocol	______
Statistical methods	20a	Statistical methods for analysing primary and secondary outcomes. Reference to where other details of the statistical analysis plan can be found, if not in the protocol	______
	20b	Methods for any additional analyses (eg, subgroup and adjusted analyses)	______
	20c	Definition of analysis population relating to protocol non-adherence (eg, as randomised analysis), and any statistical methods to handle missing data (eg, multiple imputation)	______
**Methods: Monitoring**			
Data monitoring	21a	Composition of data monitoring committee (DMC); summary of its role and reporting structure; statement of whether it is independent from the sponsor and competing interests; and reference to where further details about its charter can be found, if not in the protocol. Alternatively, an explanation of why a DMC is not needed	______
	21b	Description of any interim analyses and stopping guidelines, including who will have access to these interim results and make the final decision to terminate the trial	______
Harms	22	Plans for collecting, assessing, reporting, and managing solicited and spontaneously reported adverse events and other unintended effects of trial interventions or trial conduct	______
Auditing	23	Frequency and procedures for auditing trial conduct, if any, and whether the process will be independent from investigators and the sponsor	______
**Ethics and dissemination**			
Research ethics approval	24	Plans for seeking research ethics committee/institutional review board (REC/IRB) approval	______
Protocol amendments	25	Plans for communicating important protocol modifications (eg, changes to eligibility criteria, outcomes, analyses) to relevant parties (eg, investigators, REC/IRBs, trial participants, trial registries, journals, regulators)	______

For peer review only - http://bmjopen.bmj.com/site/about/guidelines.xhtml

Consent or assent	26a	Who will obtain informed consent or assent from potential trial participants or authorised surrogates, and how (see Item 32)	_____________
	26b	Additional consent provisions for collection and use of participant data and biological specimens in ancillary studies, if applicable	_____________
Confidentiality	27	How personal information about potential and enrolled participants will be collected, shared, and maintained in order to protect confidentiality before, during, and after the trial	_____________
Declaration of interests	28	Financial and other competing interests for principal investigators for the overall trial and each study site	_____________
Access to data	29	Statement of who will have access to the final trial dataset, and disclosure of contractual agreements that limit such access for investigators	_____________
Ancillary and post-trial care	30	Provisions, if any, for ancillary and post-trial care, and for compensation to those who suffer harm from trial participation	_____________
Dissemination policy	31a	Plans for investigators and sponsor to communicate trial results to participants, healthcare professionals, the public, and other relevant groups (eg, via publication, reporting in results databases, or other data sharing arrangements), including any publication restrictions	_____________
	31b	Authorship eligibility guidelines and any intended use of professional writers	_____________
	31c	Plans, if any, for granting public access to the full protocol, participant-level dataset, and statistical code	_____________
**Appendices**			
Informed consent materials	32	Model consent form and other related documentation given to participants and authorised surrogates	_____________
Biological specimens	33	Plans for collection, laboratory evaluation, and storage of biological specimens for genetic or molecular analysis in the current trial and for future use in ancillary studies, if applicable	_____________

*It is strongly recommended that this checklist be read in conjunction with the SPIRIT 2013 Explanation & Elaboration for important clarification on the items. Amendments to the protocol should be tracked and dated. The SPIRIT checklist is copyrighted by the SPIRIT Group under the Creative Commons "Attribution-NonCommercial-NoDerivs 3.0 Unported" license.

Simplified clinical algorithm for identifying patients eligible for immediate initiation of antiretroviral therapy for HIV (SLATE): protocol for a randomized evaluation

Journal:	*BMJ Open*
Manuscript ID	bmjopen-2017-016340.R1
Article Type:	Protocol
Date Submitted by the Author:	20-Mar-2017
Complete List of Authors:	Rosen, Sydney; Boston University School of Public Health, Center for Global Health and Development; University of the Witwatersrand Faculty of Health Sciences, Health Economics and Epidemiology Research Office<br>Fox, Matt; Boston University, Epidemiology and Global Health<br>Larson, Bruce; Boston University, School of Public Health<br>Brennan, Alana; Boston University<br>Maskew, Mhairi; University of the Witwatersrand, Health Economics and Epidemiology Research Office<br>Tsikhutsu, Isaac; Kenya Medical Research Institute, Walter Reed Projects<br>Bii, Margaret; Kenya Medical Research Institute, Walter Reed Projects<br>Ehrenkranz, Peter; Bill & Melinda Gates Foundation<br>Venter, WD Francois; University of the Witwatersrand, Wits RHI
**Primary Subject Heading**:	HIV/AIDS
Secondary Subject Heading:	Global health, Health services research
Keywords:	Antiretroviral therapy, Treatment initiation, South Africa, Kenya, Protocols & guidelines < HEALTH SERVICES ADMINISTRATION & MANAGEMENT, Randomized trial

For peer review only - http://bmjopen.bmj.com/site/about/guidelines.xhtml

Simplified clinical algorithm for identifying patients eligible for immediate initiation of antiretroviral therapy for HIV (SLATE): protocol for a randomized evaluation

Rosen S[1,2*], Fox MP[1,2,3], Larson BA[1], Brennan AT[1,2], Maskew M[2], Tsikhutsu I[4], Bii M[4], Ehrenkranz PD[5], Venter WDF[6]

[1]Department of Global Health, Boston University School of Public Health, Boston, Massachusetts, United States of America

[2]Health Economics and Epidemiology Research Office, Department of Internal Medicine, School of Clinical Medicine, Faculty of Health Sciences, University of the Witwatersrand, Johannesburg, South Africa

[3]Department of Epidemiology, Boston University School of Public Health, Boston, Massachusetts, United States of America

[4]Kenya Medical Research Institute/Walter Reed Project HIV Program, Kericho, Kenya

[5]Bill & Melinda Gates Foundation, Seattle, Washington, United States of America

[6]Wits Reproductive Health and HIV Institute, Department of Internal Medicine, School of Clinical Medicine, Faculty of Health Sciences, University of the Witwatersrand, Johannesburg, South Africa

*Author for correspondence: Sydney Rosen, Boston University School of Public Health, 801 Massachusetts Ave Room 390, Boston, MA 02118 USA, sbrosen@bu.edu, +1 617 414 1273.

Word count: 4,038

For peer review only

Abstract

Introduction: African countries are rapidly adopting guidelines to offer antiretroviral therapy (ART) to all HIV-infected individuals, regardless of CD4 count. For this policy of "treat all" to succeed, millions of new patients must be initiated on ART as efficiently as possible. Studies have documented high losses of treatment-eligible patients from care before they receive their first dose of antiretrovirals (ARVs), due in part to a cumbersome, resource-intensive process for treatment initiation, requiring multiple clinic visits over a several-week period.

Methods and analysis: The SLATE (Simplified Algorithm for Treatment Eligibility) study is an individually randomized evaluation of a simplified clinical algorithm for clinicians to reliably determine a patient's eligibility for immediate ART initiation without waiting for laboratory results or additional clinic visits. SLATE will enroll and randomize (1:1) 960 adult, HIV-positive patients who present for HIV testing or care and are not yet on ART in South Africa and Kenya. Patients randomized to the standard arm will receive routine, standard of care ART initiation from clinic staff. Patients randomized to the intervention arm will be administered a symptom report, medical history, brief physical exam, and readiness assessment. Patients who have positive (satisfactory) results for all four components of SLATE will be dispensed ARVs immediately, at the same clinic visit. Patients who have any negative results will be referred for further clinical investigation, counseling, tests, or other services prior to being dispensed ARVs. After the initial visit, follow up will be by passive medical record review. The primary outcomes will be ART initiation ≤28 days and retention in care 8 months after study enrollment.

Ethics and dissemination: Ethics approval has been provided by the Boston University Institutional Review Board, the University of the Witwatersrand Human Research Ethics Committee (Medical), and the KEMRI Scientific and Ethics Review Unit. Results will be published in peer-reviewed journals and made widely available through presentations and briefing documents.

Trial registration: Clinicaltrials.gov NCT02891135, registered September 1, 2016

For peer review only

Keywords: Antiretroviral therapy, Treatment initiation, Africa, South Africa, Kenya, Protocol, Randomized trial

Strengths and limitations of this study

- Evaluates a practical, simple algorithm for accelerating initiation of HIV treatment, a point at which many patients are currently lost from care
- Will contribute to the evidence base for achieving global targets
- Individually randomized trial conducted in six study sites in two African countries, making the results generalizable to the region
- Comparison arm is standard care, which could change over the course of the study

For peer review only

INTRODUCTION

In its 2015 revision of the global guidelines for HIV care and treatment, the World Health Organization (WHO) called for initiating lifelong antiretroviral treatment (ART) for all patients testing positive for HIV, rather than waiting for a patient's CD4 count to fall below 500 cells/mm^3, as in the previous guidelines.[1] Many African countries have adopted this recommendation and are rapidly developing new instructions and procedures for its implementation.

In its recommendation, which is referred to as "treat all" or "test and treat", the WHO cited "increases in ART uptake and linkage to care, reduction in the time between HIV diagnosis and ART initiation regardless of baseline CD4 cell count and an increase in the median CD4 value at ART initiation" as some of the main benefits to be gained from the guideline change, a conclusion recently confirmed in a modeling analysis.[2] For countries to benefit from treat all, therefore, millions of new patients must be initiated on ART as efficiently as possible, while ensuring that patient autonomy, welfare, and commitment to lifelong ART are not jeopardized by the initiation process. "Treat all," by removing the requirement of a pre-initiation CD4 count, promises to simplify and accelerate treatment initiation, if efficient procedures can be developed and implemented. Studies from throughout sub-Saharan Africa have documented high losses of treatment-eligible patients from care after they test positive for HIV but before they receive their first dose of antiretrovirals (ARVs), due to a wide range of facility- and patient-level barriers to initiation.[3][4] Multiple preparatory visits, long waiting times, stock outs of supplies, staff absences, and poor communication between staff and patients all deter treatment initiation.[5–8]

We previously proposed a simplified clinical algorithm for allowing nurses and other clinicians to reliably determine a patient's eligibility for immediate ART initiation without waiting for laboratory results or additional clinic visits.[9] The Simplified Algorithm for Treatment Eligibility (SLATE) includes a symptom report, medical history, brief physical exam, and readiness assessment; patients who "screen in" on all four screens can be dispensed ARVs on the spot, without any further steps, while those who do not are referred for further assessment, care, or support. An international technical consultation in 2015 regarded evaluating this algorithm as

For peer review only - http://bmjopen.bmj.com/site/about/guidelines.xhtml

the highest priority question on the operational research agenda it developed[9].

In this paper, we describe the protocol for a pragmatic, individually randomized evaluation of the effectiveness of the new algorithm in increasing ART uptake and retention in care in South Africa and Kenya. The SLATE study will evaluate whether patients who are administered the SLATE screens and either initiated on ART immediately or referred for further care have higher uptake of ART at 28 days and better retention in care at 6 months than do patients who receive standard ART initiation.

The purpose of the SLATE algorithm is to allow clinics to initiate ART in a way that minimizes the time required for both patients and staff and reduces loss to follow up prior to treatment initiation for the majority of patients who require no additional clinical care or psychosocial support before they can start. For those who would benefit from additional care or support—whether an additional counseling session, a TB test, or a more comprehensive physical examination—the algorithm is intended to identify and refer such patients for the additional care required, without slowing down the process for those who do not.

METHODS AND ANALYSIS

SLATE will be an individually randomized, non-blinded, pragmatic evaluation of the effect of the SLATE algorithm on ART initiation and retention in care, conducted at three public-sector clinics each in Kenya and South Africa.

Intervention: The SLATE algorithm and immediate ART initiation

The intervention to be evaluated in this study is a clinical algorithm that streamlines and structures the information required from patients before ARVs are dispensed for the first time. The algorithm consists of four "screens": symptom report, medical history, brief physical examination, and readiness assessment. Patients who "screen in"—have satisfactory responses to all four screens—can then be dispensed ARV medications on the spot, without any further steps or delays. Initiation of ART immediately after completing the four SLATE screens, without

For peer review only - http://bmjopen.bmj.com/site/about/guidelines.xhtml

any further services required, is labeled “immediate” initiation in this study. Patients who screen out—have at least one unsatisfactory response on a screen—are referred for further services, such as a laboratory test, more intensive physical examination, or counseling, before ARVs are dispensed.

Table 1 and Figure 1 describe the SLATE screens in detail, and the questions from the SLATE screening instruments to be used in the study are available in Supplementary File 1. Most of the information collected by the screens is consistent with current practice in study countries. The fourth screen, which aims to assess patient “readiness” to start ART, addresses an issue for which there is little standardization among countries or clinics. While emotional and psychological readiness to start ART is often regarded as important to achieving good medication adherence after initiation, a published review of readiness instruments for HIV initiation found that none was notably successful in predicting patient readiness for ART, as indicated by adherence once on treatment.[10] Primarily to ensure that patients have an opportunity to express any reservations they may have about starting ART and ask questions, we developed a three-question readiness assessment instrument.

Table 1. SLATE algorithm screens

Screen	Overall purpose of screen	Reasons for screening out	Justification	If screen out, anticipated next step
Symptom report	Identify self-reported conditions that require additional investigation	Current cough, fever, night sweats, or recent weight loss	These symptoms comprise the WHO-recommended tuberculosis symptom screen[11]	Referral for TB test
		Persistent headache for > 2 days	Symptom of cryptococcal meningitis[12,13]	Referral for CrAg screening
		Other self-reported symptoms	Other symptoms could indicate the need for further clinical investigation	Referral for additional clinical consultation
Medical history	Through self-report identify individuals on concurrent medications or who may struggle with adherence	On ART previously	Patients who have been on ART in the past may require additional adherence counseling	Referral for additional counseling session
		Started TB treatment within the past two weeks	Guidelines recommend a two-week delay in ART initiation for patients starting TB treatment	Appointment for ART initiation immediately after the two-week window has passed
		Concurrent medications for epilepsy or current warfarin	These medications can interact with ARVs	Referral for additional clinical or pharmacy consultation
		Current substance abuse	Use of recreational drugs or over-use of alcohol can create challenges for chronic medication adherence	Referral for additional counseling session
Physical examination	Record weight, height, temperature and blood pressure and identify any	Any conditions that call for further investigation	Patient may identify previously unreported symptoms or clinician may observe conditions that	Referral for additional clinical consultation

For peer review only - http://bmjopen.bmj.com/site/about/guidelines.xhtml

Screen	Overall purpose of screen	Reasons for screening out	Justification	If screen out, anticipated next step
	observable conditions that require additional investigation	prior to ART initiation	indicate a need for further clinical investigation before starting ART	
Readiness assessment	Confirm that the patient feels ready to start ART today	Responses that indicate reluctance, hesitation, or concerns in starting and adhering to treatment	Creates a structured opportunity for clinician and patient to discuss any concerns that the patient has not yet raised	Referral for additional counseling and follow-up support as indicated

Figure 1. Simplified algorithm for treatment eligibility (SLATE)

Outcomes, randomization, and sample size

The study has two primary outcomes, both based on the outcomes proposed for operational research on ART initiation in a previous publication[9]. For primary outcome 1, we will estimate the proportion of patients in each arm (baseline and intervention) who initiate ART within 28 days of study enrollment. Previous studies have found that 28 days is a sufficient time interval for a majority of patients found eligible for ART to complete the steps required to start treatment under routine care.[14,15] A patient who has not initiated within 28 days will be regarded as failing to achieve this primary outcome. We note that 28 days is a relatively generous interval to allow for achievement of this outcome, since the intervention is designed to allow ART initiation within one day (ie on the same day). From the perspective of a patient who is asymptomatic at first clinic visit and thus eligible for the SLATE algorithm patient's perspective, however, there appear to be few clinical benefits to starting within one day compared to 28 days. The real benefit of the SLATE approach is to eliminate the possibility of loss to follow up between the first visit and treatment initiation. By providing a relatively long interval for standard arm patients to start ART, we can be assured that any observed differences between the arms reflect true benefits to patients' health. Secondary outcomes, as described below, will also allow proportions of patients initiating ART at any interval after study enrollment to be assessed.

For primary outcome 2, we will estimate the proportion of patients in each arm who initiate ART within 28 days (i.e. achieve primary outcome 1) and are alive, in care, and retained on ART eight months after study enrollment. Eight months was selected to allow up to 1 month (28 days) to initiate ART, six months of follow up after treatment

For peer review only - http://bmjopen.bmj.com/site/about/guidelines.xhtml

initiation, and up to 1 month to return for the 6-month routine clinic visit. Although viral suppression is preferable to retention in care as a measure of ART success, we are not confident that all the study sites will consistently perform routine viral load tests at 6 months, and viral suppression will be considered as a secondary outcome. To allow for the irregularity of clinic visits, we will allow any clinic visit between 5 and 7 months after treatment initiation (or between 6 and 8 months after study enrollment, taking into account the month allowed for treatment initiation) to represent the 6-month visit. To help explain our results, we will also describe reasons for not achieving this primary outcome to the extent that this information is available in routine clinic records and will note any adverse events encountered during the eight-month follow-up period for this outcome.

Secondary outcomes are described in Table 2.

Table 2. Secondary outcomes

Secondary outcome	Justification and/or further description	Data analysis
**ART outcomes**		
ART initiation within 14 days of study enrollment	National guidelines in both study countries[16,17] recommend initiation ≤ 14 days	Intention to treat analysis; comparison of proportions between groups presented as a risk difference and 95% confidence intervals
Time to initiation, in days	One goal of SLATE algorithm is to accelerate initiation; time to initiation captures any effect on this.	Intention to treat analysis; comparison of time to initiation presented as survival curves with log rank test
Viral suppression by 8 months after study enrollment	Allows ≤ 1 month (28 days) to initiate ART, 6 months of follow up after treatment initiation, and ≤ 1 month to return for the 6-month routine clinic visit	Intention to treat analysis; comparison of proportions between groups presented as a risk difference and 95% confidence intervals. Reasons for not achieving this outcome will also be described to the extent that routinely collected follow up data allow.
Retention in care 14 months after study enrollment	Allows ≤ 1 month (28 days) to initiate ART, 12 months of follow up after treatment initiation, and ≤ 1 month to return for the 12-month routine clinic visit; any visit 12-14 months after study enrollment will represent the 12-month visit	Intention to treat analysis; comparison of proportions between groups presented as a risk difference and 95% confidence intervals
Retention in care at 16 months after study enrollment	Allows ≤ 1 month (28 days) to initiate ART, 12 months of follow up after treatment initiation, and ≤ 3 months to return for the 12-month routine clinic visit, to allow comparability to other studies of 12 month retention in care, which often define loss to follow-up as 90 days late for the last scheduled visit.	Intention to treat analysis; comparison of proportions between groups presented as a risk difference and 95% confidence intervals
**SLATE evaluation**		
Proportions of study patients who screen in and screen out for immediate ART initiation using SLATE algorithm criteria	Will provide guidance on proportions of patients who could be initiated under SLATE if adopted as routine care	Intention to treat analysis; comparison of proportions between groups presented as a risk difference and 95% confidence intervals
Reasons for ineligibility	Will provide guidance on types of referral services required from clinics	Descriptive analysis of proportions of patients screening out for each possible reason indicated on SLATE screens

For peer review only - http://bmjopen.bmj.com/site/about/guidelines.xhtml

Patient preferences on the speed and timing of ART initiation	Baseline questionnaire data	Descriptive analysis of medians and IQRs for continuous outcomes and proportions and corresponding 95% confidence intervals for categorical outcomes
**Health system outcomes**		
Costs to patients of ART initiation under standard and intervention procedures	SLATE is hypothesized to reduce the number of clinic visits required for ART initiation and thus costs to patients	Sum of clinic visit costs and time spent from enrollment visit to visit at which ARVs are dispensed, calculated from questionnaire responses.
Costs to providers of ART initiation under standard and intervention procedures and cost-effectiveness of intervention	SLATE is hypothesized to reduce the number of clinic visits required for ART initiation and thus costs to providers	Estimate of provider costs using previously described[18] bottom-up costing methods, with resource utilization extracted from medical records and CRFs and unit costs obtained from study sites. The average cost to the provider per patient achieving each primary outcome will be compared between intervention and standard initiation groups to provide an estimate of the cost-effectiveness of the two strategies. Costs will be reported as means (standard deviations) and medians (IQRs) in local currencies and USD dollars.

Study patients will be randomized 1:1, using block randomization in blocks of six, to the intervention arm or standard care arm. Randomization will be done at the country level, with an expected sample size of 160 subjects per site but with the possibility of allocating more or fewer slots to individual sites, while maintaining randomization rules, if enrollment rates differ. Blinding is not possible in a pragmatic evaluation such as SLATE, as each arm will follow very different procedures post-randomization.

The target sample size for the study, which was powered for the second primary outcome (initiated ART within 28 days and alive, in care, and retained on ART eight months after study enrollment), is 480 patients per country, or 960 in total. Using results of the RapIT randomized trial we recently conducted in South Africa,[14] we estimated that 65% of treatment-eligible patients will be initiated on antiretroviral therapy and retained on ART in the standard arm, and we considered an increase to 80% to be programmatically important. Using an α of 0.05, power of 90%, 1:1 randomization, and an uncorrected Fisher's exact test, this requires a minimum sample size of 197 patients per group, which we will increase to a maximum of 240 to ensure sufficient power, with stratification by country, if patients withdraw or are found ineligible after consent.

Study sites and ethics review

For peer review only - http://bmjopen.bmj.com/site/about/guidelines.xhtml
For peer review only

South Africa and Kenya, where the study will be conducted, differ widely in income and HIV prevalence, and thus will allow us to determine the effectiveness of the intervention in varying settings. In each country, patients will be enrolled at three public-sector clinics that have infrastructure and staff that are typical of the facilities that treat the vast majority of HIV patients. The only restriction we applied in selecting sites was that they have relatively high patient volumes, to speed up study enrollment. The sites are described in Table 3.

Table 3. Study sites

Country, implementer, and registry	Province/district or county	Site name	Patient population served
South Africa: Health Economics and Epidemiology Research Office (HE^{2}RO), University of the Witwatersrand, Johannesburg. South African National Health Research Database GP_2016RP42_318.	Gauteng Province, Johannesburg Metro	OR Tambo Primary Health Clinic	Urban informal settlement (Diepsloot)
	Gauteng Province, Johannesburg Metro	Alexandra Community Health Centre	Urban informal settlement (Alexandra)
	Gauteng Province, Ekhurhuleni Metro	Jabulani Dumani Community Health Centre	Urban informal settlement (Vosloorus)
Kenya: Kenya Medical Research Institute/Walter Reed Projects (KEMRI/WRP), Kericho. Pan African Clinical Trial Registry PACTR201609001783150.	Kericho County	Kericho County Referral Hospital	Kericho town and surrounding rural areas
	Kapsabet County	Kapsabet County Referral Hospital	Kabsabet town and surrounding rural areas
	Kombewa County	Kombewa County Hospital	Rural areas northwest of Kisumu

Study implementation in each country will be led by a local research organization subcontracted to the primary study implementer, Boston University (Table 3). At each site, the study will employ a study clinician in the same professional cadre that initiates patients onto ART under standard care. In South Africa, ART is typically initiated by public health nurses, who are the most senior cadre of nurse in that country; in Kenya, ART is initiated by clinical officers. The study clinicians will be trained on study procedures but have no additional clinical training or qualifications, beyond what is typical in routine clinic settings. Each site will also have one or two study assistants, who will be responsible for screening potential subjects, obtaining informed consent, administering the baseline questionnaire, and performing other non-clinical tasks. The study will have two dedicated rooms or private spaces at the sites, one for the nurse/clinical officer and one for the study assistant. Where necessary, we will renovate existing space or provide a separate, stand-alone space, such as a trailer, for study activities.

The study protocol, which is available as Supplementary File 2, has been approved by the Boston University Institutional Review Board and the University of the Witwatersrand Human Research Ethics Committee (Medical)

For peer review only - http://bmjopen.bmj.com/site/about/guidelines.xhtml

and is under review by the KEMRI Scientific and Ethics Review Unit. It is registered with Clinicaltrials.gov as NCT02891135.

Screening and enrollment

At each site, we will recruit 160 adult patients (≥18 years) who have tested positive for HIV, either at the current clinic visit or previously, and have not yet initiated ART. Pregnant women will be excluded, as procedures for initiating and managing pregnant women on ART differ from those for non-pregnant adults. Patients who intend to receive further HIV care at a different clinic, rather than the study site, will also be excluded, as will those who are determined by study staff to be physically, mentally, or emotionally unable to participate. We note that these study eligibility criteria will allow enrollment of patients at varying points in the HIV care cascade. Some will be enrolled a few moments after having their first positive HIV test, while others will have been attending pre-ART monitoring visits for several years. The study sample will thus reflect the full range of ART-eligible patients presenting at African clinics as treat-all guidelines are rolled out but will face heterogeneity in patient characteristics within each arm.

Site staff will refer patients with HIV to the study assistant for study screening and consent. Patients will be screened consecutively in the order in which they are referred by the site staff on a first-come, first-served basis, based on the availability of the study assistant(s). While some patients may prefer not to wait until the study assistant is available and opt out of being screened for enrollment, we do not anticipate that the screening process will introduce any biases to the sample ultimately enrolled. All patients who are found eligible for the study through the screening process will be asked for written informed consent and enrolled in the study. Following consent, female patients will be asked to complete a pregnancy test, and any who are found to be pregnant will be withdrawn from the study and escorted to the site's antenatal clinic to enroll in antenatal care and prevention of mother-to-child transmission (PMTCT) care.

One of the goals of implementation science studies is to generate evidence in a time frame that is

For peer review only - http://bmjopen.bmj.com/site/about/guidelines.xhtml

programmatically relevant. To accelerate reaching the country enrollment targets, unequal enrollment by site will be allowed, to take advantage of sites that have higher patient volumes. Enrollment in SLATE is expected to be completed within a 6-8 month period in 2017. The first primary outcome can then be estimated just 28 days after the last patient has been enrolled, while the second primary outcome will require 8 months of follow up. We thus expect most study results to be available by the end of 2018.

Procedures

Figure 2. Study flow diagram

Study procedures are illustrated in Figure 2. Prior to randomization, patients who provide written informed consent to study participation will be administered a baseline questionnaire by the study assistant, with sections on demographic and socioeconomic status, costs incurred per clinic visit, and preferences for the timing and speed of ART initiation. Randomization assignments, stored in sealed envelopes, will be opened and conveyed to participants by the study assistant, after completing the questionnaire. After randomization, patients assigned to the standard arm will have no further study activities. They will receive a payment equivalent in value to USD 5-15 to thank them for participating and compensate them for their time. Payment will be in the form of a shopping voucher that can be used at nearby grocery/general goods stores in South Africa and cash in Kenya. The study assistant will then escort standard arm patients back to their original place in the patient queue and complete a standard clinic visit, as they would have done in the absence of the study, and will follow the clinic's regular schedule of procedures and visits for ART initiation. They will have no further interaction with study staff after this point.

Patients assigned to the intervention arm will be introduced to the study nurse/clinical officer, who will administer the four SLATE screens. For the study, all four screens will be completed for all patients assigned to the intervention arm, so that we can obtain a complete data set for all patients. If SLATE were used in routine care, we expect that the screens would be administered sequentially, so that a negative response to the first screen (for

For peer review only - http://bmjopen.bmj.com/site/about/guidelines.xhtml

example, report of a symptom of TB) would allow a decision to refer the patient for additional care to be made without having to complete the second screen, and so on. Patients would progress to the next screen only after being found positive on the previous one, where “positive” refers to satisfactory responses to all items on the screen, while “negative” indicates a response that requires referral. We expect that for the vast majority of patients, each screen will take less than five minutes to administer. We would anticipate that in routine practice the algorithm will take 15-20 minutes for most patients.

Following administration of the four screens, intervention arm patients will have a blood draw to allow the study to collect baseline CD4 counts and for any pre-initiation laboratory tests called for under standard care. Blood samples will be sent for processing at the same laboratories used by the study sites. Although baseline CD4 counts are no longer used in either study country to establish ART eligibility, they continue to be performed for all patients initiating ART in both countries. Baseline CD4 count remains a strong indicator of early outcomes on ART and is therefore an important variable for the study analysis.

The results of the four screens will indicate whether a patient has either “screened in” or “screened out” of the SLATE algorithm. Findings in any of the four screens that suggest that further clinical investigation, counseling, laboratory tests, or other services are advisable prior to dispensing ARVs will cause a patient to screen out. Patients who screen out will be referred for the suggested follow-up services and escorted to the appropriate clinic location for immediate follow up at the same visit where possible. If the recommended follow-up service is not available immediately, a follow-up appointment will be made for the patient.

It is important to note that screening out of the SLATE algorithm will not necessarily preclude same-day ART initiation, either in the study or if the algorithm were used in routine care. Many patients who screen out will receive the additional service at the same clinic visit (e.g. an additional meeting with a counselor) and can still be prescribed ARVs before completing the visit. In fact, we expect that many patients who screen out of SLATE could indeed start ART on the same day, if the clinic has the capacity to provide the follow-up service that is needed and there is enough time left in the day for the patient to be served. SLATE is not intended to delay ART initiation for

For peer review only

For peer review only - http://bmjopen.bmj.com/site/about/guidelines.xhtml

those who screen out, but rather to accelerate the process for those who screen in.

For patients who “screen in”—have satisfactory responses to all four SLATE screens, do not require any additional services, and is eligible for immediate dispensing of ARVs—the study clinician will then have a brief conversation with the patient to confirm that the patient remains ready to start ART, understands what happens next in the study, and has no further questions or concerns. The clinician will then write a prescription for an initial supply of medications and either dispense the medications directly from the clinician’s room or escort the patient to the clinic pharmacy for immediate dispensing, as per the usual practice at each clinic. Patients in the intervention arm will then receive the same token of appreciation (shopping voucher or cash) as was provided earlier to patients in the standard arm.

Data collection and management

For the primary analysis, the two main sources of study data will be case report forms (CRF) and patients’ routine medical records. The CRF includes responses to the baseline questionnaire and all four SLATE screens. It also has sections for confirming a participant’s randomization assignment, CD4 count, medication dispensing, next appointment date, and/or referral for further care, as needed. It concludes with a follow-up form for recording study outcomes manually from paper files, in case these are not available electronically at every site.

The SLATE CRF will be programmed in REDCap Mobile (https://www.project-redcap.org/software/mobile-app/), housed at Boston University. REDCap (Research Electronic Data Capture) is a secure, web-based application designed to support data capture for research studies.[19] Data capture forms will be built in REDCap’s web application and then loaded securely onto tablet computers for direct entry by study staff on site. Using REDCap Mobile, data can be entered off line, then uploaded to the REDCap server as soon as an internet connection is available. The program will ensure a consistent study identification number for each participant, maintain data security, and allow data quality monitoring by the study data management team in near-real time. Tablets will be accessible only to the study staff at the specified site, and data that are uploaded to the server will automatically

For peer review only

be erased from the tablets, to reduce any chance of loss of confidentiality.

With the exception of the follow-up form, CRFs will be completed at the end of a patient's enrollment visit. All data for the follow-up period, until all primary and secondary outcomes are reached, will come from routinely collected medical records. All the study sites maintain an electronic medical record (EMR) database that we will access for most follow up data. In South Africa, sites use Tier.Net, the national HIV monitoring system.[20] In Kenya, sites use either IQCare (https://fgiqcare.codeplex.com/) or the Kenya version of Open MRS (https://wiki.openmrs.org/display/ke/KenyaEMR). Because the electronic records are not always complete or up to date, for example if a clinic is short-staffed and a data entry backlog develops, the CRF follow-up form will be completed where needed. To fill in missing fields in the EMR databases, we will also search patients' paper files and other clinic data sources (e.g. registers) as needed.

To allow linking of data between the CRF and medical records, a separate linking form, also in REDCap, will be completed for each patient immediately after consent. The linking form will contain the patient's name, date of birth, clinic file number(s), and national identification number. It will be stored separately from all other data collection forms, and used solely to select the right medical records (electronic and/or paper) for each study participant.

In addition to the two main sources of data described above, data from screening forms, which will be entered into a separate REDCap database, will be used to compare the enrolled sample with all patients screened for the study, to determine if the study sample is representative of the screened population and summarize reasons for screening out of the study. We will also collect aggregate, facility-level information on patient volume, resource utilization, and unit costs to answer our secondary research questions.

CRF data and other information collected in REDCap (screening forms, linking forms) will be uploaded to the REDCap server on a daily basis, using a cellular network uplink from the tablets. Following an initial period of daily quality review, a study manager will monitor data quality and completeness on a weekly basis. Queries about the

For peer review only - http://bmjopen.bmj.com/site/about/guidelines.xhtml

data will be sent to study coordinators for follow up and correction by site level staff, as needed. All study investigators (principal and co-investigators) will have access to the full study data set, which will be centrally managed by the overall study manager, who is also a co-investigator. The exception to this is the linking form containing individual identifiers, to which only the overall principal investigator, country principal investigators, and overall study manager will have access.

Data generated by the study (case report forms) will be made available in de-identified format following closure of the protocol in a publicly available repository, to be identified in papers published from the study. Data obtained from the study sites (routinely generated medical record data) will not be owned by the study and cannot be made publicly available by the authors.

Data analysis

The analytic approach will be by intention-to-treat: subjects will be analyzed according to the intervention they were allocated to receive, whether or not they adhered to the defined intervention. Outcomes for patients randomized to the intervention arm who screen out of immediate ART initiation by the SLATE algorithm will count towards the intervention arm. Analyses will be pooled across countries, but we will also stratify the analysis of primary outcomes by country.

The analysis will begin with a simple comparison of the two treatment groups with respect to baseline predictors of outcomes to detect any imbalances. We will then compare the proportion of patients achieving each dichotomous outcome by group and will calculate crude risk ratios and crude risk difference and their corresponding 95% confidence intervals. We will look for effect modification by important predictors of the outcome. The primary modifier is expected to be country, as standard of care for ART initiation differs between the two study countries. We will also look for differences in effects stratified by age, sex, baseline body mass index, and CD4 count, which are predictors of retention in care, and by any other important demographic, socioeconomic, and clinical predictors of outcomes identified, using baseline data including questionnaire

For peer review only - http://bmjopen.bmj.com/site/about/guidelines.xhtml

responses. Our analysis for effect modification will use a simple stratification of the primary analysis by the potential modifier and reporting crude risk differences and risk ratios and their corresponding 95% confidence intervals.

Analytic methods for secondary outcomes are described in Table 2.

Results of the study will be disseminated through papers reporting on primary and secondary outcomes and published in peer-reviewed journals. In addition, shorter presentations of study findings will be prepared for dissemination to the study sites and patients and for use by local policy makers and program managers. As we expect the results to be of particular relevance to HIV care and treatment guidelines committees both nationally and internationally, efforts will be made to ensure that these bodies have access to the findings.

Limitations

We anticipate that the SLATE study will have four main limitations. First, the number of study countries will be small, which could reduce generalizability if patient populations differ in characteristics that affect study outcomes. Second, in order to reach the target sample size in the desired timeframe, there will be heterogeneity in the population enrolled, with some patients who have spent years in pre-ART monitoring and others who were diagnosed with HIV on the day of study enrollment. Third, ART-eligible patients who visit the study clinics but are determined by study staff to be too emotionally distraught (e.g. by just having learned of their HIV diagnosis) or physically ill to be asked to participate in a study will be excluded, which may bias the study sample toward patients who are physically or emotionally healthier than the overall population. And fourth, as in most operational research studies, we will have little control over what happens in our baseline (non-intervention) arm. Standard of care continues to evolve rapidly in Africa. Guideline revisions are frequent, and clinicians at the study sites may learn from the intervention and make adjustments to routine care before the study is completed. Any changes that occur will be reported by study staff and taken into account in data analysis. We note that although

For peer review only

For peer review only - http://bmjopen.bmj.com/site/about/guidelines.xhtml

patients participating the study will receive a payment that will presumably not be offered to patients if the SLATE intervention were adopted into standard care, the payment will be offered to both study arms and should therefore not affect either study outcomes or the generalizability of findings.

DISCUSSION

Improving the efficiency of HIV service delivery—which encompasses both improving outcomes and reducing costs—is a high priority of national governments and international agencies tasked with implementing and paying for universal access to ART. Patients who have already volunteered for HIV testing but not yet started ART are a promising target for intervention, as they have already identified themselves and, by coming forward for testing, provided a time and place to intervene. Streamlining procedures for HIV initiation thus offers a relatively easy way to increase efficiency. The resources freed by such improvements can, in turn, be used to expand access to and quality of healthcare provision overall.

The SLATE algorithm that we will evaluate in this study has the potential to reduce the time and resources that both providers and patients must invest in ART initiation, while also diminishing the likelihood that patients will get lost from care between diagnosis and treatment initiation. If it can do so without jeopardizing outcomes after starting ART—such as retention in care during the first year, as will be monitored by the study—then SLATE will offer national HIV programs and providers a new and more efficient approach to an important component of HIV care. As other researchers have argued, rigorous implementation studies of feasible and pragmatic approaches to reducing losses to care are essential to achieving global targets.[21] The SLATE study aims to generate evidence for one new such approach.

Figure 1 legend

Figure 1 adapted from Rosen S, Fox MP, Larson B, Sow PS, Ehrenkranz PD, Venter F, Manabe Y, Kaplan J, for the Models for Accelerating Treatment Initiation (MATI) Technical Consultation. Accelerating the uptake and timing of antiretroviral therapy initiation in sub-Saharan Africa: an operations research agenda. *PLoS Med* 2016; 13: e1002106. DOI:10.1371/journal.pmed.1002106 (CC BY 4.0).

For peer review only

Competing interests:

PDE is an employee of the Bill & Melinda Gates Foundation, which is funding this work. WDFV sits on antiretroviral initiation guideline committees, both local and international. WDFV has accepted speaking honoraria from multiple manufacturers of antiretrovirals and is on several of their advisory boards. The remaining authors declare that they have no competing interests.

Funding:

Funding for the work presented here was provided by the Bill & Melinda Gates Foundation under the terms of OPP1136158 to Boston University. The funders participated in the Technical Consultation reported here and are co-authors of the manuscript. The funders had no separate role, beyond that of other participants and authors, in study design, data collection and analysis, decision to publish, or preparation of the manuscript.

Authors' contributions:

Conceived of and designed the work: SR, MPF, BAL, PDE, WDFV. Contributed to designing the work: ATB, MM, IT, MB. Wrote the first draft of the manuscript: SR. Reviewed and revised the manuscript: all authors. Approve the manuscript's results and conclusions: all authors. All authors have read, and confirm that they meet, ICMJE criteria for authorship.

References

1. World Health Organization. Guideline on when to start antiretroviral therapy and on pre-exposure prophylaxis for HIV. Geneva: World Health Organization, 2015.
2. Barnabas RV, Bendavid E, Bershteyn A, Boulle A, Eaton JW, Ford N, et al. HIV mortality by care cas- cade stage and implications for universal ART eligibility. Conference on Retroviruses and Opportunistic Infections 2016; 22–25 Feb 2016; Boston. Available: http://www.croiconference. org/sessions/hiv-mortality-care-cascade-stage-and-implications-universal-art-eligibility.
3. Lahuerta M, Ue F, Hoffman S, Elul B, Kulkarni SG, Wu Y, et al. The problem of late ART initiation in Sub-Saharan Africa: a transient aspect of scale-up or a long-term phenomenon? J. Health Care Poor Underserved 2013;24:359–83.
4. Iwuji CC, Orne-Gliemann J, Larmarange J, Okesola N, Tanser F, Thiebaut R, et al. Uptake of home-based HIV testing, linkage to care, and community attitudes about ART in rural KwaZulu-Natal, South Africa: Descriptive results from the first phase of the ANRS 12249 TasP cluster-randomised trial. PLOS Med 2016;13:e1002107.
5. Govindasamy D, Ford N, Kranzer K. Risk factors, barriers and facilitators for linkage to ART care in sub-Saharan Africa: a systematic review. AIDS 2012;26:2059–67.
6. Micek M a, Gimbel-Sherr K, Baptista AJ, Matediana E, Montoya P, Pfeiffer J, et al. Loss to follow-up of adults in public HIV care systems in central Mozambique: identifying obstacles to treatment. J Acquir Immune Defic Syndr 2009;52:397–405.
7. Scott V, Zweigenthal V, Jennings K. Between HIV diagnosis and initiation of antiretroviral therapy: assessing the effectiveness of care for people living with HIV in the public primary care service in Cape Town, South Africa. Trop Med Int Heal 2011;16:1384–91.
8. Siedner MJ, Lankowski A, Haberer JE, Kembabazi A, Emenyonu N, Tsai AC, et al. Rethinking the "'pre'" in pre-therapy counseling: no benefit of additional visits prior to therapy on adherence or viremia in Ugandans initiating ARVs. PLoS One 2012;7:e39894.
9. Rosen S, Fox MP, Larson B, Sow PS, Ehrenkranz PD, Venter F, et al. Accelerating the uptake and timing of antiretroviral therapy initiation in sub-Saharan Africa: an operations research agenda. PLoS Med. 2016;13:e1002106.
10. Grimes RM, Grimes DE. Readiness: The state of the science (or the lack thereof). Curr HIV/AIDS Rep 2010;7:245–52.
11. World Health Organization. Guidelines for intensified tuberculosis case-finding and isoniazid preventive therapy for people living with HIV in resource-constrained settings. Geneva: World Health Organization, 2011.

For peer review only - http://bmjopen.bmj.com/site/about/guidelines.xhtml

12. Wajanga BM, Kalluvya S, Downs JA, Johnson WD, Fitzgerald DW, Peck RN. Universal screening of Tanzanian HIV-infected adult inpatients with the serum cryptococcal antigen to improve diagnosis and reduce mortality: An operational study. J Int AIDS Soc 2011;14:48.
13. Aoussi EF, Ehui E, Demb JP, Kolia-Diafouka P, Elloh NF, Ouattara SI, et al. Cryptoccocal meningitis and HIV in the era of HAART in Cote d'Ivoire. Med Mal Infect 2012;42:349–54.
14. Rosen S, Maskew M, Fox MP, Nyoni D, Mongwenyana C, Malete G, et al. Initiating antiretroviral therapy for HIV at a patient's first clinic visit: the RapIT randomized controlled trial. PLoS Med. 2016;13:e1002015.
15. Amanyire G, Semitala FC, Namusobya J, Katuramu R, Kampiire L, Wallenta J, et al. Effects of a multicomponent intervention to streamline initiation of antiretroviral therapy in Africa: a stepped-wedge cluster-randomised trial. Lancet HIV 2016; 3:e539-e548.
16. Ministry of Health National AIDS & STI Control Programme. Guidelines on use of antiretroviral drugs for treating and preventing HIVinfection in Kenya 2016. Nairobi; Ministry of Health, 2016.
17. Pillay Y. Implementation of the universal test and treat strategy for HIV positive patients and differentiated care for stable patients. Pretoria: Department of Health, 2016.
18. Long L, Brennan A, Fox MP, Ndibongo B, Jaffray IA, Sanne I, et al. Treatment outcomes and cost-effectiveness of shifting management of stable ART patients to nurses in South Africa: an observational cohort. PLoS Med 2011;8:e1001055.
19. Harris PA, Taylor R, Thielke R, Payne J, Gonzalez N, Conde JG. Research electronic data capture (REDCap)—A metadata-driven methodology and workflow process for providing translational research informatics support. J Biomed Inform 2009;42:377–81.
20. Osler M, Hilderbrand K, Hennessey C, Arendse J, Goemaere E, Ford N, et al. A three-tier framework for monitoring antiretroviral therapy in high HIV burden settings. J Int AIDS Soc 2014;17: 18908
21. Labhardt ND, Ringera I, Lejone TI, Masethothi P, Thaanyane T, Kamele M, et al. Same day ART initiation versus clinic-based pre-ART assessment and counselling for individuals newly tested HIV-positive during community-based HIV testing in rural Lesotho – a randomized controlled trial (CASCADE trial). BMC Public Health 2016;16:329.

For peer review only - http://bmjopen.bmj.com/site/about/guidelines.xhtml

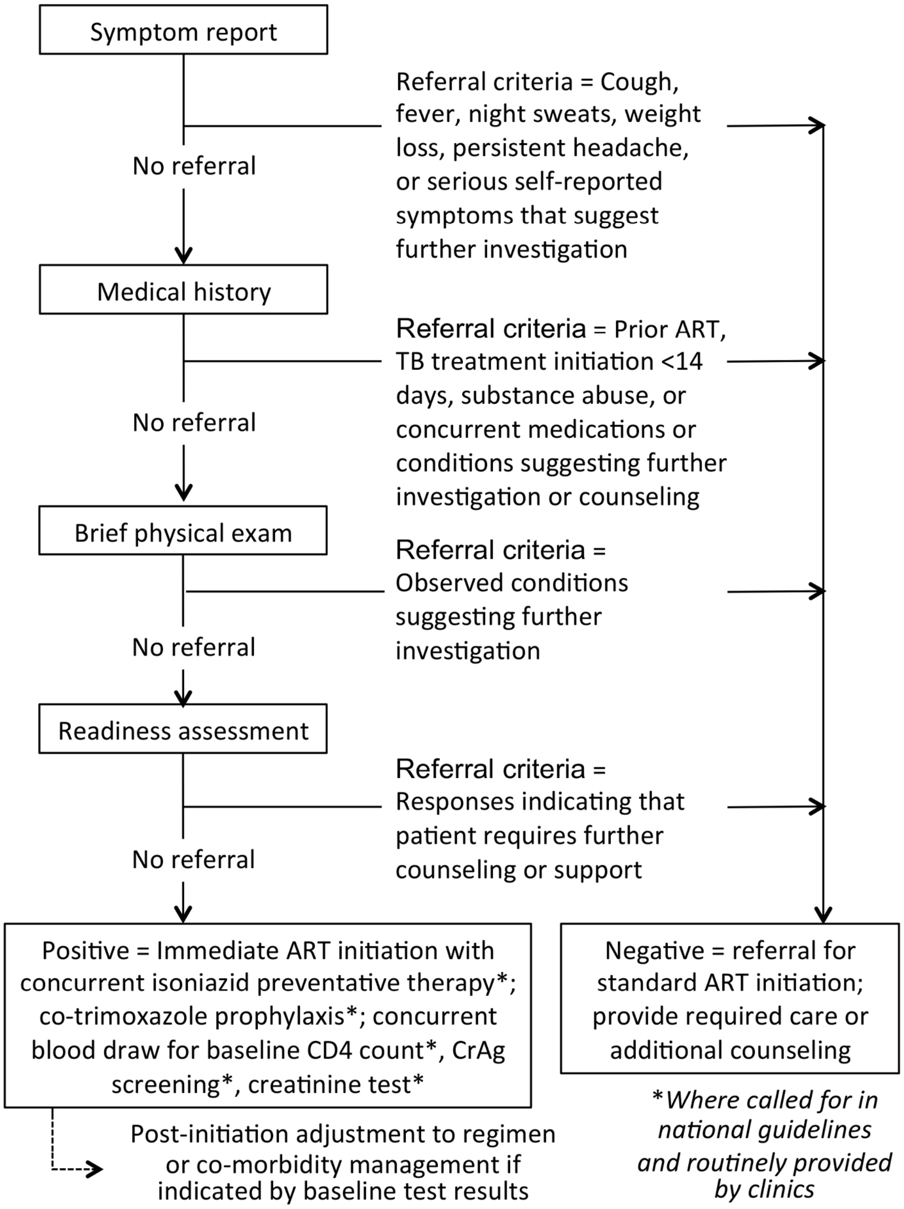

158x212mm (300 x 300 DPI)

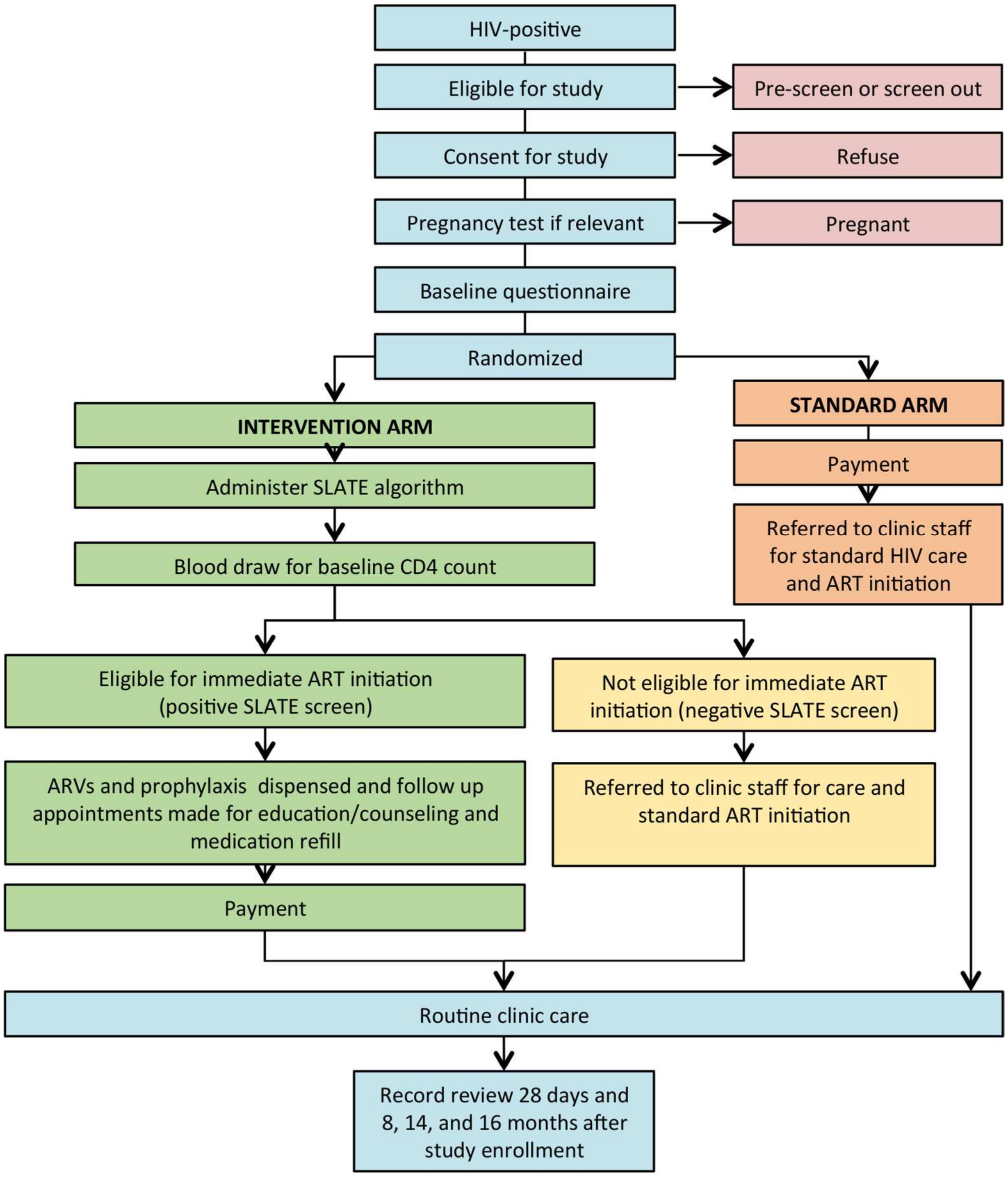

154x180mm (300 x 300 DPI)

SIMPLIFIED ALGORITHM FOR TREATMENT ELIGIBILITY (SLATE): SCREENS

Symptom report

1. Does patient have a cough? Yes/No
2. Does patient have a fever? Yes/No
3. Does patient report having night sweats? Yes/No
4. Does patient report losing weight? Yes/No
5. Does patient have a continuous headache that hasn't gone away for at least two days? (If headache is persistent or continuous, answer "yes." If occasional or temporary, answer "no.") Yes/No
6. Does patient report any other symptoms of illness? Yes/No

   If yes, what other symptoms? (Specify)

   If yes, do any of the other symptoms indicate that further consultation is needed before starting ART? Yes/No
7. On the basis of symptoms, should this patient be referred for further care before starting ART? Yes/No

Medical history

1. Have you been on ART before? Yes/No

   If yes, when did you start taking ART before? (Record)

   If yes, when did you stop taking ART before? (Record)
2. Are you currently on TB treatment? Yes/No

   If yes, how many weeks ago did you start taking TB treatment? (Record)

   If patient started taking TB treatment 1 or 2 weeks ago, how many days ago did she or he start TB treatment? (Record)
3. Have you been told that you have other diseases or health problems besides HIV? Yes/No

   If yes, what other diseases or conditions do you have? (Specify)

   If yes, do any of the diseases or conditions suggest further consultation is needed? Yes/No
4. Are you currently taking any anti-epilepsy medication? Yes/No
5. Are you currently taking warfarin? Yes/No
6. Are you currently taking any other medications? Yes/No

   If yes, what other medications are you currently taking? (Specify)

   If yes, do any of the medications suggest that further consultation is needed? Yes/No
7. Do you currently take any recreational drugs, such as smoking (bhang/ dagga/ weed) or using pills or injectables? Yes/No

For peer review only
NOT FOR USE IN PRACTICE

8. Do you currently drink more alcohol than you think you should? Yes/No
9. On the basis of medical history, should this patient be referred for further care before starting ART? Yes/No

Physical examination

1. Temperature (Record)
2. Systolic blood pressure (Record)
3. Diastolic blood pressure (Record)
4. Weight (kg) (Record)
5. Did the patient report symptoms that should be examined? Yes/No

   If yes, after examination, do any symptoms suggest that further consultation is needed? Yes/No

   If yes, which symptoms should be investigated further? (Specify)
6. Does the patient have other conditions for which further consultation is needed? Yes/No

   If yes, what are the other conditions? (Specify)
8. On the basis of the physical exam, should should this patient be referred for further care before starting ART? Yes/No

Readiness assessment

1. If you should start ART today, how ready are you?
   Not ready
   Thinking about trying, maybe in the next month or so
   Almost ready, maybe this week
   Ready today
2. How will you remember to take your medication every day?
   Mobile phone reminder
   Alarm on a clock or watch
   Ask someone to remind me
   Use a calendar or diary
   Take my tablets at the same time as I do something else every day (like brushing my teeth)
   No reminders, I'll just remember
   Other way (specify)
3. Is there anything that will stop you from taking your tablets every day? Yes/No
4. Did the patient raise any issues or concerns that lead you to think that ART should be delayed?

   If yes, what were these issues or concerns and how did you respond?
5. On the basis of the readiness assessment, should this patient be referred for further care before starting ART? Yes/No

For peer review only - http://bmjopen.bmj.com/site/about/guidelines.xhtml

For peer review only

NOT FOR USE IN PRACTICE

SLATE

Research Protocol

Randomized Evaluation of a Simplified Clinical Algorithm for Identifying Patients Eligible for Immediate Initiation of Antiretroviral Therapy for HIV (SIMPLIFIED ALGORITHM FOR TREATMENT ELIGIBILITY —THE SLATE STUDY)

BUMC IRB Protocol Number: H-35634 (United States)

Wits University HREC Protocol Number: 160910 (South Africa)

KEMRI SERU Protocol Number: XXX (Kenya)

WRAIR IRB Protocol Number: XXX (United States)

Federal-Wide Assurance Numbers:

FWA00000301 (Boston University Medical Campus)
FWA00000715 (University of the Witwatersrand)
FWA 00002066 (Kenya Medical Research Unit)
FWA00000015 (Walter Reed Army Institute of Research)

January 13, 2017

Version 1.1

For peer review only

Table of Contents

1. Summary **4**
2. Investigators **4**
3. Background **5**
3a. Context: New guidelines for HIV treatment eligibility 5
3b. The problem: loss to follow up before treatment initiation 6
3c. Proposed solution: a standardized clinical algorithm for determining eligibility for immediate treatment initiation 7
4. Objectives **9**
4a. Primary objectives 9
4b. Secondary objectives 9
5. Study Design **10**
5a. Overview 10
5b. Study sites 11
5c. Study population 12
6. Enrollment and Preparation **13**
6a. Pre-screening 13
6b. Screening 13
6c. Consent 14
6d. Pregnancy test 14
6e. Questionnaire 14
6f. Randomization 14
6g. Payment 14
7. Intervention **15**
7a. Algorithm data collection 15
7b. Blood draw 16
7c. Referral of patients with positive screens 16
7d. Dispensing of ARVs and prophylaxis 16
7e. Completion of direct study interaction 17
8. Medical Record Follow Up **17**
9. Data Collection and Management **18**
9a. Sources of data 18
9b. Pre-screening logbook 18
9c. Screening form 18
9d. Case report form 18
9e. Medical record data 19
9f. Study identification numbers and linking of records 21
9g. Data entry and storage 21
10. Staff Supervision and Training **22**
11. Sample Size **22**
12. Outcomes and Data Analysis **22**

For peer review only - http://bmjopen.bmj.com/site/about/guidelines.xhtml

12a. Outcomes 22
12b. Analytic methods 23
12c. Dissemination of findings 24
13. Human Subjects Considerations........ 25
13a. Risks and protection against risks 25
13b. Benefits 26
13c. Costs and payments to subjects 27
13d. Recruitment 27
13e. Informed consent 27
13f. Protection of confidentiality 28

14. References 28

12. Appendices 29

For peer review only

1. Summary

In its 2015 revision of the global guidelines for HIV care and treatment, the World Health Organization called for initiating lifelong antiretroviral treatment (ART) for all patients testing positive for HIV, regardless of CD4 cell count. As countries adopt the new recommendation, known as "treat all," millions of additional patients are becoming eligible for ART worldwide. In sub-Saharan Africa, where most of these patients are located, studies continue to document high losses of treatment-eligible patients from care before they receive their first dose of antiretroviral medications (ARVs). Among facility-level reasons for these losses are treatment initiation protocols that require multiple clinic visits and long waiting times before a patient who tests positive for HIV is dispensed an initial supply of medications. Simpler, more efficient, accelerated algorithms for ART initiation will be needed if "treat all" is to realize the benefits expected.

At a technical consultation convened in October 2015, participants proposed a simplified clinical algorithm to screen patients for eligibility for immediate ART initiation at a patient's first clinic visit, without the use of point-of-care laboratory test technologies. The Simplified Algorithm for Treatment Eligibility (SLATE) uses four screens to assess whether a patient is eligible for immediate (same-day) treatment initiation: i) symptom report, ii) medical history, iii) readiness assessment, and iv) brief physical examination. SLATE is a pragmatic, individually randomized evaluation to determine the effectiveness of the algorithm in increasing ART initiation, compared to standard care, among non-pregnant adult patients. A total of approximately 960 HIV-infected adult patients not yet on ART will be enrolled during a routine clinic visit and randomized to receive the intervention or standard care. Patients in the intervention arm will be administered the SLATE screens; those found eligible under the algorithm will be offered immediate treatment initiation, while those who are not eligible will be referred for standard clinic care. Patients in the standard arm will be referred for ART initiation under standard clinic procedures. All care after the initial visit will be by the clinic under standard of care. The study will be conducted at three healthcare facilities (clinics) in each of two countries, South Africa and Kenya. If successful, SLATE will offer a standardized approach to collecting and interpreting a minimum set of patient data that will avoid delaying treatment initiation for the majority of patients who are eligible for immediate ART, while deferring initiation in the minority who should not start immediately.

2. Investigators

Sydney Rosen
Research Professor
Boston University School of Public Health
Boston, MA, USA
Role on study: principal investigator

Matthew Fox
Associate Professor
Boston University School of Public Health
Boston, MA, USA
Role on study: co-investigator, statistical advisor

Bruce Larson
Research Professor
Boston University School of Public Health

For peer review only - http://bmjopen.bmj.com/site/about/guidelines.xhtml

Boston, MA, USA
Role on study: co-investigator, evaluation advisor

Alana Brennan
Research Scientist
Boston University School of Public Health
Boston, MA, USA
Role on study: co-investigator, study manager

Francois Venter
Associate Professor
Department of Medicine
Wits University
Johannesburg, South Africa
Role on study: co-investigator, clinical advisor

Mhairi Maskew
Senior Researcher
Health Economics and Epidemiology Research Office
University of the Witwatersrand
Johannesburg, South Africa
Role on study: South Africa PI, data analyst

Isaac Tsikhutsu
Medical Doctor
Kenya Medical Research Institute/Walter Reed Project HIV Program
Kericho, Kenya
Role on study: Kenya PI

3. Background

3a. Context: New guidelines for HIV treatment eligibility

In its 2015 revision of the global guidelines for HIV care and treatment, the World Health Organization called for initiating lifelong antiretroviral treatment (ART) for all patients testing positive for HIV, regardless of CD4 cell count[1]. This revision followed a series of increases in the global guidelines in the threshold for initiating ART, from a CD4 count of ≤200 cells/mm^3 prior to 2010, to 350 until 2013, to 500 at present. In its recommendation, the WHO cites three anticipated benefits from this approach, which is referred to as “treat all” (alternatively, “test and treat” or “test and start”): reduced morbidity among HIV-infected patients; reduced risk of transmission from HIV-infected individuals to their partners; and “*increases in ART uptake and linkage to care, reduction in the time between HIV diagnosis and ART initiation regardless of baseline CD4 cell count and an increase in the median CD4 value at ART initiation*.” A recent analysis concluded that future reductions in HIV-related mortality in sub-Saharan Africa will derive largely from this last benefit, rather than from the first two[2].

Multiple African countries—including South Africa and Kenya, where the proposed study will take place—have already announced their plans to adopt the new guidelines, and it is clear that the trend globally is toward offering ART to all. As this happens, national HIV programs will face the challenge of

For peer review only - http://bmjopen.bmj.com/site/about/guidelines.xhtml

initiating individuals newly diagnosed as HIV-infected on ART as efficiently as possible, while ensuring that retention on ART and patient welfare are not jeopardized by the initiation process.

3b. The problem: loss to follow up before treatment initiation

Studies from throughout sub-Saharan African continue to document high losses of treatment-eligible patients from care before they receive their first dose of ARVs. A systematic review of the literature on pre-ART retention in care found that roughly one third of adult patients in sub-Saharan Africa who had already been determined eligible for ART did not start ART within the studies' follow-up periods[3]. If those diagnosed with HIV but not assessed for treatment eligibility had been added, this proportion would have been far higher. Reasons for this include a wide range of facility- and patient-level barriers to initiation[4]. Among facility-level barriers are clinic inaccessibility and inefficiency and initiation protocols designed for the convenience of providers rather than patients. These barriers manifest themselves as multiple required visits, long waiting times, stock outs of supplies, staff absences, and poor communication between staff and patients. Eliminating the eligibility threshold under a "treat all" policy is not likely to solve these problems, as clinics become even more crowded with newly-eligible patients. Simpler, more efficient, accelerated algorithms for ART initiation will be needed if "treat all" is to realize the benefits expected.

In an earlier clinical trial in South Africa (RapIT, H-31880), we demonstrated that same-day treatment initiation, in which all the steps required for ART initiation are performed in sequence during a single clinic visit, significantly increased the proportion of eligible patients started on treatment (RR 1.36, 95% CI 1.24–1.49) and the proportion virally suppressed six months later (RR 1.26, 95% CI 1.05–1.50).[5] The RapIT trial was premised on the notion that standard of care treatment initiation, which in South Africa required 5 or 6 clinic visits over a 1-2 month period, created a burden on patients that deterred many from completing the process. In addition to delaying the start of treatment, each clinic visit entailed transport costs and time, many hours of waiting time in the clinic, and possibly lost wages, child care, and other costs for the patient[6]. The results of the study indicate that accelerating the initiation process does overcome these barriers; in the intervention arm of the study, 75% of study subjects started treatment on the same day as study enrollment, and nearly all the rest within one month. The study demonstrated that it is possible to initiate nearly 100% of eligible patients, and eliminate loss to follow up at this point in the care cascade.

Although RapIT and other studies have shown that the vast majority of patients presenting for HIV care at clinics in Africa can safely start ART on the same day, without delay, there are still patients for whom a delay is necessary. These reasons include:

- Tuberculosis (TB): To minimize the risk of immune reconstitution inflammatory syndrome (IRIS), two weeks of TB treatment is recommended for patients presenting with active TB before ART is started.
- Cryptococcal meningitis (CM): Patients with low CD4 counts should be screened for cryptococcal antigen (CrAg) and given presumptive treatment for CM before ART is started, again to minimize the risk of IRIS.
- Other clinical conditions: HIV patients present with a range of symptoms and conditions that may lead a clinician to decide to delay ART while further investigations or treatment are completed.
- Patient history: Patients who previously defaulted from ART or have a history predicting non-adherence, such as substance abuse, may require additional support before starting ART.
- Patient readiness: Finally, some patients may feel that they are not ready to start treatment on the same day and choose for themselves to delay initiation.

For peer review only - http://bmjopen.bmj.com/site/about/guidelines.xhtml

The RapIT trial used multiple rapid, point-of-care laboratory test instruments to generate the laboratory data required for ART initiation in South Africa. The generalizability of its findings is thus limited by the need for expensive equipment that will not be feasible to maintain in most primary healthcare clinics in the region. RapIT also excluded many ART-eligible patients on the basis of clinical trial-specific exclusion criteria that would not apply in a routine care setting. What is needed now is a way to assess eligibility for immediate treatment initiation that is broadly applicable to nearly all patients and does not require either point-of-care laboratory instruments or delays for laboratory results to be received, while screening out patients who should not start treatment immediately for clinical or personal reasons.

3c. Proposed solution: a standardized clinical algorithm for determining eligibility for immediate treatment initiation

In October 2015, the investigators convened a technical consultation of experts in the field to discuss models for accelerating treatment initiation[7]. Participants in that meeting proposed a simplified clinical algorithm to screen patients for eligibility for immediate ART initiation without the use of point-of-care technologies. The simplified algorithm, abbreviated “SLATE,” is illustrated in Figure 1.

For peer review only

Figure 1. SLATE algorithm

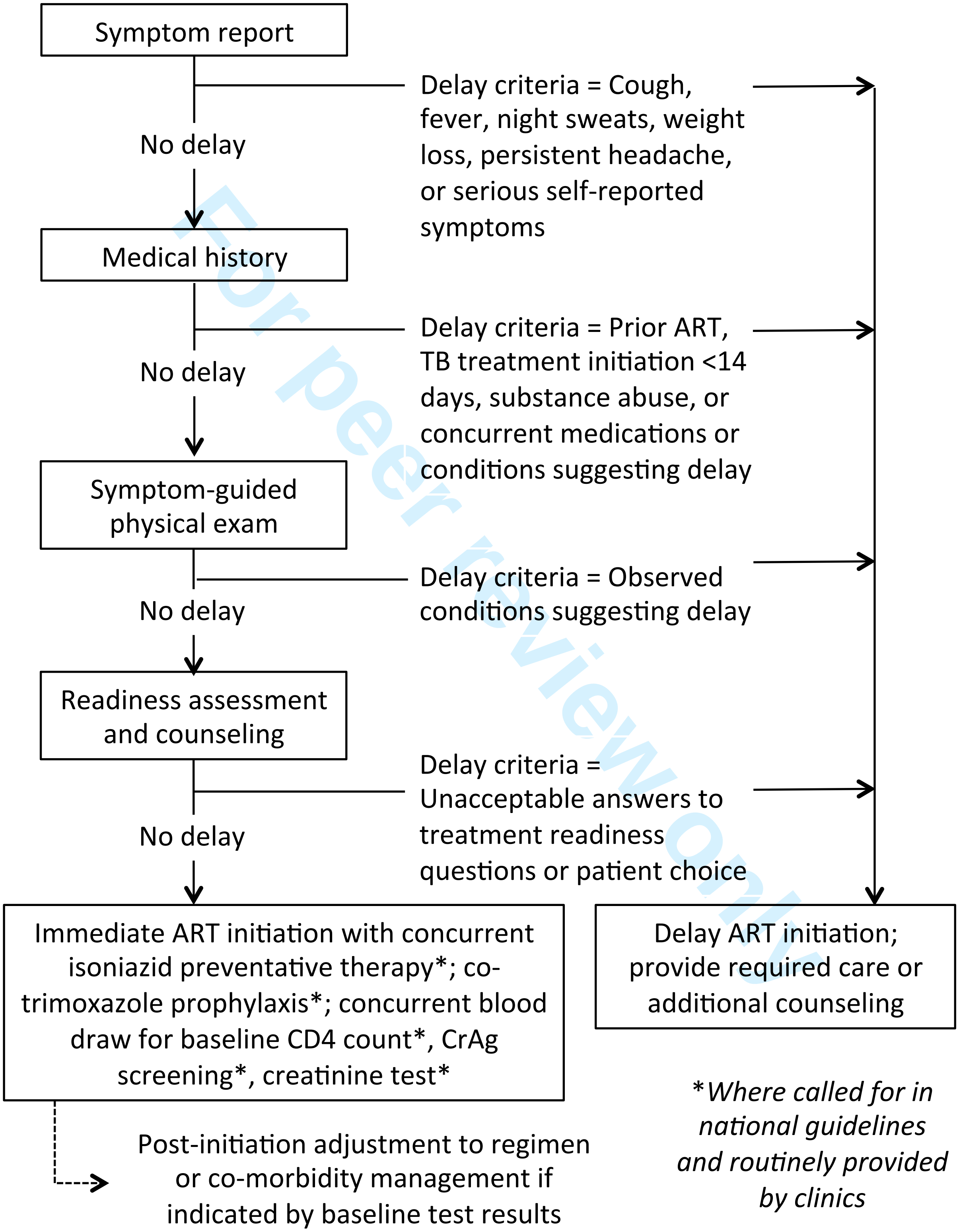

For peer review only

For peer review only - http://bmjopen.bmj.com/site/about/guidelines.xhtml

As shown in Figure 1, the SLATE algorithm uses four screens to assess whether a patient is eligible for immediate (same-day) treatment initiation. Each of the screens is intended to identify specific reasons that a patient might *not* be eligible, including current symptoms of opportunistic infections, previous experiences or behaviors that indicate a need for additional treatment readiness or adherence support, and the patient's own concerns about starting treatment. The algorithm captures all components of treatment eligibility screening currently used in routine care in the study countries with the exception of pre-initiation laboratory results. It follows national guidelines for detection of co-morbidities, which vary by country; some countries call for a CrAg test for patients with CD4 counts < 100, for example, while others do not conduct routine CrAg screening.

The purpose of the SLATE algorithm is to create a standardized approach to collecting and interpreting a minimum set of patient data that will avoid delaying treatment initiation for the majority of patients who are eligible for immediate ART, while deferring initiation in the minority who should not start immediately. If successful, it is expected to increase overall uptake of ART and viral suppression in the population of HIV-infected adults eligible for ART in sub-Saharan Africa.

4. Objectives

The SLATE study is a pragmatic, individually randomized evaluation to determine the effectiveness of the SLATE algorithm in increasing ART initiation, compared to standard care, among non-pregnant adult patients. Patients will be individually randomized to either an intervention arm, in which the SLATE algorithm will be implemented, or a standard arm, in which they will receive standard treatment initiation.

4a. Primary objectives

The primary objectives of this study are to:

1. Compare the proportion of study-eligible patients who initiate ART within 28 days of study enrollment between HIV-infected patients offered immediate ART initiation under the SLATE algorithm and patients offered standard ART initiation.

2. Compare the proportion of study-eligible patients who initiate ART and are alive, in care, and retained on ART by eight months after study enrollment between HIV-infected patients offered immediate ART initiation under the SLATE algorithm and patients offered standard ART initiation.

4b. Secondary objectives

The secondary objectives of this study are to:

3. Compare the proportion of study-eligible patients who initiate ART within 14 days of study enrollment between HIV-infected patients offered immediate ART initiation under the SLATE algorithm and patients offered standard ART initiation.
4. At sites where viral load data are available, compare the proportion of study-eligible patients who initiate ART and are virally suppressed eight months after having an HIV test or enrolling in HIV care
5. Compare the proportion of study-eligible patients who initiate ART and are alive, in care, and retained on ART by 14 months and 16 months after study enrollment between HIV-infected patients

For peer review only - http://bmjopen.bmj.com/site/about/guidelines.xhtml

For peer review only

offered immediate ART initiation under the SLATE algorithm and patients offered standard ART initiation.

6. Estimate the proportion of HIV-positive patients presenting at study clinics and not yet on ART who are eligible or ineligible for immediate initiation using SLATE algorithm criteria.
7. Describe reasons for ineligibility for immediate initiation, among those found ineligible in the intervention arm.
8. Estimate average time interval in days between study enrollment and ART initiation for each study arm.
9. Describe self-reported patient preferences on the speed and timing of ART initiation.
10. Estimate costs to patients of implementing the SLATE algorithm, compared to standard care.
11. Estimate costs to providers of implementing the SLATE algorithm, compared to standard care.

5. Study Design

5a. Overview

The study will be an individually randomized evaluation that will collect data from consented study subjects using questionnaires, a brief physical examination, a venous blood draw where not already done under routine care, and review of routine medical records. Patients randomized to the intervention arm will be initiated on ART if eligible under SLATE or referred for standard care if not eligible under SLATE. Health facility information that does not pertain to human subjects (e.g. unit costs) will also be collected. Figure 2 summarizes study design and procedures.

For peer review only

For peer review only - http://bmjopen.bmj.com/site/about/guidelines.xhtml

Figure 2. SLATE study procedures

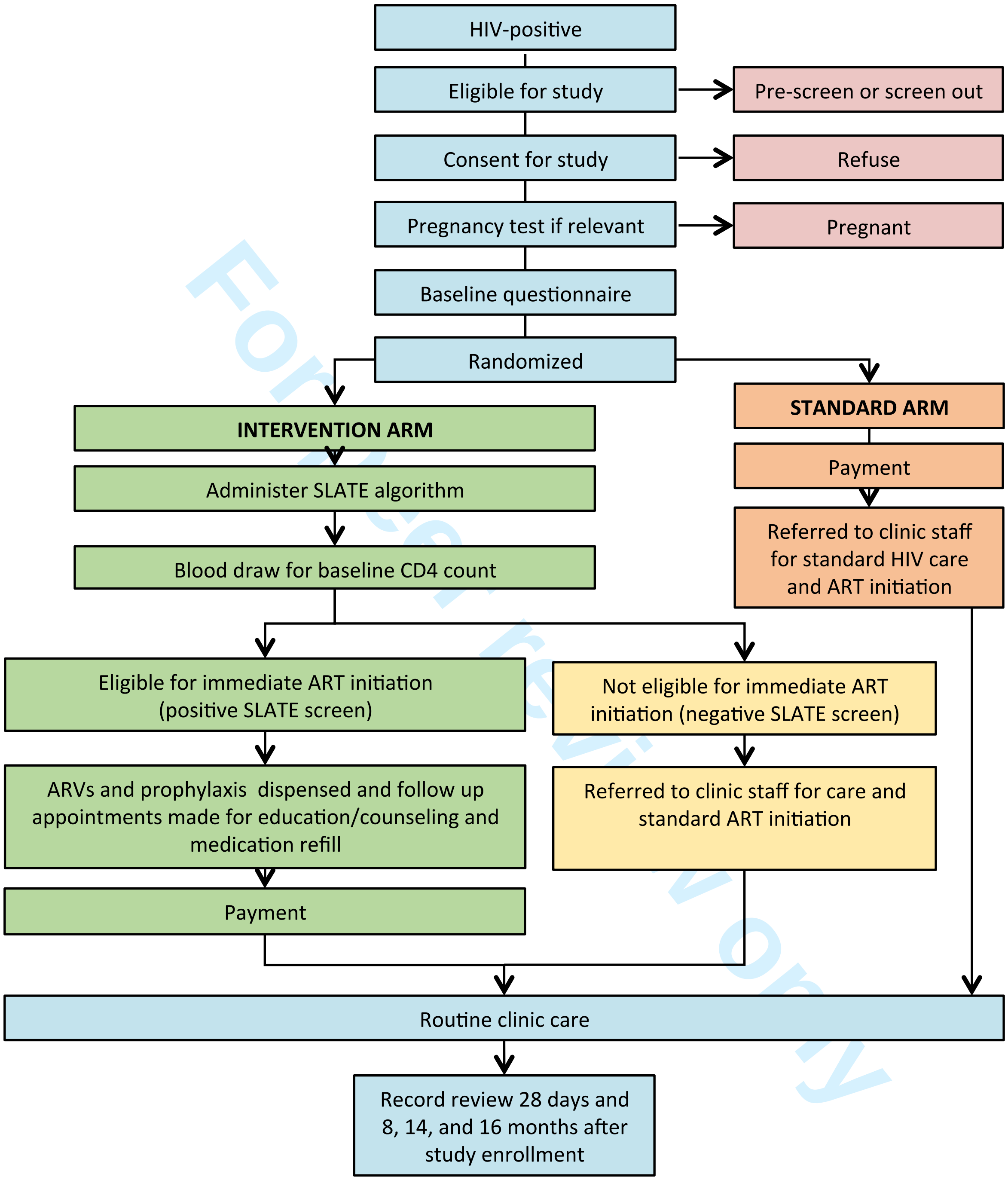

5b. Study sites

The study will be conducted at three high-volume, public sector clinics in each study country. We selected sites that have both a relatively high volume of potentially study-eligible patients, to reduce the time required for enrollment, and have service delivery infrastructure and staff that are typical of the public sector, so that results will be broadly generalizable within each country.

For peer review only - http://bmjopen.bmj.com/site/about/guidelines.xhtml

Sites were selected in collaboration with local study teams and in consultation with government health authorities in each area. A letter confirming government authorization for each site to participate in this study will be submitted to the BU and local IRBs prior to study enrollment at that site.

The proposed sites in each country are:

South Africa:

- OR Tambo Primary Health Clinic, City of Johannesburg
- Alexandra Community Health Centre, City of Johannesburg
- Jabulani Dumani Community Health Centre, Ekhurhuleni Metro

Kenya:
- Kericho County Referral Hospital, Kericho
- Kapsabet County Referral Hospital, Kapsabet
- Kombewa County Hospital, Kombewa

5c. Study population

The study population will be all non-pregnant HIV-infected adults who are not yet on antiretroviral therapy.

Inclusion criteria:
- Adult patients ($\geq$18 years) (initiating children on ART is likely to require additional information, making the SLATE algorithm less applicable to pediatric populations)
- Confirmed HIV-positive test result at any time (may have been diagnosed previously)
- Not currently on ART (three-drug combination)
- Presented at the study clinic for any HIV-related reason, including an HIV test, pre-ART monitoring, or ART initiation

Exclusion criteria:
- Pregnant (pregnancy is an exclusion criterion because treatment guidelines for pregnant women differ from those for non-pregnant adults; most pregnant women are diagnosed with HIV and initiated on ART in antenatal clinics, not general adult HIV clinics)
- Not intending to return to this clinic for further HIV care in the coming year (i.e. intends to seek further care somewhere else)
- Not physically, mentally, or emotionally able to participate in the study, in the opinion of the investigators or study staff
- Not willing or able to provide written informed consent to participate in the study
- Previously enrolled in the same study

All the study countries have adopted a policy of “treat all” or “test and treat”, so that all SLATE study subjects will also be eligible for ART under national guidelines. Although implementation of this policy may not be complete and may vary by site, we will enroll all patients who would be eligible under expected treat all guidelines regardless of actual clinic-specific practice at the time of the study. This will

For peer review only - http://bmjopen.bmj.com/site/about/guidelines.xhtml
For peer review only

ensure that the SLATE study can generate information for a future in which “treat all” is the standard in most places.

Patients will be followed passively through medical record review for up to 16 months after study enrollment.

6. Enrollment and Preparation

6a. Pre-screening

Patients presenting at a study clinic who are HIV-infected but not yet on ART will be eligible for study pre-screening. This includes patients diagnosed earlier and patients newly diagnosed on the same day. At each study site, the site’s staff (counselors, clerks, and clinical staff, as appropriate to each site) will inform potentially eligible patients about the study at visit registration, which will be defined as the patient’s first point of contact with clinic staff upon arriving at the clinic. Patients who express willingness to learn more about the study will be referred to a study assistant for screening and consent. Patients will be assured that they will not “lose their place” in the clinic queue by agreeing to referral to the study assistant; upon completion of interaction with study staff, the study assistant will escort patients who do not want to participate or who were randomized to standard care to return to their original place in the queue (or front of the line in case their place was lost) to continue with their routine clinic visit.

Where feasible, clinic staff who refer patients to the study assistant will be asked to complete one row in a pre-screening logbook (Appendix 1) for each patient they inform about the study, to allow the study to collect aggregate statistics about the overall patient population and compare it to the screened population. Fields that will be recorded in this logbook are date, sex, age in years, and primary reason for clinic visit (HIV test, CD4 count, pre-ART monitoring, adherence education session, ART initiation). No identifiers of any kind will be collected in this logbook, which is intended to provide only aggregate information. We anticipate that use of the logbook will be feasible at some study sites and some first points of contact at those sites but may not be feasible at others, if clinic staff are too busy or do not remain in a fixed location in the clinic. Where the logbook cannot be used, clinic staff will follow the same procedures for information patients about the study, but no record will be kept. Data analysis requiring pre-screening data will be limited to sites where the logbook is used.

6b. Screening

Upon referral to the study assistant, patients will receive a more complete description of the study, including the details of why it is being done, study procedures, and the need for written informed consent. They will be assured that participation is voluntary and that they can withdraw from the study at any time, without affecting the quality of care provided by the site. They will also be offered the opportunity to ask questions. If the study assistant concludes that all other inclusion/exclusion criteria have been met, patients will then be asked to provide written informed consent to participate, as described below. The study assistant will complete a screening form (Appendix 2) to confirm study eligibility for each patient screened. The screening form will not collect any identifiable information pertaining to individual patients prior to receipt of written informed consent.

Patients will be screened consecutively, as they arrive at the study site. Due to the volume limitations of study staff, however, recruitment may be halted during intervals when the study assistant and/or study

For peer review only - http://bmjopen.bmj.com/site/about/guidelines.xhtml

clinician are already busy. We anticipate that clinical data collection will take no more than 30 minutes per patient, allowing us to enroll and collect data from roughly 8-10 patients per day using a single study clinician per site.

6c. Consent

Written informed consent will be obtained from all study participants after screening and prior to study enrollment. The study information sheet and consent form (Appendix 3) will be translated into local languages most commonly spoken by patients at the study sites. Translated consent documents and attestation of consent accuracy will be submitted to the BUMC IRB and local IRB prior to use with any study subjects. Potential subjects will be asked if they would prefer to read the information sheet or have it read to them by the study assistant and will be given the opportunity to ask questions about the study. Those who provide written informed consent will be enrolled. Those who refuse consent will be referred back to the clinic staff to complete their routine visit, and the study assistant will indicate the refusal on the screening form.

6d. Pregnancy test

Because SLATE is not intended for pregnant women, we will conduct a rapid pregnancy test for female patients following consent. Women who have already reported being pregnant will have been screened out prior to consent; the pregnancy test will identify those who were not aware of being pregnant. Patients who have a positive pregnancy test will be withdrawn from the study at this time and escorted to the clinic registration desk to begin antenatal care and PMTCT.

6e. Questionnaire

The study assistant will administer a short questionnaire documenting the subject's basic demographic and socioeconomic characteristics, costs incurred per clinic visit, and preferences regarding ART initiation. As the questionnaire elicits only simple responses and will be completed by the study assistant not the subject, we do not intend to translate the questionnaire itself into subjects' first languages. Instead, for subjects who do not speak English, the assistant will ask the questions in one of the commonly-understood languages used by the study population, and then record the answers in English on the questionnaire. The questionnaire is included in the study case report form in the appendix to this protocol.

6f. Randomization

After the questionnaire has been completed, patients will be randomized to the intervention or standard groups of the study. Randomization envelopes will be generated in blocks of 6 and kept at the study sites. For each enrolled patient, the study assistant will open the next envelope in sequential order, read the randomization group, and record it on the case report form. The study assistant will then inform the patient of the randomization assignment.

Following randomization, standard group patients will be escorted to the clinic registration desk to resume their routine clinic visit. The study assistant will repeat the explanation of next steps in the study, which are limited to review of medical records by the study team, provide study payment as described below, and thank them for their willingness to participate in the study. The study team will have no further personal contact with patients in the standard group.

For peer review only - http://bmjopen.bmj.com/site/about/guidelines.xhtml
For peer review only

The study assistant will introduce intervention group patients to the study clinician for algorithm data collection. The study clinician will be a nurse, medical officer, or clinical officer at the same level as the individual responsible for ART initiation under standard care at the study site.

7. Intervention

7a. Algorithm data collection

The SLATE algorithm (illustrated in Figure 1 above) calls for four types of screens: a symptom report, medical history, brief physical exam, and readiness assessment. Each of these is described below and further detail is provided in the case report form appended to this protocol. A negative response to a screen indicates that the patient “screens out” and referall for additional care (consultation, laboratory test, counselling, other) is recommended; if all responses are positive, then the patient “screens” in for immediate initiation under SLATE.

1. Symptom report. Each patient will be screened for current cough, fever, night sweats, and weight loss, which comprise the standard, WHO-recommended TB symptom screen[8], and for persistent (continuous) headache for more two days, which is a symptom of cryptococcal meningitis[9,10], and will be asked about any other symptoms that the patient has noticed.

2. Medical history: To identify individuals who are likely to face problems with medication-taking or adherence, patients will be asked if they have ever been on ART previously, started TB treatment in the past two weeks, are taking concurrent medications for epilepsy or warfarin, which can interact with ARVs, or have a history of substance abuse.

3. Physical exam: A brief, symptom-guided physical exam to look for causes of symptoms or any other evident conditions that could postpone ART initiation will be conducted.

4. Readiness assessment and counselling: A published review of readiness instruments for HIV initiation found that none was notably successful in predicting patient readiness for ART, as indicated by adherence once on treatment[11]. Despite this, there is general consensus that explicit attention to readiness is an important component of treatment initiation. Drawing on the published literature on stage-of-readiness scales[12], we have created a short instrument which we expect to serve primarily as an invitation to patients to raise concerns or questions about treatment readiness with the clinician conducting the assessment. Following the questions, the study clinician will have a brief conversation with the patient to confirm that the patient feels ready to start treatment immediately, understands what happens next, and has no further questions or concerns.

If SLATE were used in routine care, we expect that the screens would be administered sequentially, so that a negative response on the first screen would cause a patient to screen out without having to complete the second screen, and so on. Patients would progress to the next screen only after being found positive on the previous one. For purposes of this study, however, we will administer all four screens, to obtain a complete algorithm data set for each patient in the intervention arm.

We anticipate that for the vast majority of patients, the first three screens will take less than 5 minutes each to administer and the last screen less than 10 minutes. Patients who screen out will require more time, and we therefore estimate needing an average of up to 30 minutes of clinician time per patient in the intervention am.

For peer review only - http://bmjopen.bmj.com/site/about/guidelines.xhtml

7b. Blood draw

Cryptococcal meningitis and other co-infections are more likely in patients with very low CD4 counts. Although new national guidelines in the study countries do not require the results of a CD4 count to establish treatment eligibility, most countries will continue to use CD4 counts to monitor patient condition. To avoid asking intervention group patients to wait in the clinic's queue for a blood draw, the study clinician will do a standard venous blood draw, following the same procedures that the clinic would otherwise use. Blood samples will be tested by the laboratories that serve the clinics, following routine procedures, and results will be placed in routine medical records so that the study clinics do not have to repeat the CD4 count for the same patients. The clinic may also use the blood sample for other baseline tests recommended in national guidelines, such as creatinine, hemoglobin, and for patients with CD4 counts under 100 cells/mm^3, cryptococcal antigen (CrAg). The clinic will follow routine procedures for laboratory tests; the study will only be responsible for drawing the required blood sample.

Study staff will monitor CD4 count results for all patients enrolled in the intervention arm. Under national guidelines in both countries, any patient who has a CD4 count under 100 cells/mm^3 and a positive cryptococcal antigen (CrAg) screening result should begin presumptive treatment for cryptococcal meningitis. Results of the CD4 count and CrAg screening test will only be available several days after study enrollment, however. To facilitate proper follow up of any intervention arm patient who does have a positive CrAg screening test, study staff will review CD4 count results for intervention arm patients as soon as the results are available at the study clinics. The study team will alert the clinic to any patient who has a CD4 count under 100 and request that the clinic trace the patient and provide follow up care as guidelines mandate.

7c. Referral of patients who screen out

After completing all four of the SLATE screens, the study clinician will fill out a brief referral letter that reports any substantive findings, such as TB symptoms or a physical exam observation. Intervention arm patients who screen out (have negative responses on any of the SLATE screens) will be referred to routine clinic care to address the issue(s) identified and initiate ART under standard care. The study clinician will discuss and explain the concern(s) with the patient and then escort the patient to the registration desk to continue with a standard clinic visit. The patient will be given the referral letter and encouraged to give the letter to the clinic nurse or medical officer. Patients who do not wish study findings to be shared with clinic staff will not be required to pass on the letter. Patients who are referred to routine care due to a positive SLATE screen will remain in the intervention arm, have the same study follow up as other intervention arm participants, and be analyzed as part of the intervention arm.

For patients who screen in (have positive responses on all the SLATE screens and are eligible for immediate ART initiation) give their agreement, referral letters will be placed in their clinic files along with other source documentation, such as the regular ART record that must be filled out for all new ART patients. Letters will not be filed for patients who do not agree.

7d. Dispensing of ARVs and prophylaxis

Patients who have a full negative SLATE screen (no reasons for delay) will immediately be offered their first supply of ARV medications. While making the offer to dispense, the study clinician will confirm that

For peer review only - http://bmjopen.bmj.com/site/about/guidelines.xhtml

the patient still wishes to start ART immediately, answer any questions the patient may have, and reiterate the importance of returning for the patient's next appointment and medication refill.

In addition to ARVs, national guidelines call for prescribing isoniazid preventive therapy (IPT) and/or co-trimoxazole prophylaxis to new HIV patients. The study clinician will also write prescriptions for these medications. Where possible, the study clinician will have a stock of medications in the study room, so that the patient does not need to go anywhere else in the clinic to obtain their medications. If this is not allowed, the study clinician or study assistant will escort the patient to the clinic pharmacy to obtain the medications without waiting in a queue. The clinician or study assistant will then escort the patient to the clinic registration desk to schedule the next appointment, which will include participation in one of the clinic's routine adherence education sessions.

7e. Completion of direct study interaction and payment

Direct study interaction with study subjects will cease as follows:

- *Intervention group (Track 1)*: Upon completion of the ART initiation steps described above, after which subjects will be managed by the study site following standard procedures.

- *Standard group (Track 1)*: after randomization, at which point subjects will be managed by the study site following standard procedures.

From these points on, all subjects, regardless of which type of initiation they received, will be managed by the site following its routine procedures for pre-ART and ART care. Because this study evaluates routine practice and uses retention in care as an outcome, any further study interaction with subjects would affect the results, in particular loss to follow-up. There will therefore be no post-baseline study visits, only routine care visits not involving study personnel. Standard procedures will also be implemented by the study site (not the study team) for study subjects who miss scheduled visits.

The last step in study interaction for both groups will be payment. Reimbursement to consented study subjects of the equivalent of $5-$15 will be provided as a token of appreciate for study participation. The exact amount will be determined in consultation with the study sites and local study teams. The reimbursement will be in the form of cash in Kenya and a voucher to a nearby supermarket and general goods store in South Africa.

8. Medical Record Follow Up

Study subjects in both arms will be followed passively through review of routine medical records kept by the study sites for up to 14 months after study enrollment. This will capture up to 1 month for treatment initiation, up to 12 months of follow up, and up to 1 month for a 12-month routine visit to be made.

The purpose of medical record follow up is to determine which patients enrolled in the study did go on to initiate ART and after what time interval, their early outcomes on treatment, and resource utilization (cost) of initiating ART.

Specific data fields to be collected from medical records are listed under Data Collection below.

For peer review only

9. Data Collection and Management

9a. Sources of data

The study will collect data from five sources, as follows:

1. Pre-screening logbook. Aggregate statistics about the number of potentially eligible patients not screened for the study will be calculated from logbooks completed by clinic staff.

2. Screening form. Screening forms will be completed to confirm study eligibility and consent.

3. Case report form. The study assistant and study clinician will use the CRF to record all information gathered directly from the patient, including the questionnaire and four SLATE screens.

4. Medical records. Routinely collected medical record data will be abstracted from medical records maintained by the study site and by central repositories (e.g. laboratory database) in electronic and paper format.

5. Facility-level records. We will use aggregate information from the study sites to estimate the overall proportion of patients presenting at each clinic who could start ART immediately if SLATE were used in routine care and the resources that would be required.

9b. Pre-screening logbook

As explained above, where feasible we will ask clinic registration staff to complete one row of a pre-screening logbook for each patient who is offered referral to the study assistant for screening. The logbook will have only five fields: date of visit, sex, age in years, primary reason for clinic visit (HIV test, CD4 count, pre-ART monitoring, adherence education session, ART initiation), and whether the referral to the study assistant was accepted (yes/no). No identifiers of any kind will be collected in this logbook, which is intended to provide only aggregate information. Data from the logbook will be used to estimate the total volume of patients who could benefit from SLATE if it were adopted as standard care and to compare the characteristics of patients who do enroll in the study with those of the overall patient population at the site.

9c. Screening form

The study assistant will complete a screening form for each patient referred after pre-screening. The screening form will record whether the patient meets each inclusion and exclusion criterion and provides written informed consent. For patients enrolled, the Study ID number and medical record number will be added to the screening form, which will become part of the case report form for study subjects. For patients who screen out, it will record the reason for ineligibility.

For female patients, the results of the pregnancy test conducted after consent will be recorded on the screening form, as pregnancy is an exclusion criterion for the study. No case report form will be opened for patients who have positive pregnancy tests.

9d. Case report form

For peer review only - http://bmjopen.bmj.com/site/about/guidelines.xhtml

The case report form will have eight sections. Sections 1, 7, and 8 will be completed for standard group patients. All sections will be completed for intervention group patients.

1. A short, closed-ended questionnaire to be administered by the study assistant after consent. The questionnaire will elicit basic information about:

    - Patient characteristics (age, sex, marital status, household composition, duration living in current location, education)
    - HIV-related information (familiarity with clinic, time of diagnosis, acquaintance with others with HIV or on ART)
    - Activities and employment
    - Costs to patient of making a clinic visit (transport costs, loss of income, etc.)
    - Patient's preferences regarding ART initiation (single visit, multiple visits, intervals between).

2. Symptom report. The symptom report form will record yes/no to the presence of the symptoms listed above and provide a space for the study clinician to describe or comment on symptoms and to state whether the patient is ineligible for immediate ART initiation due to symptoms.

3. Medical history. The medical history form will record answers to the medical history questions and provide a space for the study clinician to describe or comment on these answers and to state whether the patient is ineligible for immediate ART initiation due to medical history.

4. Physical exam. The physical exam form will record findings of the physical exam and provide a space for the study clinician to describe or comment on these findings and to state whether the patient is ineligible for immediate ART initiation due to the outcome of the medical exam.

5. Readiness assessment. The readiness assessment form will record answers to the readiness questions and provide a space for the study clinician to describe or comment on these answers and to state whether the patient is ineligible for immediate ART initiation due to readiness.

6. Blood draw. The blood draw form will confirm that a blood sample was taken and record the identification number or bar code used to identify the sample on the CD4 count laboratory order form.

7. Patient identifiers. The identification form will capture identifying information about the subject, including name, date of birth, clinic ID number, and national identification number. This information will not be entered into the study database but will be used only for matching CRF and medical record data.

8. Completion form. The last page of the CRF will confirm data collection and close the study visit for the patient.

9e. Medical record data

Once the CRF has been completed, all remaining patient-level data for the study will be collected from routinely maintained medical records. The format of these records will vary by study country and site. Some sites will use electronic medical records, while others will rely on paper-based records and registers or a combination of electronic and paper. We will access all available records for each study

For peer review only - http://bmjopen.bmj.com/site/about/guidelines.xhtml

patient, regardless of format or storage location, to try to obtain as complete a study data set as possible. The fields we will aim to capture are listed below.

To be used to link electronic and paper-based records to study identification number only:

- Electronic medical record system number
- Clinic file numbers
- National ID number
- Name
- Date of birth
- Sex

All subjects at clinic visit on date of study enrollment:

- Date of positive HIV test (if available)
- CD4 count (most recent)
- Date and result of TB symptom screen, if done
- Date, type, and result of TB test(s) (current and previous, if any)
- TB treatment, if offered (date of initiation)
- WHO stage and clinical conditions

Subjects who are eligible for ART initiation at the study clinic:

- Reasons for delaying ART initiation, if relevant
- Date ART initiated (first dose of drugs dispensed)
- First-line regimen prescribed

For all clinic visits for duration of study follow up, from study enrollment to the follow-up endpoints indicated above:

- Date of visit
- Primary reason for visit (e.g. pre-ART visit, scheduled medical visit, medication pickup, unscheduled medical visit)
- Types of professionals seen (e.g. nurse, doctor, pharmacy assistant, counselor)
- Group and individual sessions attended (e.g. wellness course, adherence education)
- Numbers and types of all lab tests conducted (including CD4 counts, viral loads, CrAg test, blood tests to determine ARV regimen)
- Other procedures (e.g. x-rays) performed
- Medications prescribed and dispensed (ARV and non-ARV, including isoniazid and cotrimoxazole)
- Admissions for inpatient care since previous visit, if recorded
- Details of inpatient care received (facility, number of days, medications, lab tests, etc.) if recorded

At follow-up endpoints (6 and 12 months after ART initiation or comparable timepoint for those who do not initiate ART):

- Date and result of most recent viral load test
- Status at clinic (in care / died / transferred to another site / stopped care / lost to follow up, defined as > 1 month late for last medication pickup)
- New or recurring HIV-related conditions
- Date and cause of death if applicable
- Date and result of most recent CD4 count

For peer review only - http://bmjopen.bmj.com/site/about/guidelines.xhtml

9f. Study identification numbers and linking of records

Upon consenting to participate in the study, each subject will be assigned a random study identification number. This will be a five-digit number in which the first digit indicates the country and the remaining four digits will be a sequential number that does not provide any identifying information.

A password-protected, encrypted linking file will be created in REDCap to link study identification numbers to subjects' names, dates of birth, national identity numbers, and clinic file numbers. These fields are needed to ensure that study ID numbers can be correctly matched to the identification numbers used in the sites medical records. When a subject has been matched to a clinic record, the clinic record number will also be added to the linking file. The linking file will be a separate document from any other study files. Access to the linking file will be limited to the study team, and the file will be stored in a secure location separate from the study database.

9g. Data entry and storage

Screening and CRF data will be entered by study staff onto Android-based tablet computers programmed for data collection using the REDCap mobile app. The REDCap system provides a secure, web-based platform for data entry that allows real time monitoring, querying, and quality control of data by an off-site data manager. It allows access to data to be assigned and restricted to individual study staff as needed and creates audit trails to monitor appropriate access. The mobile app allows data to be entered offline and then uploaded to a central server when an internet connection is available. Records are encrypted upon entry and can be removed from data capturers' tablets once they have been uploaded to the server, reducing the chances of breach of confidentiality if a tablet is lost or stolen. REDCap is supported by the Boston University IT office (https://redcap.bumc.bu.edu/). It has previously been used successfully in South Africa, where the University of the Witwatersrand is a REDCap Consortium partner.

Medical record data for study subjects will be downloaded from electronic medical record systems where available. The lead data analyst will be responsible for ensuring that study identification numbers are correctly matched to the record numbers in the database. In instances where the data analyst cannot match a study ID number to an electronic medical record number using only the fields in the study linking file, a query will be sent to the on-site study staff, who will access the clinic's paper-based patient files to determine whether the subject ever initiated ART and, if so, locate the correct medical record number.

At sites where all or some data are recorded only in individual paper-based files, a note or tab indicating that a patient is a participant in the study will be attached to the cover of each subject's file, to assist site and study staff in locating study subjects' files for data extraction. At regular intervals, files will be pulled and the data fields listed above entered into the study database by study staff. All data entry will take place at the study clinics or other locations where the files are stored; no hard copies of patient records will be taken off site by the study team.

Data will be retained in secure study databases until completion of the study, including completion of all data analysis and report writing associated with the study and closing of the IRB-approved protocol. Study data sets and other electronic files containing study data will then have all identifiers removed

For peer review only - http://bmjopen.bmj.com/site/about/guidelines.xhtml

and where possible will be made available for use of other researchers through a repository such as Dryad. Hard copies of study documents will be shredded.

10. Staff Supervision and Training

In each study country, the local study team will include a principal investigator, study coordinator, and site-based study clinicians, assistants, and data capturers. The local PIs will be responsible for training and supervising their teams, under the guidance of the overall study PI and co-investigators. The study clinicians who will implement the intervention will have the qualifications required for ART initiation in each study country. Study assistants who will administer consent and the questionnaire will also be trained as HIV counselors to ensure that they are sensitive to the needs of HIV-positive study subjects.

All members of the study team will be trained in and adhere to the standards laid out in the Belmont Report and NIH guidelines for research and in research ethics applicable to South Africa. Human subjects ethics training will be repeated on an annual basis to ensure that the study team's understanding of research ethics is current.

11. Sample Size

Using results of the RapIT randomized trial we recently conducted in South Africa, we estimate that 65% of treatment-eligible patients will be initiated on antiretroviral therapy and retained on ART in the standard group, and we consider an increase to 80% to be programmatically important. Using an α of 0.05, power of 90%, 1:1 randomization, and an uncorrected Fisher's exact test, this requires a minimum sample size of 197 patients per group, which we will increase to a maximum of 240 to ensure sufficient power if patients withdraw or are found ineligible after consent. The sample size will thus be a maximum of 480 per country, or a maximum of 960 for the two-country study.

12. Outcomes and Data Analysis

12a. Outcomes

The primary outcomes for the study are:

1. The proportion of patients initiated on ART within 28 days of study enrollment. While national guidelines recommend ART initiation within two weeks of a patient's first clinic visit or HIV diagnosis, recent studies suggest that 28 days is a more realistic deadline for routine care.

2. The proportion of patients who initiate ART and are alive, in care, and retained on ART eight months after study enrollment. Eight months was selected to allow up to 1 month (28 days) to initiate ART, six months of follow up after treatment initiation, and up to 1 month to return for the six-month routine clinic visit.

These outcomes were proposed in a paper published by the investigators based on the MATI technical consultation described in protocol section 3c.[7]

Secondary outcomes will include:

For peer review only - http://bmjopen.bmj.com/site/about/guidelines.xhtml

3. The proportion of patients initiated on ART within 14 days of having an HIV test or enrolling in HIV care.
4. The proportion of patients who initiate ART and are virally suppressed eight months of having an HIV test or enrolling in HIV care between HIV-infected patients offered immediate ART initiation under the SLATE algorithm and patients offered standard ART initiation.
5. The proportions of patients who initiate ART and are alive, in care, and retained on ART 14 and 16 months after having an HIV test or enrolling in HIV care. Fourteen months extends the primary outcome endpoint, which is at 8 months, by an additional six months. Sixteen months allows loss to follow up at 12 months to be defined as three months late for the 12-month visit, which is a common endpoint in the literature (16 = 1 month to initiate ART + 12 months' follow up + 3 months to attend 12-month visit).
6. The proportions of HIV-positive patients presenting at study clinics and not yet on ART who are eligible and ineligible for immediate initiation using SLATE algorithm criteria.
7. Reasons for ineligibility for immediate initiation, among those found ineligible in the intervention arm.
8. Average time to ART initiation (days) for each arm.
9. Patient preferences on the speed and timing of ART initiation.
10. Costs to patients of ART initiation under standard and intervention procedures.
11. Costs to providers of ART initiation under standard and intervention procedures.

12b. Analytic methods

We will first conduct descriptive analyses to allow us to determine the characteristics of the sample we have enrolled. We will then analyze the specific outcomes as follows:

Primary outcomes 1 and 2 and secondary outcomes 3, 4, and 5:

For all primary and secondary clinical outcomes, the analytic approach will be the same. All analyses will be by intention-to-treat: subjects will be analyzed according to the intervention they were supposed to receive, whether or not they adhere to the defined intervention. This includes patients randomized to the intervention arm who are screened out of immediate ART initiation by the SLATE algorithm; they will remain in the intervention arm for data analysis. Primary analyses will be pooled across study countries, though we will also conduct stratified analyses by country to look for country-specific differences.

The analysis will begin with a simple comparison of the two treatment groups with respect to baseline predictors of outcomes to look for any imbalances. These potential confounders include demographic (e.g. age, sex) and socioeconomic (education, distance from clinic, employment, etc.) variables, medications, baseline CD4 counts, and low BMI, among others.

We will then conduct a crude analysis comparing the proportion of patients achieving each dichotomous outcome by group and estimating crude risk ratios and crude risk differences and their corresponding 95% confidence intervals. This will be our primary analysis as we anticipate that randomization of over 1000 subjects should lead to balance in baseline covariates. Should any important imbalances occur by chance, we will proceed with an adjusted model as described below for the country-specific analyses.

Next, we will look for effect modification by important predictors of each outcome. The primary modifier will be country, as practices for care and treatment may differ across the different countries.

For peer review only - http://bmjopen.bmj.com/site/about/guidelines.xhtml

We will also look for differences in effects stratified by age, sex, baseline BMI, CD4 count, and any other important demographic and clinical predictors of outcomes identified. Our analysis for effect modification will use a simple stratification of the primary analysis by the potential modifier and report crude risk differences and risk ratios and their corresponding 95% confidence intervals.

When stratified by these potential effect modifiers, because the sample sizes within groups will be smaller, it is possible that some baseline covariates may be imbalanced by treatment arm. To adjust for potential differences by baseline covariates, a log-linear regression model will be used to estimate adjusted risk ratios. Variables considered to be important in the univariate stage will be included in multivariate models. Adjusted analyses will include covariates which are unevenly distributed across treatment groups and which could plausibly affect retention or ART initiation. Each of these models can then be used to look for patient-level predictors of treatment uptake and retention in care.

Secondary outcomes 6-9:

Secondary outcomes 6, 7, 8, and 9 are descriptive in nature. These will be described using frequencies, stratified by country, site, and patient characteristics as numbers allow.

Secondary outcome 10:

We will estimate the average cost to patients per clinic visit using questionnaire responses, including transport, substitute labor, and other costs. This cost will then be multiplied by the average number of clinic visits observed in each study arm to generate a total average cost to patients of ART initiation under each model (SLATE and standard). Costs will be reported as means (standard deviations) and medians (IQRs) in local currencies and dollars.

Secondary outcome 11:

At 8 months after study enrollment, all resource usage will be extracted from subjects' medical records and case report forms. Unit costs will be obtained from external suppliers and the site's finance and procurement records and applied to the resource usage data to provide a cost per study patient. Costs will be measured from the provider perspective and will include the cost of all resources utilized for each study patient, including drugs, laboratory tests, outpatient visits, and fixed costs such as building space, equipment, and management staff. We will estimate the average cost to the provider per patient achieving each primary outcome. The average cost per outcome will then be compared between intervention and standard initiation groups to provide an estimate of the cost-effectiveness of the two strategies. Costs will be reported as means (standard deviations) and medians (IQRs) in local currencies and dollars.

12c. Dissemination of findings

The results of this study will be disseminated as widely as possible in the study countries, where new strategies for increasing the efficiency of treatment initiation are activity sought. A full report will be made to the study sites and other local stakeholders and will be circulated widely and posted on our website. We will also develop a short briefing document to send to the national and provincial departments of health, donor agencies, and other interested organizations, and we will present the results at relevant conferences domestically and internationally. One or more journal manuscripts will be submitted to an appropriate peer-reviewed international journal.

For peer review only - http://bmjopen.bmj.com/site/about/guidelines.xhtml
For peer review only

13. Human Subjects Considerations

13a. Risks and protection against risks

For this study, we will not collect any biomedical samples that would not be collected as part of routine care, nor will we implement any clinical procedures that are not routinely carried out under standard of care. The strategy being evaluated merely reorganizes data collection and accelerates the timing of events. The only non-routine data generated for the study itself will be responses to questionnaires. Study staff will conduct a brief physical exam and do a blood draw for intervention arm subjects, but both are procedures that are routinely performed and carry little risk. We therefore believe that our study poses no physical risks to subjects beyond those routinely encountered. It may, however, pose risks associated with loss to HIV care, emotional distress, and breach of confidentiality.

<u>Emotional distress</u>

Risk: By necessity, the study population for this study will include some patients who have been newly diagnosed with HIV. Interacting with them in order to explain the study and confirm eligibility before requesting written informed consent may cause some emotional distress for some potential subjects.

Protection: To minimize any emotional distress caused by interacting with study staff or enrolling in the study, we will train the site's staff to emphasize when they introduce the study to potential subjects that referral to study staff and enrolling in the study are completely voluntary and that those who do not wish to enroll will receive exactly the same care as the study site would otherwise have provided. Potential subjects will also be told that they can discontinue participation at any time, even after consenting. Study staff will be trained to look for signs of emotional distress and instructed to terminate or postpone the enrollment process if subjects appear distraught.

<u>Loss to HIV care</u>

Risk: The study aims to initiate on ART patients who might otherwise be lost to HIV care. Some patients lost before or after ART initiation will seek care at other sites, but many will not and will postpone or end all HIV care and treatment, some until they become more seriously ill, others forever. Although the SLATE strategy is expected to increase the proportion of patients who do initiate ART, there is a possibility that patients who accept immediate ART initiation under SLATE will subsequently discontinue treatment at a greater rate than do those who receive standard ART initiation. In our prior study of same-day initiation (RapIT), we found that a higher proportion of patients in the intervention arm than in the standard arm were lost to care, but we believe that most of these were patients who otherwise would never have started treatment at all, had only standard initiation been offered. We therefore expect the SLATE intervention to have a net positive impact on our primary outcomes. We cannot be certain of this result in advance, however.

Protection: Because this is an evaluation of routine practice and retention in care is a study outcome, no efforts will be made to retain patients in the study, beyond those already routinely undertaken by the study site clinics as part of standard care. Intervention arm patients will

For peer review only - http://bmjopen.bmj.com/site/about/guidelines.xhtml

be encouraged to remain in the clinic on the day of study enrollment if a routine ART adherence class or club is available on that day. Study staff will escort intervention arm patients to clinic registration desks to confirm that each patient has made an appointment for a follow-up visit and that the first follow-up visit includes the clinic's regular ART adherence activities (e.g. individual counselling, group education sessions, etc.). Through these efforts, intervention arm patients will have access to the same adherence support as standard arm patients, though the support will be offered after ART initiation rather than before.

Loss of confidentiality

Risk: Because we must collect identifying information in order to link CRF data with medical records, accidental disclosure of HIV status or other loss of confidentiality is also possible. A high level of stigmatization continues to inhibit the disclosure of HIV status in the study populations. Data collected in during the study visit or from clinic records could be disclosed and reveal a person's HIV status.

Protection: Several steps will be taken to protect subjects against the risk of accidental disclosure of HIV status. Individual documents or electronic files (including signed consent forms, case report forms, and the linking file) which could associate patients with an HIV study will be kept strictly confidential. Signed consent forms will be stored in locked cabinets away from the study site, with access limited to the senior investigators. CRFs will be held in electronic versions only and will be password-protected. The linking file will also be password-protected, with access limited to study staff. It will be used only for the purpose of linking study identification numbers with clinic medical records.

To protect against other violations of confidentiality, study staff will be trained in expectations that they are not to disclose any information collected in the study to anyone outside the study team. All study staff will be required to pass an ethics certification course, such as the on-line certification offered by the NIH. All study participants will be encouraged to contact the study clinician or clinic manager to report any undesirable conduct associated with the study. These reports will be brought to the attention of the local and international PI, and appropriate steps will be taken to solve the problems, including reporting to relevant ethical review boards.

13b. Benefits

Direct benefits

Study subjects offered immediate treatment initiation will benefit from the opportunity to start ART quickly and with fewer clinic visits and time delays than they would otherwise have incurred. Study subjects offered standard initiation will not receive any direct benefits from the study but will receive the same care that would have been provided in the absence of the study.

Indirect (societal) benefits

The study has the potential to generate substantial indirect benefits to the subjects. The research we will undertake is expected to reveal whether a simplified clinical algorithm can successfully be

For peer review only - http://bmjopen.bmj.com/site/about/guidelines.xhtml

incorporated into procedures for ART initiation, leading to more patients initiating ART and a reduction in patient attrition from care. The results of the study may thus lead to improvements in HIV care and keep patients alive and in care longer.

Ratio of benefits to risks

The knowledge to be generated by this study will allow HIV treatment programs to determine whether a simplified clinical algorithm can be used to determine eligibility of immediate ART initiation. This study will address an important gap in the current knowledge around ART initiation and what can be done to reduce the already high proportion of patients who are being lost from care after testing positive. There is currently almost no information available on how to reduce these pre-ART losses. As explained in the previous section, we believe that the risk to subjects in our study is minimal and is outweighed by the indirect benefits and the potential importance of the findings.

13c. Costs and payments to subjects

There will be no costs to subjects for participating in this study.

Subjects who consent to participate and complete study procedures will receive a payment of $10-$15 (local currency equivalent) to reimburse for their time and inconvenience. This payment will be made in the form of a shopping voucher, cell phone air time, or cash, depending on individual study site circumstances.

13d. Recruitment

Identification of potential subjects will occur during patients' regular clinic visits for any HIV-related service other than monitoring of patients already on ART. At reception and relevant waiting areas at each study site a flier will be posted that informs patients that a study is underway and indicates where to get further information. The flier will be translated into the commonly used languages at the study sites.

The site's post-test counselors, nurses, and registration staff and the study assistant will inform potentially eligible patients about the study while they are awaiting and/or receiving services. They will explain that a study is underway and that patients found eligible are invited to participate. They will be assured that they will not lose their place "in line" at the clinic and will still receive whatever services they came to the clinic for. Patients who indicate that they may be interested in participating in the study will be referred to the study assistant for screening.

13e. Informed consent

Written, informed consent will be sought from all study subjects by a trained study assistant. The informed consent information sheet will describe the nature, goals, and procedures of the study and assure subjects their information will be kept confidential. The consent form will explain:

- That patients will not get to decide which group they will be in, but rather that we will decide by chance and they will have a 50-50 chance of being in either group;
- How study procedures will be different from standard care procedures for those randomized to the intervention group;

For peer review only - http://bmjopen.bmj.com/site/about/guidelines.xhtml

- How we will do follow-up through medical record data collection; and
- That any individual patient can choose not to participate in the study and be offered standard initiation.

The full informed consent information sheet and form will be translated into the most commonly used languages at the study sites. Translated forms and attestations of translation accuracy will be submitted to the IRB prior to any use of the forms. Subjects who unable to read and/or sign the consent form due to illiteracy will be asked to provide a thumbprint mark, in the presence of a witness who will also sign the form. Patients who do not speak English or any of the languages into which the form has been translated will be regarded as unable to provide written informed consent, which is an exclusion criterion for the study, and will not be eligible for study enrollment.

13f. Protection of confidentiality

Subject data will be captured electronically on case report forms programmed on Android tablet computers using REDCap or another similar, secure application. The tablets will be password-protected and files will be encrypted. When not in use, they will be stored in locked cabinets at the study sites or in study team offices. Study subjects will be assigned sequential study ID numbers upon consent, and data files will contain study ID numbers only, without any other individual identifiers.

A password-protected linking file allowing medical record data to be matched to study-generated data will be maintained in a secure location, with access limited to study staff. Medical record data will be downloaded or extracted from paper records on a regular basis, as needed for data analysis. Electronic medical record datasets may include data on all patients at the study clinic, not just those included in the study, as it may not be possible to select only study participants prior to downloading. As soon as datasets are downloaded, the linking file will be used to select study participants and link their records with their study identification numbers. All identifiers will then be stripped from the clinical data and records for all patients not enrolled in the study will be deleted.

14. References

1. World Health Organization. Guideline on when to start antiretroviral therapy and on pre-exposure prophylaxis for HIV. World Health Organization, Geneva, 2015.
2. Barnabas R, Bendavid E, Bershteyn A, et al. HIV mortality by care cascade stage and implications for universal ART eligibility. Conference on Retroviruses and Opportunistic Infections, Boston, MA, Feb 22-25, 2016.
3. Rosen S, Fox MP. Retention in HIV care between testing and treatment in sub-Saharan Africa: a systematic review. PLoS Med 2011; 8: e1001056.
4. Lahuerta M, Ue F, Hoffman S, et al. The problem of late ART initiation in Sub-Saharan Africa: a transient aspect of scale-up or a long-term phenomenon? J Health Care Poor Underserved 2013; 24: 359–83.
5. Rosen S, Maskew M, Fox MP, et al. Initiating antiretroviral therapy for HIV at a patient's first clinic visit: the RapIT randomized controlled trial. PLoS Med 2016; 13: e1002015.
6. Lessells RJ, Mutevedzi PC, Cooke GS. Retention in HIV care for individuals not yet eligible for antiretroviral therapy: rural KwaZulu-Natal , South Africa. J Acquir Immune Defic Syndr 2011; 56: 79–86.
7. Rosen S, Fox MP, Larson B, et al. Accelerating the uptake and timing of antiretroviral therapy initiation in sub-Saharan Africa: an operations research agenda. PLoS Med 2016; 13: e1002106.

For peer review only - http://bmjopen.bmj.com/site/about/guidelines.xhtml

8. World Health Organization. Guidelines for intensified tuberculosis case-finding and isoniazid preventive therapy for people living with HIV in resource-constrained settings. World Health Organization, Geneva, 2011.
9. Wajanga BM, Kalluvya S, Downs JA, Johnson WD, Fitzgerald DW, Peck RN. Universal screening of Tanzanian HIV-infected adult inpatients with the serum cryptococcal antigen to improve diagnosis and reduce mortality: An operational study. J Int AIDS Soc 2011; 14: 48.
10. Aoussi EF, Ehui E, Demb JP, et al. Cryptoccocal meningitis and HIV in the era of HAART in Cote d'Ivoire. Med Mal Infect 2012; 42: 349–54.
11. Grimes RM, Grimes DE. Readiness: The state of the science (or the lack thereof). Curr HIV/AIDS Rep 2010; 7: 245–52.
12. Rathbun RC, Farmer KC, Lockhart SM, Stephens JR. Validity of a stage of change instrument in assessing medication adherence in indigent patients with HIV infection. Ann Pharmacother. 2007;41: 208–215.

12. Appendices

Appendix 1. Pre-screening log
Appendix 2. Screening form
Appendix 3. Informed consent information sheet and consent form
Appendix 4. Case report form
Appendix 5. Referral letter
Appendix 6. Recruiting flier

For peer review only

For peer review only - http://bmjopen.bmj.com/site/about/guidelines.xhtml

SPIRIT

STANDARD PROTOCOL ITEMS: RECOMMENDATIONS FOR INTERVENTIONAL TRIALS

SPIRIT 2013 Checklist: Recommended items to address in a clinical trial protocol and related documents*

Section/item	Item No	Description	Addressed on page number
**Administrative information**			
Title	1	Descriptive title identifying the study design, population, interventions, and, if applicable, trial acronym	________
Trial registration	2a	Trial identifier and registry name. If not yet registered, name of intended registry	________
	2b	All items from the World Health Organization Trial Registration Data Set	________
Protocol version	3	Date and version identifier	________
Funding	4	Sources and types of financial, material, and other support	________
Roles and responsibilities	5a	Names, affiliations, and roles of protocol contributors	________
	5b	Name and contact information for the trial sponsor	________
	5c	Role of study sponsor and funders, if any, in study design; collection, management, analysis, and interpretation of data; writing of the report; and the decision to submit the report for publication, including whether they will have ultimate authority over any of these activities	________
	5d	Composition, roles, and responsibilities of the coordinating centre, steering committee, endpoint adjudication committee, data management team, and other individuals or groups overseeing the trial, if applicable (see Item 21a for data monitoring committee)	________

For peer review only

**Introduction**			
Background and rationale	6a	Description of research question and justification for undertaking the trial, including summary of relevant studies (published and unpublished) examining benefits and harms for each intervention	______
	6b	Explanation for choice of comparators	______
Objectives	7	Specific objectives or hypotheses	______
Trial design	8	Description of trial design including type of trial (eg, parallel group, crossover, factorial, single group), allocation ratio, and framework (eg, superiority, equivalence, noninferiority, exploratory)	______
**Methods: Participants, interventions, and outcomes**			
Study setting	9	Description of study settings (eg, community clinic, academic hospital) and list of countries where data will be collected. Reference to where list of study sites can be obtained	______
Eligibility criteria	10	Inclusion and exclusion criteria for participants. If applicable, eligibility criteria for study centres and individuals who will perform the interventions (eg, surgeons, psychotherapists)	______
Interventions	11a	Interventions for each group with sufficient detail to allow replication, including how and when they will be administered	______
	11b	Criteria for discontinuing or modifying allocated interventions for a given trial participant (eg, drug dose change in response to harms, participant request, or improving/worsening disease)	______
	11c	Strategies to improve adherence to intervention protocols, and any procedures for monitoring adherence (eg, drug tablet return, laboratory tests)	______
	11d	Relevant concomitant care and interventions that are permitted or prohibited during the trial	______
Outcomes	12	Primary, secondary, and other outcomes, including the specific measurement variable (eg, systolic blood pressure), analysis metric (eg, change from baseline, final value, time to event), method of aggregation (eg, median, proportion), and time point for each outcome. Explanation of the clinical relevance of chosen efficacy and harm outcomes is strongly recommended	______
Participant timeline	13	Time schedule of enrolment, interventions (including any run-ins and washouts), assessments, and visits for participants. A schematic diagram is highly recommended (see Figure)	______

For peer review only - http://bmjopen.bmj.com/site/about/guidelines.xhtml

Sample size	14	Estimated number of participants needed to achieve study objectives and how it was determined, including clinical and statistical assumptions supporting any sample size calculations	______________
Recruitment	15	Strategies for achieving adequate participant enrolment to reach target sample size	______________

Methods: Assignment of interventions (for controlled trials)

Allocation:			
Sequence generation	16a	Method of generating the allocation sequence (eg, computer-generated random numbers), and list of any factors for stratification. To reduce predictability of a random sequence, details of any planned restriction (eg, blocking) should be provided in a separate document that is unavailable to those who enrol participants or assign interventions	______________
Allocation concealment mechanism	16b	Mechanism of implementing the allocation sequence (eg, central telephone; sequentially numbered, opaque, sealed envelopes), describing any steps to conceal the sequence until interventions are assigned	______________
Implementation	16c	Who will generate the allocation sequence, who will enrol participants, and who will assign participants to interventions	______________
Blinding (masking)	17a	Who will be blinded after assignment to interventions (eg, trial participants, care providers, outcome assessors, data analysts), and how	______________
	17b	If blinded, circumstances under which unblinding is permissible, and procedure for revealing a participant's allocated intervention during the trial	______________

Methods: Data collection, management, and analysis

Data collection methods	18a	Plans for assessment and collection of outcome, baseline, and other trial data, including any related processes to promote data quality (eg, duplicate measurements, training of assessors) and a description of study instruments (eg, questionnaires, laboratory tests) along with their reliability and validity, if known. Reference to where data collection forms can be found, if not in the protocol	______________
	18b	Plans to promote participant retention and complete follow-up, including list of any outcome data to be collected for participants who discontinue or deviate from intervention protocols	______________

Data management	19	Plans for data entry, coding, security, and storage, including any related processes to promote data quality (eg, double data entry; range checks for data values). Reference to where details of data management procedures can be found, if not in the protocol	______________
Statistical methods	20a	Statistical methods for analysing primary and secondary outcomes. Reference to where other details of the statistical analysis plan can be found, if not in the protocol	______________
	20b	Methods for any additional analyses (eg, subgroup and adjusted analyses)	______________
	20c	Definition of analysis population relating to protocol non-adherence (eg, as randomised analysis), and any statistical methods to handle missing data (eg, multiple imputation)	______________
**Methods: Monitoring**			
Data monitoring	21a	Composition of data monitoring committee (DMC); summary of its role and reporting structure; statement of whether it is independent from the sponsor and competing interests; and reference to where further details about its charter can be found, if not in the protocol. Alternatively, an explanation of why a DMC is not needed	______________
	21b	Description of any interim analyses and stopping guidelines, including who will have access to these interim results and make the final decision to terminate the trial	______________
Harms	22	Plans for collecting, assessing, reporting, and managing solicited and spontaneously reported adverse events and other unintended effects of trial interventions or trial conduct	______________
Auditing	23	Frequency and procedures for auditing trial conduct, if any, and whether the process will be independent from investigators and the sponsor	______________
**Ethics and dissemination**			
Research ethics approval	24	Plans for seeking research ethics committee/institutional review board (REC/IRB) approval	______________
Protocol amendments	25	Plans for communicating important protocol modifications (eg, changes to eligibility criteria, outcomes, analyses) to relevant parties (eg, investigators, REC/IRBs, trial participants, trial registries, journals, regulators)	______________

For peer review only - http://bmjopen.bmj.com/site/about/guidelines.xhtml

Consent or assent	26a	Who will obtain informed consent or assent from potential trial participants or authorised surrogates, and how (see Item 32)	________
	26b	Additional consent provisions for collection and use of participant data and biological specimens in ancillary studies, if applicable	________
Confidentiality	27	How personal information about potential and enrolled participants will be collected, shared, and maintained in order to protect confidentiality before, during, and after the trial	________
Declaration of interests	28	Financial and other competing interests for principal investigators for the overall trial and each study site	________
Access to data	29	Statement of who will have access to the final trial dataset, and disclosure of contractual agreements that limit such access for investigators	________
Ancillary and post-trial care	30	Provisions, if any, for ancillary and post-trial care, and for compensation to those who suffer harm from trial participation	________
Dissemination policy	31a	Plans for investigators and sponsor to communicate trial results to participants, healthcare professionals, the public, and other relevant groups (eg, via publication, reporting in results databases, or other data sharing arrangements), including any publication restrictions	________
	31b	Authorship eligibility guidelines and any intended use of professional writers	________
	31c	Plans, if any, for granting public access to the full protocol, participant-level dataset, and statistical code	________
**Appendices**			
Informed consent materials	32	Model consent form and other related documentation given to participants and authorised surrogates	________
Biological specimens	33	Plans for collection, laboratory evaluation, and storage of biological specimens for genetic or molecular analysis in the current trial and for future use in ancillary studies, if applicable	________

*It is strongly recommended that this checklist be read in conjunction with the SPIRIT 2013 Explanation & Elaboration for important clarification on the items. Amendments to the protocol should be tracked and dated. The SPIRIT checklist is copyrighted by the SPIRIT Group under the Creative Commons "Attribution-NonCommercial-NoDerivs 3.0 Unported" license.

For peer review only
